# Inhibition of Pellino-1 reverts the progression and tyrosine kinase inhibitor resistance in chronic myeloid leukemia

**DOI:** 10.1038/s41419-026-08799-7

**Published:** 2026-05-05

**Authors:** Qian Zhou, Guangsen Xu, Zhuoran Li, Yan Xu, Jianmin Guan, Zhenyu Li, Mingying Li, Tingjian Zu, Yuan Li, Chunhua Lu, Chunyan Ji, Baobing Zhao

**Affiliations:** 1 Key Lab of Chemical Biology (MOE), School of Pharmaceutical Sciences, Cheeloo College of Medicine, Shandong University, Jinan, Shandong China; 2 State Key Laboratory of Discovery and Utilization of Functional Components in Traditional Chinese Medicine, Cheeloo College of Medicine, Shandong University, Jinan, Shandong China; 3 NMPA Key Laboratory for Technology Research and Evaluation of Drug Products, School of Pharmaceutical Sciences, Cheeloo College of Medicine, Shandong University, Jinan, Shandong China; 4 Department of Pharmacology, School of Pharmaceutical Sciences, Cheeloo College of Medicine, Shandong University, Jinan, Shandong China; 5 Department of Pharmacy, Shandong Provincial Key Medical and Health Discipline of Clinical Pharmacy, Shandong Provincial Third Hospital, Shandong University, Jinan, Shandong China; 6 Department of Hematology, Heze Municipal Hospital, Heze, Shandong China; 7 Department of Pharmacy, Shandong Provincial Hospital Affiliated to Shandong First Medical University, Jinan, Shandong China; 8 Department of Hematology, Qilu Hospital, Cheeloo College of Medicine, Shandong University, Jinan, Shandong China; 9 School of Stomatology, Shandong First Medical University & Shandong Academy of Medical Sciences, Jinan, Shandong China

**Keywords:** Ubiquitylation, Chronic myeloid leukaemia

## Abstract

BCR-ABL1, derived from structural chromosome rearrangements, is the driver mutation in chronic myeloid leukemia (CML). Targeting BCR-ABL1 for degradation is an ideal therapeutic strategy for CML, however, the regulatory mechanisms controlling BCR-ABL1 expression in CML remained unclear. Here, we identified PELI1 as a key regulator for maintaining BCR-ABL1 in CML. BCR-ABL1 upregulates PELI1 via the STAT5/FOXP3 pathway, and the increased PELI1 then interacts with and protects BCR-ABL1 from degradation in CML cells. Concurrently, PELI1 functions as a downstream effector to promote CML cell proliferation. Notably, genetic or pharmacological inhibition of PELI1 effectively suppresses the proliferation of both tyrosine kinase inhibitors (TKIs)-sensitive and TKI-resistant CML cells, as well as Leukemia stem cells (LSCs), which consequently ameliorates the disease burden and progression of CML. Collectively, our findings demonstrated that targeting PELI1 is a promising therapeutic strategy for CML that can overcome TKI resistance and eliminates LSCs.

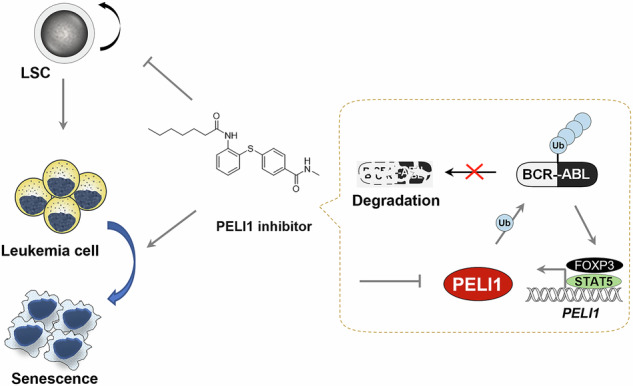

## Introduction

Abnormal gene fusions have been acknowledged as driver mutations in neoplasia, providing crucial insights into the mechanisms of disease involved in tumor formation [1]. *BCR-ABL1*, originating from the Philadelphia chromosome’s a translocation occurs between chromosomes 9 and 22, is the driver mutation in chronic myeloid leukemia (CML) [2, 3]. Tyrosine kinase inhibitors (TKIs) that bind to the ATP binding site of the ABL1 domain have significantly enhanced the prognosis for CML patients, yet the treatment outcomes in advanced CML patients remain poor [4]. Acquired resistance to TKIs and residual LSCs account for the disease relapse and progression [5–7].

BCR-ABL1 mutations lead to TKI resistance in patients with CML, prompting the development of TKIs from Imatinib (IM) to the third-generation Ponatinib (PO) [8, 9]. Given the driving role of BCR-ABL1 in CML development, targeting BCR-ABL1 degradation is an ideal strategy for achieving a functional cure. A previous study had highlighted the potential benefits of degrading BCR-ABL1 for CML that overcomes TKIs resistance, in which the degradation of BCR-ABL1 was accomplished through targeted protein degradation by Proteolysis-targeting chimera (PROTAC) [10]. Therefore, a more detailed understanding of BCR-ABL1 maintenance and its downstream effectors and signaling will provide vital clues for new therapies in CML.

Ubiquitination is a posttranslational modification involving covalent conjugation of ubiquitin (Ub) to lysine residues of target proteins, which is sequentially implemented by E1 activating enzymes, E2 conjugating enzymes, and E3 ligating enzymes [11]. Ubiquitination modification has been implied as potential drug target owing to its functional roles in the progression of leukemia and regulation of LSCs [12–16]. Pellino-1 (PELI1), an E3 ubiquitin ligase identified in recent years, has been documented to play roles in the onset and progression of autoimmunity, inflammatory disorders, infection, and cancer [17, 18]. This is mediated by targeting substrates for proteasomal degradation or protein stability via ubiquitination [3, 19, 20]. However, the role of PELI1 in hematologic malignancies remains unclear. This study revealed that PELI1 is an important regulator of the persistent BCR-ABL1 in CML. Targeting PELI1 inhibition was sufficient to reduce the growth of CML cells and ameliorate the disease burden and progression of CML that is not rely on the clinic BCR-ABL1 mutations.

## Materials and methods

### Animals and clinical samples

All mice were raised on the C57BL/6 genetic background. *PELI1* conditional knockout mice (*Peli1*^CKO^) were generated via the recombineering method (Supplementary Fig. 5A, see Supplemental Information for full details). Vav-cre mice were purchased from the Jackson Laboratory (Bar Harbor, USA). C57BL/6 and NOD-Prkdc^scid^IL2rg^tm1^/Cavens mice were obtained from Charles River Laboratories (Beijing, China). The experiment utilized groups that were matched by age and sex. All experimental protocols involving animals were conducted in strict accordance with the NIH Guide for the Care and Use of Laboratory Animals and were approved by the Institutional Animal Ethics Committee of Shandong University (#20021).

Bone marrow (BM) and peripheral blood (PB) samples from healthy donors and CML patients were collected at the Heze Municipal Hospital and Qilu Hospital of Shandong University. The use of human specimens was approved by the Ethics Committee of Heze Municipal Hospital and Qilu Hospital of Shandong University (KYLL-2023(ZM)-576). Informed consent was acquired following the Declaration of Helsinki. The information for the human samples was shown in Supplementary Table 2.

### Cellular thermal shift assay

The Cellular Thermal Shift Assay (CETSA) was executed according to the procedure [21]. In brief, K562 cells were exposed to either 10 μM BZ61 or DMSO for 4 h. Each sample was suspended in PBS supplemented with protease inhibitors, and then the samples were freeze-thawed five times and centrifuged at 20,000 g for 20 min at 4 °C. The supernatant was distributed into eight-tubes and thermally heated for 5 minutes at a certain temperature gradient. The supernatant standing at room temperature for 5 min and centrifuged again, after which the supernatant was denatured and subjected to western blot analysis. To verify the bindings of BZ61 and PELI1 in vitro, K562 cells lysate was incubated with different dose of BZ61 at 48°C for 5 min, and the soluble PELI1 was analyzed by Western blotting.

### Colony-forming cells assay

GFP^+^ LSK were plated (1 × 10^3^) in methylcellulose medium (StemCell^TM^, #03534, Canada) and colony number was counted 10 days later. For serial replating, GFP^+^ LSK cells from colonies in the well were diluted 20-fold and then replated (1 × 10^3^) in fresh methylcellulose; colony number was counted again after 10 days. Human CD34^+^ cells (5 × 10^3^) were seeded in methylcellulose medium (StemCell^TM^, #04434, Canada), with colony counts taken on day 14. For the serial replating, the same approach was applied as the mouse colony-forming cells assay.

### Surface plasmon resonance analysis

To kinetically analyze the affinity of PELI1 with BZ61, SPR analysis was carried out as described [22]. using Biacore^TM^ T200 (GE Healthcare, Pittsburgh, USA). Briefly, following the manufacturer’s instructions, PELI1 proteins at 20 μg/mL in 10 mM sodium acetate (pH = 5) were fixed onto a CM5 sensor chip using amine coupling chemistry (Cytiva, BR100012, USA), then flowed through with gradient dilution of BZ61 compound (0–37 μM). The dissociation constant (KD) was evaluated by the built-in evaluation software and graphed with GraphPad Prism 8.0.

### Chromatin immunoprecipitation (ChIP)

ChIP assays were conducted with SimpleChIP^®^-ChIP Assay Kits (Cell Signaling Technology, #9003, USA) following the supplier’s guidelines. Briefly, HEK293T cells were transfected with *STAT5B* (HA-tagged) or FOXP3 (FLAG-tagged) and PELI1 promoter plasmids for 24 h, then the cells were treated with 1% formaldehyde for 10 min at room temperature and then subjected to immunoprecipitation using IgG antibody (2 μg, Proteintech, 30000-0-AP, China) and STAT5 antibody (2 μg, Santa Cruz Biotechnology, sc-835, USA) overnight at 4 °C with rotation, subsequently, ChIP-Grade Protein G Magnetic Beads are added and incubate for 2 h at 4 °C with rotation, then the magnetic beads was washed with low salt and high salt buffer for 3 times at 4 °C with rotation alternately and then carefully remove the supernatant by placing the tubes in a magnetic separation rack. The bound DNA quantity was measured through real-time PCR with the PELI1 primers that were provided in Supplementary Table 3.

### CML patient-derived xenografts

CD34^+^ cells were extracted from the BM and PB of CML patients by a CD34^+^ Selection Kit (StemCell^TM^, #17856, Canada) and administered by tail vein injection into irradiated (100 cGy) 8 weeks of age male NOD-Prkdc^scid^IL2rg^tm1^/Cavens mice. After 4 weeks of acclimation, the mice were treated with either vehicle or BZ61 (25 mg/kg, administered intraperitoneally every other day) for 2 weeks. Following the collection and staining of cells, flow cytometry was employed to assess the proportion of leukemic cells (hCD45^+^, hCD45^+^CD34^+^, hCD45^+^CD11b^+^) in PB and BM. The antibodies employed for this experiment are presented in Supplementary Table 4.

### In vitro ubiquitination assay

An in vitro ubiquitination assay was performed according to the protocol provided with the E3 Ligase Auto-Ubiquitylation Assay kit (Abcam, ab139469, Britain). Briefly, 18 μg of FLAG-tagged BCR-ABL1 plasmid was introduced into HEK293T cells for 48 h, followed by immunoprecipitation of BCR-ABL1 using an anti-FLAG antibody and 40 μL of Protein A/G PLUS-Agarose (Santa Cruz Biotechnology, sc-2003, USA). The resulting construct was transformed into Escherichia coli BL21 cells for recombinant protein expression. PELI1 protein was expressed and purified following a standard prokaryotic expression protocol. For the ubiquitination reaction, 10 μg of FLAG-BCR-ABL1 and purified PELI1 were incubated with E1 (50 nM), E2 (UbcH5a, 0.3 mM), ubiquitin (10 μM), and ATP (2 mM) as recommended by the manufacturer. The reaction proceeded at 37 °C for 1.5 h and was terminated by adding 1× SDS-PAGE loading buffer. The samples were then subjected to immunoblot analysis using an anti-ubiquitin antibody.

### Statistics

All statistical analyses were conducted using Student’s t-tests, and the data are presented as mean ± SD. Survival data were displayed using Kaplan-Meier curves. The *P* values < 0.05 were considered significant.

## Results

### PELI1 interacted with and protected BCR-ABL1 from degradation via K63-linked polyubiquitination

**Fig. 1:**
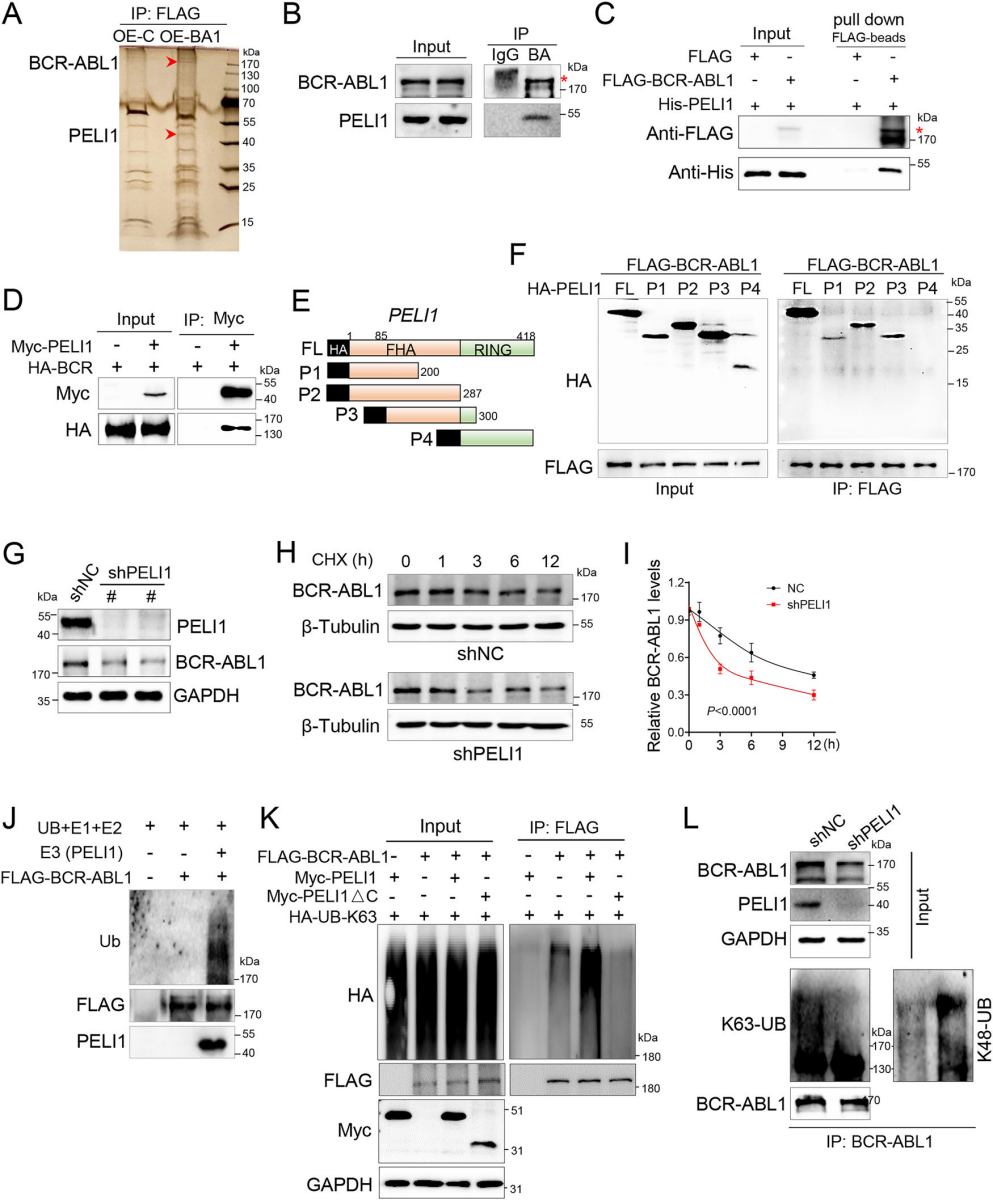
PELI1 protected BCR-ABL1 from degradation via K63-linked polyubiquitination. **A** Proteomic study of BCR-ABL1 interacting proteins in K562 cells transduced with retroviral constructs encoding FLAG-BCR-ABL1 (OE-BA1) or empty vector (OE-C). Proteins immunoprecipitated using anti-FLAG were resolved by SDS-PAGE and visualized by silver staining followed by mass spectrometry analysis. **B** Co-IP analysis of endogenous BCR-ABL1 binding to PELI1 in K562 cells with anti-IgG and anti-cABL1 (BA) antibodies respectively. **C** Pull-down assays with anti-FLAG agarose beads from the protein mixtures of BCR-ABL1 (FLAG-tagged, 10 μg) or FLAG and PELI1 (His-tagged, 10 μg). **D** Co-IP analysis of PELI1 binding to BCR segment of BCR-ABL1 using anti-Myc antibody in HEK293T cells co-transfected with BCR (HA-tagged) and PELI1 (Myc-tagged) plasmids. **E** Mapping of full-length and truncated human *PELI1*. (**F**) Co-IP analysis of BCR-ABL1 binding to PELI1 truncations with HA antibody in HEK293T cells co-transfected with BCR-ABL1 (FLAG-tagged) and PELI1 truncations as in E (HA-tagged). **G** Immunoblotting analysis of BCR-ABL1 in K562 cells transduced with retroviruses encoding indicated shRNAs. shNC represents a non-targeting shRNA. GAPDH was used as the loading control. (**H-I**) Immunoblotting analysis of BCR-ABL1 in K562 cells with indicated shRNAs upon cycloheximide (CHX, 10 μM) treatment. BCR-ABL1 levels were normalized to the change of GAPDH. shNC represents a non-targeting shRNA. Data were presented as mean ± SD from three independent experiments. *P* value was determined by two-way ANOVA. **J** In vitro ubiquitination assays with recombinant PELI1(10 μg) and BCR-ABL1(10 μg) proteins in the presence of ubiquitin (Ub), E1 and E2 (UbcH5A). **K** Immunoblotting analysis of the K63-linked polyubiquitination of BCR-ABL1 immunoprecipitated by anti-FLAG antibody from HEK293T cells transfected with BCR-ABL1 (FLAG-tagged), PELI1 or PELI1ΔC (RING-deletion mutant, Myc-tagged) and K63-only ubiquitin (HA-tagged) plasmids. **L** Immunoblotting analysis of ubiquitination of endogenous BCR-ABL1 immunoprecipitated by anti-cABL1 antibody from K562 cells with *PELI1* shRNAs, using K63-linkage and K48-linkage polyubiquitin antibodies. shNC represents a non-targeting shRNA. GAPDH was used as the loading control.

To examine the basic mechanisms associated with the expression of the BCR-ABL1 protein, we performed immunoprecipitations with anti-FLAG antibody in K562 cells ectopically expressing FLAG-tagged BCR-ABL1 (Fig. 1A). Multiple previously reported BCR-ABL1-interactors were detected by LC-MS, such as GRB2, USP10 and β-catenin [23–25]. suggesting the high reliability of the IP/MS data (Fig. 1A and Supplementary Table 1). Among the potential interactors, PELI1 was attractive due to its role in protein stability via ubiquitination, with elevated expression levels in BM cells obtained from CML patients in comparison to healthy donors (GSE47927) (Supplementary Fig. 1A).

The interaction of BCR-ABL1 and PELI1 was confirmed by the co-IP assays in HEK293T cells ectopically expressing PELI1 and BCR-ABL1, and also their endogenous binding in K562 cells (Fig. 1B and Supplementary Fig. 1B). Furthermore, the in vitro FLAG-beads pull-down assays also showed that PELI1 were specifically retained in the presence of BCR-ABL1, indicating their directly physical interaction (Fig. 1C). To identify the basis of the interaction of PELI1 and BCR-ABL1, co-IP experiments were performed in HEK293T cells transfected with HA-tagged BCR or ABL1 domains together with Myc-tagged PELI1 respectively and found that BCR but not ABL1 segment of BCR-ABL1 was pulled down by PELI1 (Fig. 1D and Supplementary Fig. 1C). To map the domains of PELI1 binding with BCR-ABL1, we also generated a battery of truncation in PELI1 (Fig. 1E), by which we demonstrated FHA domain (85-200 aa) of PELI1 is responsible for its binding with BCR-ABL1 (Fig. 1F).

Since PELI1 acts as an E3 ubiquitin ligase, we hypothesized that it regulates BCR-ABL1 protein stability through ubiquitination. As expected, BCR-ABL1 protein levels were markedly reduced in K562 cells transduced with retrovirus encoding PELI1 shRNAs, while its mRNA levels remained unchanged (Fig. 1G and Supplementary Fig. 1D). Knockdown of *PELI1* accelerated BCR-ABL1 degradation upon cycloheximide (CHX) treatment (Fig. 1H, I). This degradation was rescued by the proteasome inhibitor MG132 (Supplementary Fig. 1E). In contrast, overexpression of PELI1 in K562 cells significantly increased BCR-ABL1 protein levels with modest increase of its mRNA levels (Supplementary Fig. 1F, G). PELI1 overexpression also conferred resistance to CHX-induced BCR-ABL1 degradation (Supplementary Fig. 1H, I). We therefore performed in vitro ubiquitination assays and found that PELI1, in the presence of E1 activating and E2 conjugating enzymes, successfully assembled polyubiquitin chains on BCR-ABL1 (Fig. 1J), confirming that PELI1 directly ubiquitinates BCR-ABL1.

To characterize the type of ubiquitin linkage mediated by PELI1, co-IP assays were performed in HEK293T cells transfected with plasmids encoding K63-only or K48-only ubiquitin, together with PELI1 and BCR-ABL1. Overexpression of full-length PELI1, but not its RING-deletion mutant (Peli1ΔC, which lacks E3 ligase activity) [26]. significantly enhanced K63-linked polyubiquitination of BCR-ABL1 (Fig. 1K). In contrast, no increase in ubiquitination was observed with K48-linked ubiquitin, indicating that PELI1 specifically promotes K63-linked polyubiquitin chain formation on BCR-ABL1 (Supplementary Fig. 1J). Consistent with this, PELI1 knockdown led to the reduced K63-linked ubiquitination but increased K48-linked ubiquitination of BCR-ABL1 in K562 cells (Fig. 1L). Together, these results demonstrate that PELI1 stabilizes BCR-ABL1 via K63-linked polyubiquitination in CML cells.

### BCR-ABL1 upregulated PELI1 expression through the STAT5-FOXP3 signaling in CML

**Fig. 2:**
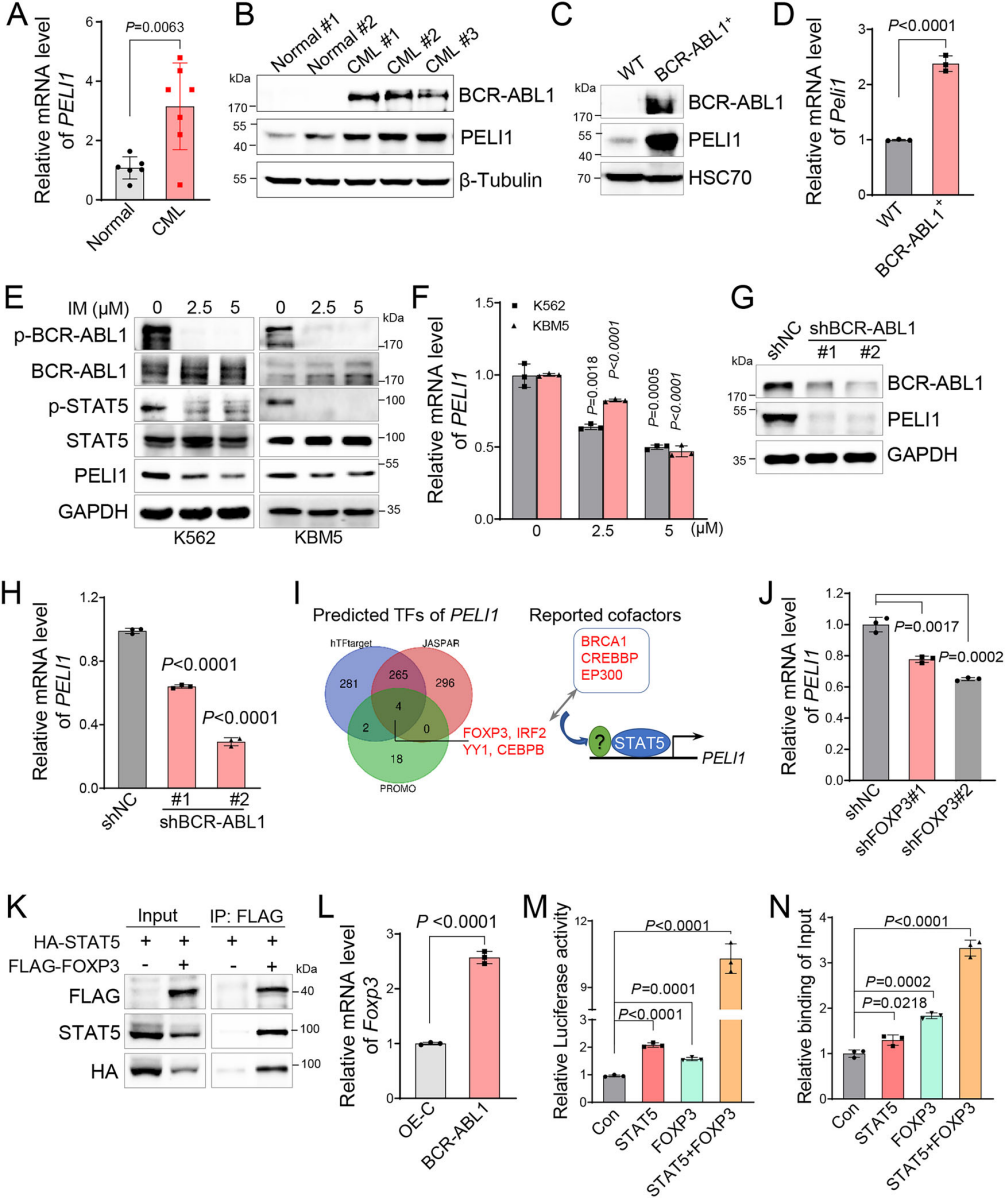
BCR-ABL1 regulated PELI1 expression via STAT5/FOXP3 transcriptional activation in CML cells. **A** Quantitative PCR analysis of *PELI1* mRNA levels in CD34^+^ BM or PB cells from CML patients (CML#1-7 in Supplementary Table 2) and healthy donors (Normal#1-6 in Supplementary Table 2). Each dot represents one human sample. Data were presented as mean ± SD. **B** Immunoblotting analysis of PELI1 in the bone marrow mononuclear cells from 3 CML patients (CML#8-10 in Supplementary Table 2) and 2 healthy donors (Normal#7-8 in Supplementary Table 2). β-Tubulin was used as the loading control. **C** Immunoblotting analysis of BCR-ABL1 and PELI1 in BM mononuclear cells from BCR-ABL1-driven CML mice and wild-type controls. HSC70 was used as the loading control. **D** Quantitative PCR analysis of *Peli1* mRNA levels in cells as in C. Data were presented as mean ± SD from three independent experiments. **E** Immunoblotting analysis of indicated proteins in K562 and KBM5 cells with Imatinib treatment for 10 h. GAPDH was used as the loading control. **F** Quantitative PCR analysis of *PELI1* mRNA levels in cells as in E. Data were presented as mean ± SD from three independent experiments. **G** Immunoblotting analysis of indicated proteins in K562 cells transduced with retroviruses encoding indicated shRNAs. shNC represents a non-targeting shRNA. GAPDH was used as the loading control. #1/#2 represent independent shRNAs targeting *BCR-ABL1*. **H** Quantification of *PELI1* mRNA levels in cells as in (**G**). Data were presented as mean ± SD from three independent experiments. **I** Schematic of the screening of the co-factors of STAT5 for the regulation of PELI1. Several candidates for cofactors of STAT5 were identified from previous publications and predictions with databases including JASPAR (https://jaspar.genereg.net/), PROMO (http://alggen.lsi.upc.es/cgi-bin/promo_v3), and hTFtarget (http://bioinfo.life.hust.edu.cn/hTFtarget/) databases. **J** Quantitative PCR analysis of *PELI1* mRNA levels in K562 cells transduced with retroviruses encoding *FOXP3* shRNAs. shNC represents a non-targeting shRNA. Data were presented as mean ± SD from three independent experiments. **K** Co-IP analysis of FOXP3 binding to STAT5 in HEK293T cells co-transfected with FOXP3 (FLAG-tagged) and STAT5 (HA-tagged) plasmids. **L** Quantitative PCR analysis of *Foxp3* mRNA levels in Ba/F3 cells transduced with retroviral constructs encoding BCR-ABL1 or empty vector (OE-C). Data were presented as mean ± SD from three independent experiments. **M** Luciferase reporter assays of *PELI1* promoter in HEK293T cells co-transfected with indicated genes and *PELI1* promoter luciferase reporter. Data were presented as mean ± SD from three independent experiments. **N** Chromatin immunoprecipitation analysis of the binding activity of STAT5 on *PELI1* promoter in HEK293T cells transfected with the indicated expression constructs. Data were presented as mean ± SD from three independent experiments.

In line with the upregulation of PELI1 in CML (Supplementary Fig. 1A), our independent analysis using BM cells from 7 CML patients also revealed that PELI1 was highly expressed in CML compared with that of healthy donors (Fig. 2A, B). Additionally, we employed a recognized BM transplantation model featuring ectopic *BCR-ABL1* expression (Supplementary Fig. 2A), which recapitulates the disease mechanisms observed in human chronic myeloid leukemia patients [27–29]. Compared to the wild-type controls, PELI1 expression was significantly upregulated in BM mononuclear cells of BCR-ABL1-driven CML mice (Fig. 2C, D). Similar observations were made in Ba/F3 cells that overexpress BCR-ABL1(Supplementary Fig. 2B, C).

We next examined the PELI1 expression in BCR-ABL1-positive CML cell lines and found that Imatinib inhibited PELI1 expression as well as the phosphorylation of STAT5 (Fig. 2E, F). Genetic ablation of *BCR-ABL1* similarly reduced PELI1 expression in K562 cells (Fig. 2G, H). Since STAT5 functions as a key downstream mediator of BCR-ABL1 signaling, pharmacological inhibition of STAT5 with inhibitor in K562 cells resulted in a marked suppression of PELI1 level (Supplementary Fig. 2D, E).

Putative STAT5-binding sites (TTC*NNN*GAA) were identified in the promotor of *PELI1*. This finding was further supported by the ENCODE database, which revealed STAT5-binding peaks close to the transcriptional start site of *PELI1* in K562 cells (Supplementary Fig. 2F, G). However, STAT5 alone did not substantially increase *PELI1* promoter-driven luciferase activity in HEK293T cells (Supplementary Fig. 2H), suggesting that STAT5 is insufficient for the upregulation of PELI1 induced by BCR-ABL1 in CML cells.

Several candidates for cofactors of STAT5 were identified from previous publications and predictions with databases (Fig. 2I). Interestingly, PELI1 expression in K562 cells was selectively reduced only upon *FOXP3* or *IRF2* depletion (Fig. 2J and Supplementary Fig. 2I, J). Co-IP assays demonstrated that STAT5 interacts with FOXP3 but not IRF2 (Fig. 2K and data not shown). Indeed, *Foxp3* was dramatically up-regulated by BCR-ABL1 in Ba/F3 cells (Fig. 2L), while BCR-ABL1 inhibition downregulated *FOXP3* expression in K562 cells (Supplementary Fig. 2K, L).

Compared to the modest increase of luciferase activity observed in HEK293T cells expressing FOXP3 or STAT5 alone, co-expression of both proteins significantly enhanced the luciferase activity driven by *PELI1* promoter (Fig. 2M). This synergistic effect was further supported by the ChIP assays, which revealed that FOXP3 co-expression strongly promoted STAT5 recruitment to the PELI1 promoter compared to STAT5 expression alone (Fig. 2N). These results suggest that BCR-ABL1 regulates PELI1 expression through the STAT5-FOXP3 signaling axis.

### Silencing PELI1 attenuated the CML cells proliferation independent of TKI-resistant mutations of BCR-ABL1

**Fig. 3:**
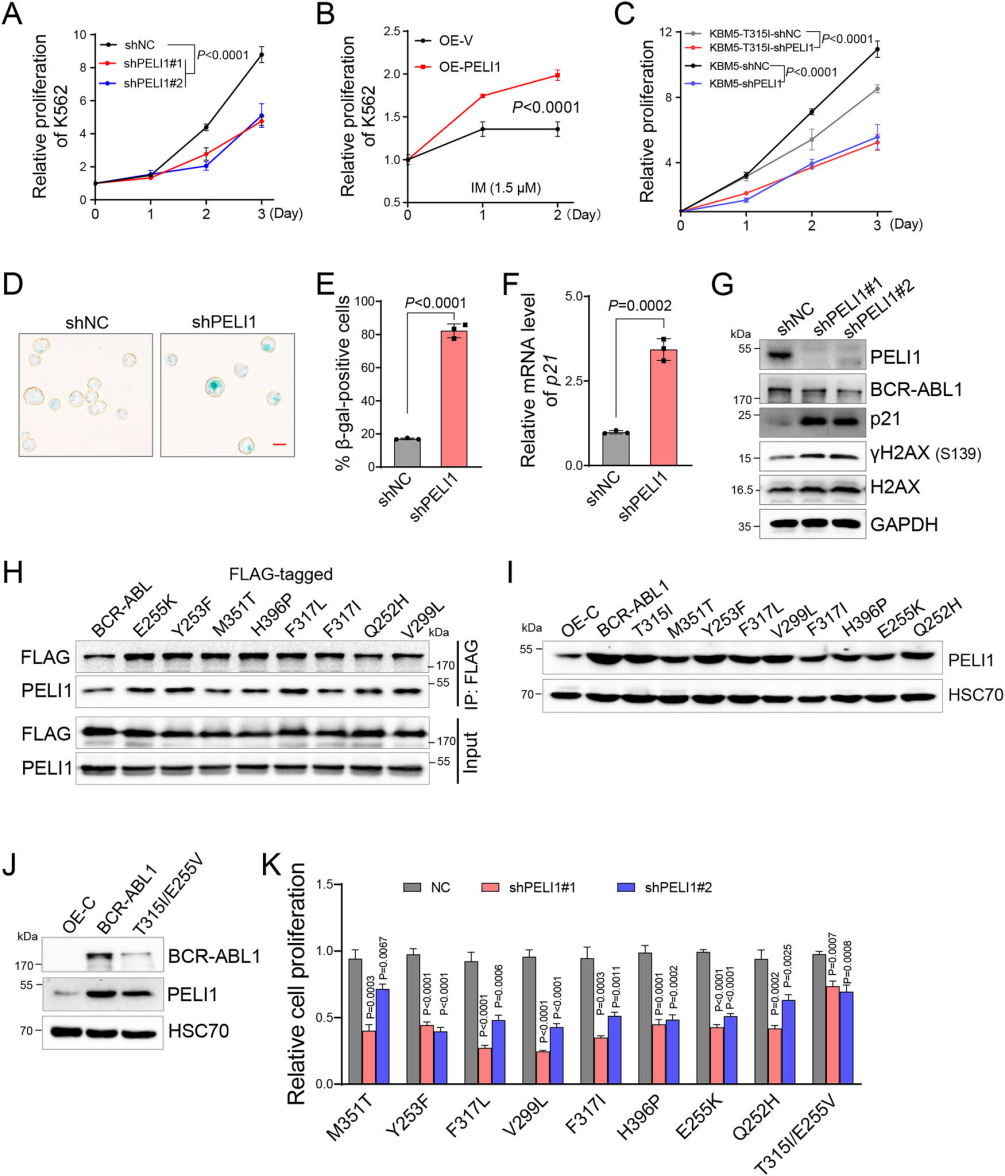
Silencing PELI1 inhibited the proliferation of CML cells. **A** Statistical analysis of cell proliferation in K562 cells with indicated shRNAs. shNC represents a non-targeting shRNA. Data were obtained from three independent experiments. *P* value was determined by two-way ANOVA. **B** Statistical analysis of cell proliferation in K562 cells transduced with retroviral constructs encoding BCR-ABL1 or empty vector (OE-C) upon the treatment of Imatinib (IM, 1.5 μM). Data were obtained from three independent experiments. *P* value was determined by two-way ANOVA. **C** Statistical analysis of cell proliferation in KBM5 and KBM5-T315I cells with indicated shRNAs. shNC represents a non-targeting shRNA. Data were obtained from three independent experiments. *P* value was determined by two-way ANOVA. **D**, **E** SA-β-gal staining in K562 cells transduced with indicated shRNAs. Quantification of SA-β-gal staining positive cells is shown as mean ± SD from three independent experiments. shNC represents a non-targeting shRNA. **F** Quantitative PCR analysis of *p21* mRNA levels in K562 cells with *PELI1* shRNA. shNC represents a non-targeting shRNA. Data were presented as mean ± SD from three independent experiments. **G** Immunoblotting analysis of indicated proteins in K562 cells with *PELI1* shRNA. shNC represents a non-targeting shRNA. GAPDH was used as the loading control. **H** Co-IP analysis of PELI1 binding to BCR-ABL1 using anti-FLAG antibody, in HEK293T cells co-transfected with Flag-BCR-ABL1 (mutants) and HA-PELI1 plasmids. **I**, **J** Immunoblotting analysis of Peli1 in Ba/F3 cells transduced with retroviral constructs encoding indicated BCR-ABL1 mutants or empty vector (OE-C). HSC70 was used as the loading control. **K** Statistical analysis of cell proliferation in Ba/F3 cells that stably expressed BCR-ABL1 mutants with *PELI1* shRNAs. shNC represents a non-targeting shRNA. Data were obtained from three independent experiments.

Considering the correlation between PELI1 and BCR-ABL1, we next examined whether PELI1 is involved in BCR-ABL1-deriven CML. *PELI1* knockdown substantially inhibited the proliferation of K562 cells, while its overexpression significantly attenuated the effect of IM on K562 cells (Fig. 3A, B). Interestingly, *PELI1* knockdown also led to the proliferative inhibition of KBM5 cells harboring BCR-ABL1 and BCR-ABL1^T315I^ mutants, respectively (Fig. 3C). To elucidate the PELI1’s regulatory mechanism in CML cells proliferation, we first assessed the cell cycle progression and apoptotic indices of K562 cells but found undetectable changes after PELI1 knockdown (data not shown). In contrast, senescence-associated β-galactosidase assays showed significant elevation of SA-β-gal-positive cells upon *PELI1* knockdown, indicating induction of cellular senescence (Fig. 3D, E). In line with this finding, p21 expression (regarded as a biomarker of senescence) was also upregulated in K562 cells with *PELI1* knockdown (Fig. 3F). Moreover, western blotting analysis showed that *PELI1* knockdown led to an increase of p21 protein levels, as well as the accumulation of phosphorylated H2AX (γH2AX) (Fig. 3G). Indeed, *BCR-ABL1* knockdown and IM treatment also markedly increased the SA-β-gal-positive cells and level of γH2AX, phenocopying the effects of *PELI1* knockdown in K562 cells (Supplementary Fig. 3A-D).

BCR-ABL1^T315I^ is a prevalent resistance-associated variant, confers insensitivity to first- and second-generation TKIs and represents a persistent therapeutic challenge in CML management [30, 31]. Co-IP assays showed that T315I mutation and other identified BCR-ABL1 mutants failed to impair the protein-protein interaction between BCR-ABL1 and PELI1 (Fig. 3H and Supplementary Fig. 4A). Similar high expressions of PELI1 were also observed in Ba/F3 carrying these BCR-ABL1 mutants compared with the corresponding cells (Fig. 3I and Supplementary Fig. 4B). Even the E255V/T315I mutant that was recently identified to confer resistance to Ponatinib [32–35]. also upregulated the PELI1 expression in Ba/F3 cells (Fig. 3J and Supplementary Fig. 4C). Notably, *PELI1* knockdown significantly inhibited the Ba/F3 cells proliferation driven by these BCR-ABL1 mutants even the Po-resistant mutation (Fig. 3K). Our results indicate that PELI1 ablation inhibits the proliferative capacity of CML cells irrespective of BCR-ABL1 mutations.

### Loss of PELI1 ameliorated the disease progression and leukemic burden of CML in vivo

To clarify the role of PELI1 in hematopoiesis, we crossed *Peli1*^fl/fl^ mice with Vav-Cre transgenic mouse to generate a hematopoietic-specific knockout mouse model (Supplementary Fig. 5A, B, referred to as CKO mice). *Peli1* deletion did not affect the hematopoietic profile characterized by the comparable blood cell counts in young mice as WT mice (2 months of age). The older CKO mice (12 months of age) exhibited mild leukopenia coupled with minor anemia (Supplementary Fig. 5C), suggesting that PELI1 plays important roles in stressed hematopoiesis.

**Fig. 4:**
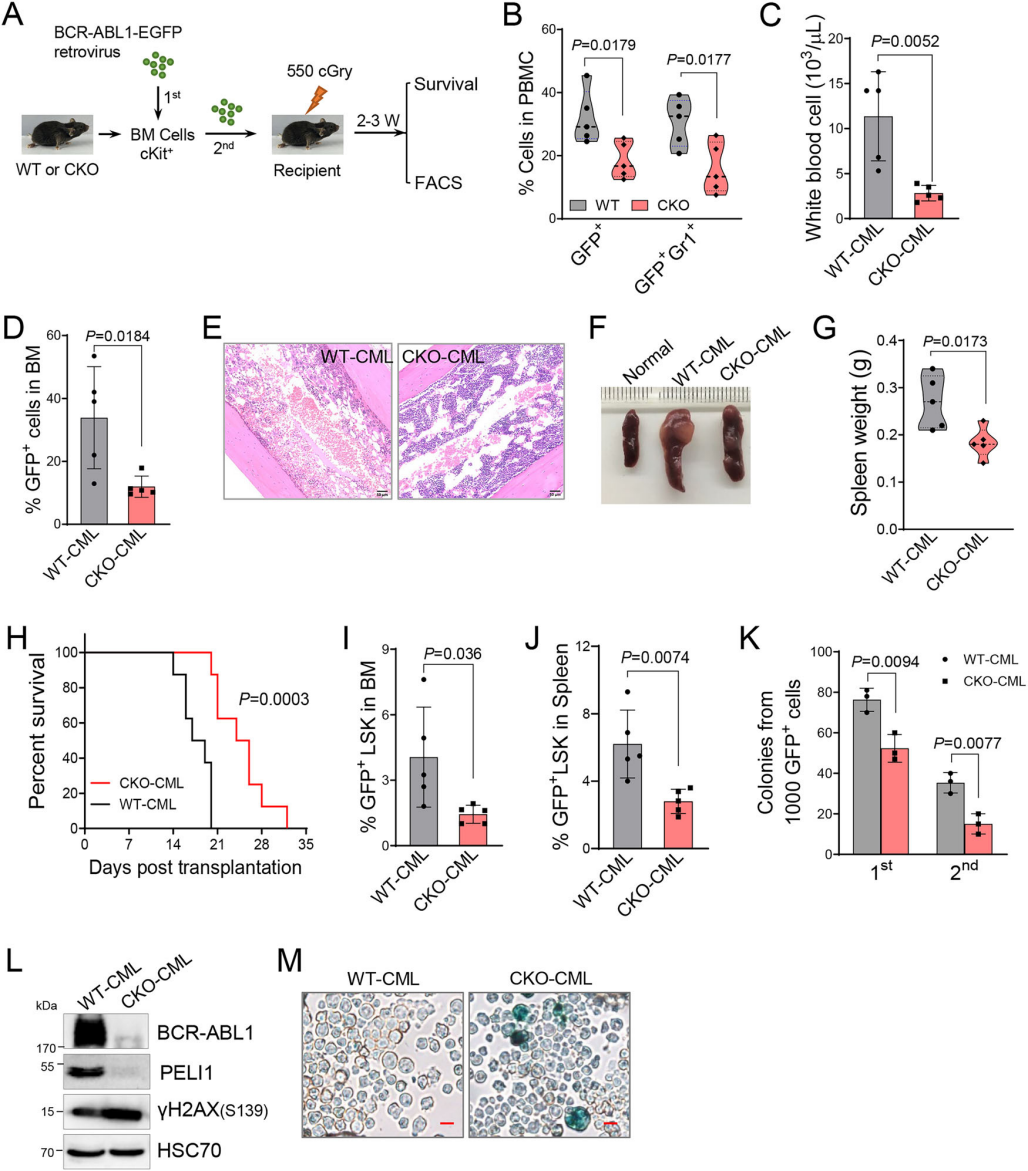
Loss of Peli1 ameliorated the progression of BCR-ABL1-driven CML. **A** Schematic representation of BCR-ABL1-driven CML mice model with wild type (WT) or *Peli1* conditional knockout (CKO) mice bone marrow cells. **B** Flow cytometry analysis of leukemia cells (GFP^+^ and GFP^+^Gr1^+^) in peripheral blood mononuclear cells (PBMN) from the mice as in A (*n* = 5). Each dot represents one mouse. Data were presented as mean ± SD. **C** Statistical analysis of white blood cells in peripheral blood from indicated mice as in A (WT-CML *N* = 7 and CKO-CML *N* = 5). Each dot represents one mouse. Data were presented as mean ± SD. **D** Flow cytometry analysis of leukemia cells (GFP^+^) in the bone marrow from indicated mice as in (**A**) (*N* = 5). Data were presented as mean ± SD. **E** Representative Haematoxylin and eosin (H&E) stains of bone marrow from indicated mice as in (**A**). **F** Representative spleen of indicated mice as in (**A**) was shown. **G** Statistical analysis of spleen weight from indicated mice as in (**A**) (*N* = 5). Each dot represents one mouse. Data were presented as mean ± SD. **H** Kaplan-Meier survival curves of indicated mice after bone marrow transplantation as in (**A**). *N* = 8 mice per group. *P* value was determined by Log-rank test. **I**, **J** Flow cytometry analysis of CML-LSCs (GFP^+^LSK^+^) in the BM and spleen from the mice as in (**A**) (*N* = 5). Each dot represents one mouse. Data were presented as mean ± SD. **K** Colony forming cell assays with GFP^+^LSK^+^ in bone marrow from indicated mice. Data were presented as mean ± SD from 3 mice of indicated genotype. **L** Immunoblotting analysis of indicated proteins in mononuclear bone marrow cells from indicated mice as in (**A**). HSC70 was used as the loading control. **M** Representative SA-β-gal staining in cells as in (**A**). Scale bars represent 20 μm.

To gain a deeper insight into the function of PELI1 in BCR-ABL1-driven CML pathogenesis, we used a CML mouse model by transducing *Peli1*-CKO or WT BM with BCR-ABL1 overexpression and transplanting them into sub-lethally irradiated recipient mice (Fig. 4A, termed CKO-CML and WT-CML). Homing analysis revealed that *Peli1* knockout did not impair the engraftments of LSC (GFP^+^LSK^+^, Supplementary Fig. 5D). As previously reported [36]. myeloid blast crisis predominated in CML peripheral blood (Supplementary Fig. 5E). CKO-CML mice exhibited significantly decreased peripheral leukemic burden (GFP^+^Gr1^+^) at day 14 post-transplantation (Fig. 4C), corresponding to diminished total leukocyte counts (Fig. 4B). Similar suppression of leukemic cells was observed in bone marrow from CKO-CML mice (Fig. 4D). Consequently, the impaired BM structure due to the abnormal proliferation of leukemic myeloid cells was also largely reversed by *Peli1* depletion (Fig. 4E). Moreover, loss of *Peli1* greatly ameliorated the CML cells invasion in both spleen and lung tissue (Fig. 4F, G and Supplementary Fig. 5F-H). Notably, *Peli1* deletion dramatically rescued BCR-ABL1-triggered mortality (Fig. 4H and Supplementary Fig. 5I).

CKO-CML mice also showed the diminished LSCs (GFP^+^LSK cells) in BM and spleen (Fig. 4I, J). This observation was further corroborated by the substantially reduced colony formation activity from CKO-CML mice ex vivo (Fig. 4K). However, both group mice showed comparable GFP^-^LSK cells in bone marrow, suggesting that PELI1 deficiency had no effect on normal HSCs (Supplementary Fig. 5J, K). Consistent with in vitro findings, CKO-CML mice also exhibited upregulation of γH2AX expression, concomitant with the increase of cellular senescence compared with that of WT-CML mice (Fig. 4L, M).

Similar inhibition of CML progression was also observed in BCR-ABL1^T315I^ and BCR-ABL1^T315I/E255V^-driven CML mice upon the *Peli1* deletion (Supplementary Figs. 6, 7), indicating that targeting PELI1 is sufficient to ameliorate the disease burden and progression of CML that is independent of TKI-resistant BCR-ABL1 mutations.

### Inhibition of PELI1 exhibited effective therapy for CML

**Fig. 5:**
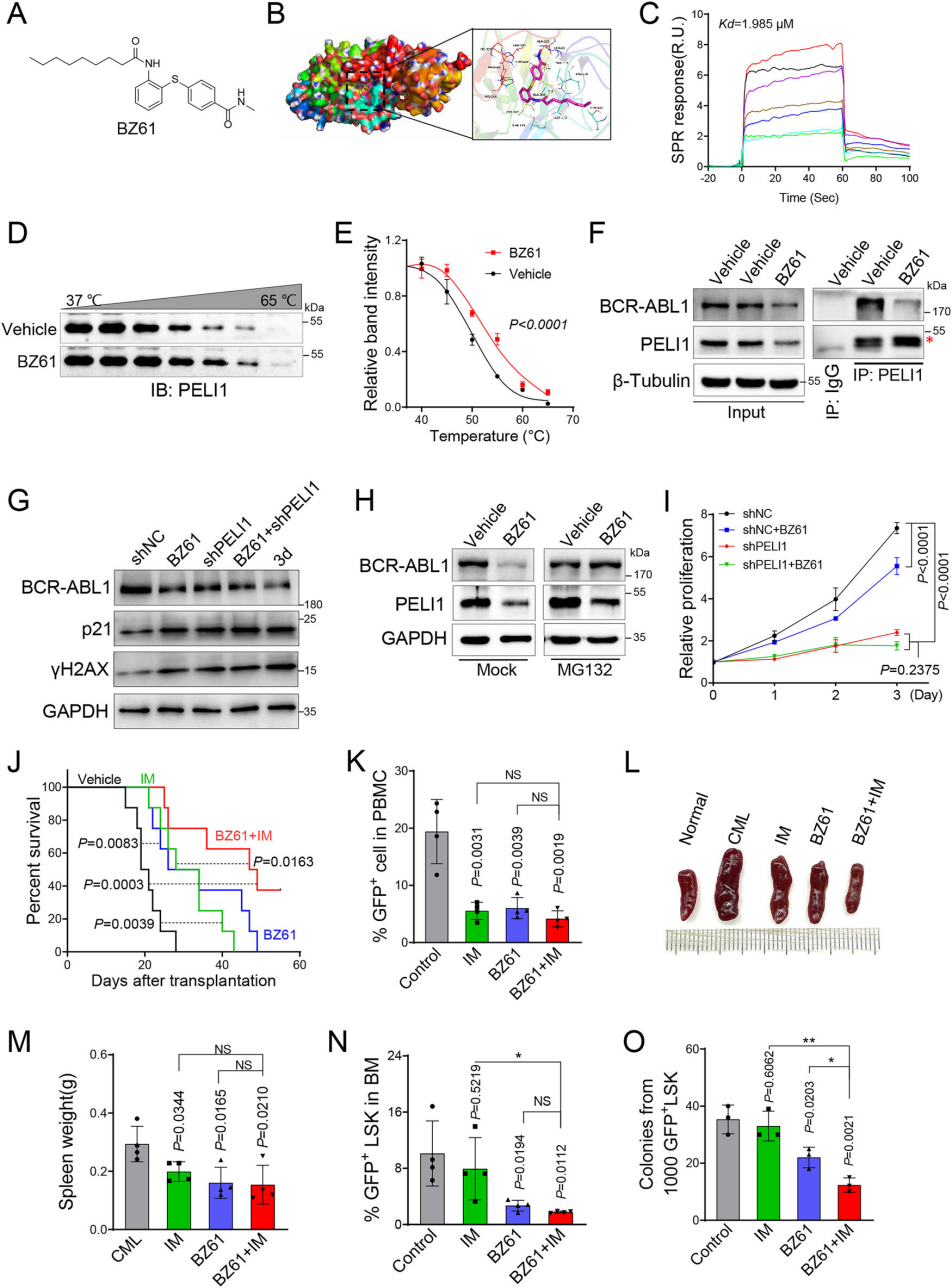
Pharmacological inhibition of PELI1 is sufficient to ameliorate the CML phenotypes driven by BCR-ABL1. **A** Chemical structure of BZ61. **B** 3D (left) and surface (right) view models show the binding pocket and binding of PELI1 and BZ61. **C** Surface Plasmon Resonance (SPR) analysis revealed the kinetic interaction of BZ61 and PELI1. The color curves represent concentrations of BZ61 (0–37 μM) from down to up. The dissociation constant (*Kd*) was calculated by the built-in evaluation software and indicated the affinity for PELI1 and BZ61. **D**, **E** Cellular thermal shift assays show the binding of PELI1 with BZ61 in K562 cells. K562 cells were treated with BZ61 (10 μM) or DMSO for 1 h, respectively. Cell lysate was collected and divided into 7 equal parts, followed by the incubation of indicated temperature for 5 min and Western blotting for the soluble proteins. Data were presented as mean ± SD from three independent experiments. *P* value was determined by two-way ANOVA. **F** Co-IP analysis of PELI1 binding to BCR-ABL1 in K562 cells with BZ61 (10 μM) for 18 h. **G** Immunoblotting analysis of the indicated proteins in K562 cells treated with BZ61 (10 μM), 3 d (10 μM) or *PELI1* shRNA for 10 h. GAPDH was used as the loading control. **H** Immunoblotting analysis of indicated proteins in K562 cells as in G except additional incubation of MG132 (10 μM) for 4 h. GAPDH was used as the loading control. **I** Statistical analysis of cell proliferation in K562 cells transduced with retrovirus encoding indicated shRNAs with the treatment of BZ61 (10 μM). shNC represents a non-targeting shRNA. Data was obtained from three independent experiments. *P* value was determined by two-way ANOVA. **J** Kaplan-Meier survival curves of CML mice after the treatment of BZ61 (25 mg/kg, administered intraperitoneally every other day), IM (100 mg/kg/day, i.g.) and their combination as in Supplementary Fig. 9 F. *N* = 8 mice for each group. *P* value was determined by Log-rank test. **K** Statistical analysis of leukemia cells (GFP^+^) in peripheral blood mononuclear cells from indicated mice as in J. Each dot represents one mouse. Data were presented as mean ± SD. NS indicates not significant (*P* > 0.05). (**L**) Representative spleen of indicated mice as in (**J**). **M** Statistical analysis of spleen weight from the mice as in L. Each dot represents one mouse. Data were presented as mean ± SD. NS indicates not significant (*P* > 0.05). **N** Statistical analysis of GFP^+^ LSK^+^ cells in bone marrow from indicated mice as in (**J**). Each dot represents one mouse. Data were presented as mean ± SD. *: *P* < 0.05; NS: not significant (*P* > 0.05). **O** Statistical analysis of colonies derived from indicated mice as in J. Each dot represents one mouse. Data were presented as mean ± SD. *: *P* < 0.05, **: *P* < 0.01; NS: not significant (*P* > 0.05).

To gain therapeutic understanding of PELI1 inhibition in the treatment of CML, we screened an internal library of 200 small molecules and identified BZ61 as a putative PELI1 inhibitor (Fig. 5A and Supplementary Fig. 8A). This selection was based on observed changes in the protein levels of SNAIL and SLUG, which are known substrates of PELI1-mediated K63-linked ubiquitination [37]. Computational docking showed that PELI1 adopts a compact conformation to bind with BZ61 at Asp-116, Thr-117, and Thr-250 (Fig. 5B). This binding was further supported by a high affinity for PELI1 and BZ61 (*Kd* = 1.985 μM) as indicated by SPR (Fig. 5C and Supplementary Fig. 8B). Cellular thermal shift assays also demonstrated that BZ61 binds to PELI1 in K562 cells (Fig. 5D, E) and in vitro (Supplementary Fig. 8C).

To further verify that BZ61 directly inhibits PELI1, we assessed its effects on the known substrates Snail and STAT3. In K562 cells, BZ61 treatment markedly reduced the expression of both Snail and STAT3 (Supplementary Fig. 8D). Co-IP assays in HEK293T cells transfected with K63 ubiquitin together with PELI1 and Snail or STAT3 plasmids demonstrated that BZ61 suppresses K63-linked ubiquitination of Snail and STAT3 (Supplementary Fig. 8E, F). Furthermore, in vitro ubiquitination assays confirmed that BZ61 disrupts the assembly of polyubiquitin chains on Snail or STAT3 by PELI1 in the presence of E1 activating and E2 conjugating enzymes (Supplementary Fig. 8G, H).

We then assessed the efficacy of BZ61 in CML cells. Proliferation assays revealed that BZ61 inhibited K562 cells in a dose-dependent manner, with an IC_50_ of 13.614 μM (Supplementary Fig. 9A). A concentration of 10 μM was selected for subsequent experiments. Co-IP assays showed that BZ61 markedly reduced the interaction between PELI1 and BCR-ABL1(Fig. 5F and Supplementary Fig. 9B). Consistent with the previously reported effects of the PELI1 inhibitor 3 d [38]. treatment with BZ61 also led to a pronounced decrease in BCR-ABL1 protein levels but not mRNA levels (Fig. 5G and Supplementary Fig. 9C), along with a marked increase in SA-β-gal-positive cells and elevated expression of γH2AX and p21 (Fig. 5G and Supplementary Fig. 9D, E). These effects coincided with downregulation of PELI1 expression in K562 cells. The reduction in BCR-ABL1 induced by BZ61 was rescued by the proteasome inhibitor MG132 (Fig. 5H). In line with the effects of PELI1 silencing on CML cell lines, BZ61 also inhibited proliferation of CML cells, while it did not show additional inhibitory effects in PELI1-silenced CML cells (Fig. 5I). These data indicated that BZ61 acted as a PELI1 inhibitor to suppress the proliferation of CML cells.

Next, the effect of BZ61 on CML progression was assessed using a BCR-ABL1-driven CML mouse model (Supplementary Fig. 9F). The administration of BZ61 to CML mice resulted in body weights similar to the control group, which implies minimal toxicity (data not shown). BZ61 treatment notably extended the lifespan of CML mice (Fig. 5J), reduced the leukemic burden in PB and BM (Fig. 5K and Supplementary Fig. 9G, H), as well as the decreased splenomegaly (Fig. 5L, M and Supplementary Fig. 9I, J). Although IM treatment decreased tumor burden in CML mice, it had limited effect on the LSCs (GFP^+^LSK cells) that were largely diminished in BZ61-treated CML mice (Fig. 5N and Supplementary Fig. 9K). This was further confirmed by the significantly reduced colony formation from BZ61-treated but not IM-treated CML mice (Fig. 5O). Notably, combined BZ61 and IM showed further therapeutic benefits for CML compared to current TKI treatment.

To test whether BZ61 could address BCR-ABL1 mutation-dependent drug resistance, we also performed BZ61 administration in a Po-resistant CML mice model driven by BCR-ABL1^T315I/E255V^ mutation. Strikingly, BZ61 exhibited similar effects in alleviating disease burden and progression in Po-resistant CML driven by BCR-ABL1^T315I/E255V^ mutation (Supplementary Fig. S10).

### Pharmacological inhibition of PELI1 reduced human CML burden and proliferation of CML-LSCs in vivo

**Fig. 6:**
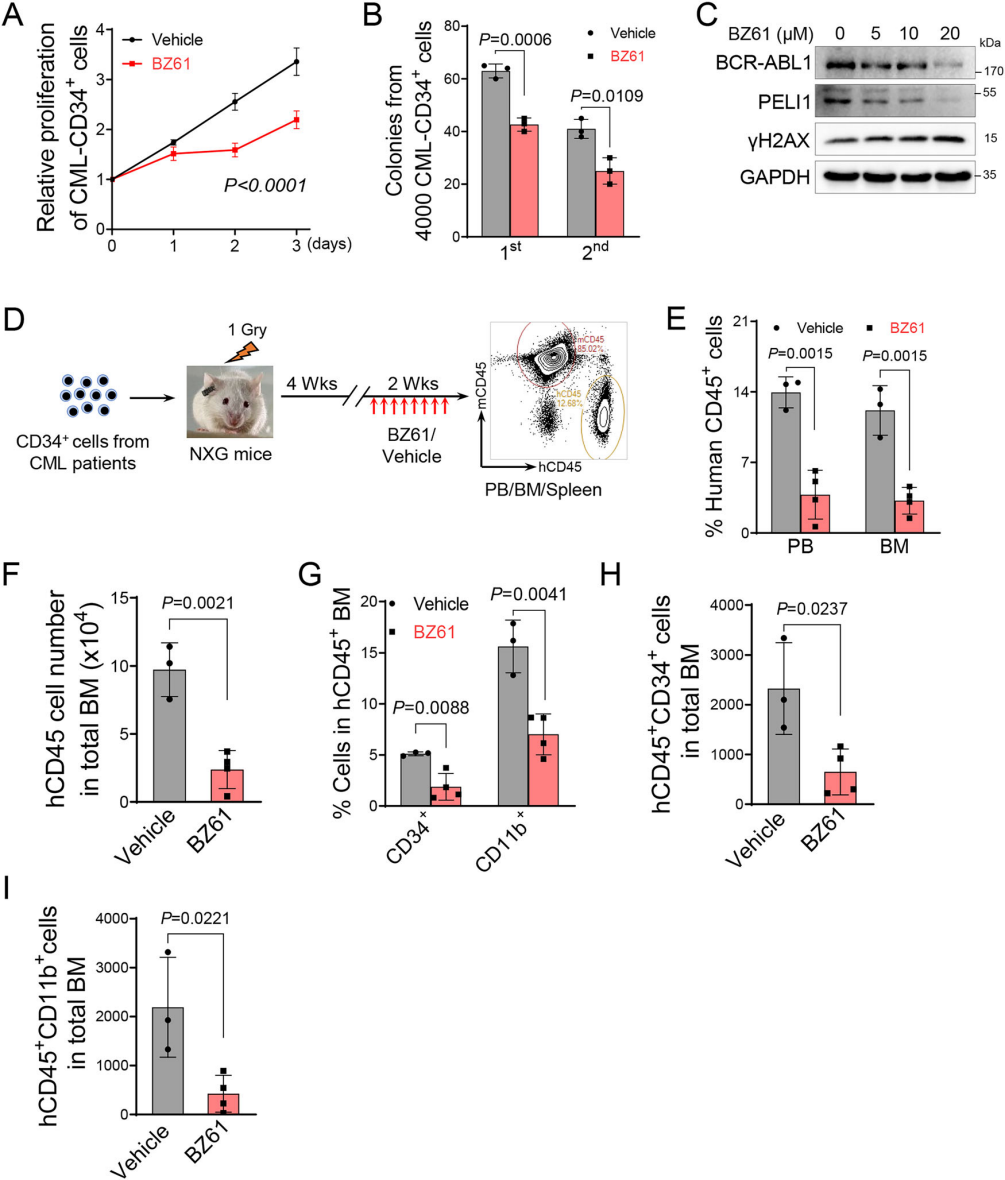
Pharmacological inhibition of PELI1 reduced human CML burden and proliferation of CML-LSCs. **A** Statistical analysis of cell proliferation in CD34^+^ bone marrow cells from CML patient (CML#11 in Supplementary Table 2) with the treatment of BZ61 (10 μM). Data were obtained from three independent experiments. *P* value was determined by two-way ANOVA. **B** Statistical analysis of colonies derived from the cells as in A. Data were presented as mean ± SD from three independent experiments. **C** Immunoblotting analysis of indicated proteins in CD34^+^ bone marrow cells from CML patient (CML#11 in Supplementary Table 2) with the treatment of BZ61 (10 μM). GAPDH was used as the loading control. **D** Experimental design for the CML xenograft model, in which 2.5 × 10^5^ CD34^+^ cells purified from peripheral blood of newly diagnosed patient with chronic CML (CML#12 in Supplementary Table 2) were injected into the tail veins of the sublethally irradiated NXG recipient mice (100 cGy). The mice were randomized for treatment (arrows) with vehicle or BZ61 (25 mg/kg, administered intraperitoneally every other day) for 2 weeks. Each dot represents one mouse. **E** Statistical analysis of human CD45^+^ in peripheral blood (PB) and bone marrow (BM) from indicated mice. Each dot represents one mouse. Data were presented as mean ± SD. **F** Statistical analysis of cell numbers of human CD45^+^ cells in bone marrow from the mice as in D. Each dot represents one mouse. Data were presented as mean ± SD. **G** Statistical analysis of CD34^+^ and CD11b^+^ cells derived from CML patient donors in the mice as in D. Each dot represents one mouse. Data were presented as mean ± SD. **H**, **I** Statistical analysis of cell numbers of CD34^+^ and CD11b^+^ cells in bone marrow from the mice as in D. Each dot represents one mouse. Data were presented as mean ± SD.

We next examined the therapeutic responses of primary human CML CD34^+^ cells to PELI1 inhibition. BZ61 significantly inhibited the expansion of the CD34^+^ cells obtained from CML patients in vitro (Fig. 6A), which was further verified by the sustained decrease in colony formation of these CML CD34^+^ cells when treated with BZ61 (Fig. 6B). Similar findings were also observed with *PELI1* silencing (Supplementary Fig. 11A, B). However, inhibiting PELI1 did not impact the proliferation of normal human CD34^+^ cells (Supplementary Fig. 11C). Furthermore, treatment with BZ61 also led to a pronounced decrease in BCR-ABL1 protein levels, along with elevated expression of γH2AX and p21 (Fig. 6C).

Xenotransplantation experiments were also carried out to assess the in vivo effects of BZ61 on CML, in which CD34^+^ cells from PB of a patient recently diagnosed with CML were transplanted into NOD-Prkdc^scid^IL2rg^tm1^/Cavens mice (Fig. 6D and Supplementary Fig. 11D). BZ61 treatment significantly reduced human blasts (hCD45^+^) in PB and BM, at the end of 2-weeks treatment (Fig. 6E, F). CD34^+^ and CD11b^+^ cells derived from human CML CD34^+^ cells were also dramatically diminished by the treatment of BZ61, in comparison to the control groups (Fig. 6G-I). These findings indicated that BZ61 suppressed human CML burden and progression in vivo.

## Discusion

As the emerging non-native component in cells, chimeric proteins encoding by the gene fusions may require an adaptive mechanism that offer self-protection for its maintenance. Here, we discovered that PELI1 is a key factor in maintaining BCR-ABL1 in CML progression. PELI1 is upregulated by BCR-ABL1 via STAT5/FOXP3 axis, which reversely interacts with and protect BCR-ABL1 from the degradation via K63-linked polyubiquitination.

The fate of substrate proteins varies depending on the type of ubiquitination. Proteins tagged with K48-linked ubiquitination are mainly destined for degradation by the 26S proteasome, whereas K63-linkages are predominantly participated in intracellular signaling related to DNA damage repair or cytokine signaling via interactions between proteins [11]. PELI1 promotes the K63-linked ubiquitination of BCR-ABL1 that compete with K48-Ub BCR-ABL modification, which aligns with earlier findings [37, 39, 40]. These findings suggest that PELI1 stabilizes BCR-ABL1 via K63-linked polyubiquitination in CML cells. It is also possible that other E3 ubiquitin ligases-mediated K48-linked ubiquitination of BCR-ABL1 was exclusively masked by the upregulated PELI1 in CML cells.

Consistent with its previously established roles in tumorigenesis [18]. our study demonstrated that PELI1 functions as a co-factor and effector for BCR-ABL1 in CML pathogenesis. Indeed, several E3 ubiquitin ligases have been identified as potential drug target owing to their contributions to the BCR-ABL1 expression in CML. RING-type E3 ligase c-CBL is essential for Arsenic sulfide to trigger the decomposition of BCR-ABL1 in CML [41]. S-phase kinase-associated protein 2 acts as a joint regulator of BCR-ABL1 via K63-linked ubiquitination, which inhibition markedly reduces the expansion of CML cells [24]. The suppression of PELI1 causes BCR-ABL1 to degrade that overcome the compound mutations-mediated TKI resistance. Similar self-protection mechanisms may be also involved in the pathogenesis of other diseases driven by emerging mutants or fusion proteins such as BCR-ABL1.

Our data showed that FOXP3 is upregulated by BCR-ABL1 in CML cells. In line with this, STAT5 was previously reported to bind to the conserved non-coding sequence 2 (CNS2) regions of FOXP3 to regulate its expression [42]. Given that FOXP3 functions as a regulator in other lineage cells, this mechanism may also take responsibility for the progression of BCR-ABL1^+^ lymphoblastic leukemia [43].

The stepwise use of TKIs, each with enhanced abilities to overcome resistance, led to a gradual increase in mutation diversity and complex resistance mutations, mainly occurring on the ABL1 kinase domain [44]. Although Ponatinib has been developed to retain efficacy against mutations that confer resistance to earlier-generation TKIs including the gatekeeper T315I mutation, novel compound mutations (e.g., Y253H + T315I or E255V + T315I) with resistance to Ponatinib have emerged [34, 45]. On the other hand, resistant clones might exist when therapy begins and are selected during treatment rather than being caused by the therapy [44]. Our data demonstrated that PELI1 interacted with the BCR segment of BCR-ABL1 that is independent of the mutations on its ABL1 kinase domain. Therefore, silencing PELI1 impeded the expansion of CML cells driven by native and mutant BCR-ABL1 even those resistance mutations to Ponatinib, exhibiting the potential to overcome TKIs resistance.

PELI1 may be also required for the maintenance of CML-LSCs against cell senescence from DNA damage. Compared to HSCs or bulk CML cells, CML-LSCs exhibit elevated ROS levels [2, 46]. Insufficient repair of oxidative DNA damage impaired self-renewal potential [47]. It is possible that PELI1 help to tolerate high levels of ROS in CML-LSCs, which may explain the upregulated PELI1 in CML-LSCs observed in our study. Due to low levels of BCR-ABL1 expression, CML-LSCs do not depend on it for survival or resistance to TKI therapy [48, 49]. Nonetheless, these BCR-ABL1 maintained blow a certain threshold can upregulate the PELI1 expression in LSCs. Consequently, the self-renewal of CML-LSCs was diminished by genetic or pharmacological blocking of PELI1.

Our data showed that loss of *Peli1* did not affect normal hematopoiesis in young mice (2-months of age). The mild leukocytopenia and anemia in old *Peli1*-knockout mice (>12 Months old) illustrated the functional roles of *Peli1* in stressed hematopoiesis as aging. We also found that CKO-CML mice showed the diminished GFP^+^LSK cells (CML-LSCs) but comparable GFP^-^LSK cells (normal LSC) in bone marrow, as well as similar homing ability with wide type controls. These results support the idea that PELI1 is an ideal target for CML treatment without affecting normal hematopoiesis.

## Conclusion

Our study identified PELI1 as a key regulator for the maintenance of BCR-ABL1 in CML. PELI1 was upregulated by BCR-ABL1 via STAT5/FOXP3, which reversely interacted with and protected BCR-ABL1 from degradation in CML cells. Inhibition of PELI1 is sufficient to ameliorate the disease burden and progression of CML, indicating that targeting PELI1 is a potent strategy for CML treatment that overcomes TKIs resistance and eliminates LSCs.

## Supplementary information

Supplementary information

Supplementary Figures 1

Supplementary Figures 2

Supplementary Figures 3

Supplementary Figures 4

Supplementary Figures 5

Supplementary Figures 6

Supplementary Figures 7

Supplementary Figures 8

Supplementary Figures 9

Supplementary Figures 10

Supplementary Figures 11

Supplementary Table 1

checklist

Unedited blot and gel images

## Data Availability

The data that support the findings of this study are available on request from the corresponding author.
