# Supplementary material for: Inhibition of Pellino-1 reverts the progression and tyrosine kinase inhibitor resistance in chronic myeloid leukemia
Source: Cell Death Dis. 2026 May 5;17(1):593. doi: 10.1038/s41419-026-08799-7 (PMC13287773; doi:10.1038/s41419-026-08799-7)
Supplement: Supplementary file 1 — Supplementary information [file 41419_2026_8799_MOESM1_ESM.docx]

**Inhibition of Pellino-1 reverts the progression and tyrosine kinase inhibitor resistance in chronic myeloid leukemia**

Qian Zhou^1,2,3,4,5^, Guangsen Xu^1,2^, Zhuoran Li^1,2,3,4^, Yan Xu^1,2,3,4^, Jianmin Guan^6^, Zhenyu Li^7^, Mingying Li^8^, Tingjian Zu^9^, Yuan Li^1,2,3,4^, Chunhua Lu^1,2,3,4^, Chunyan Ji^8^, Baobing Zhao^1,2,3,4^*

^1^Key Lab of Chemical Biology (MOE), School of Pharmaceutical Sciences, Cheeloo College of Medicine, Shandong University, Jinan, Shandong, 250012, China;

^2^State Key Laboratory of Discovery and Utilization of Functional Components in Traditional Chinese Medicine, Cheeloo College of Medicine, Shandong University, Jinan, Shandong, 250012, China;

^3^NMPA Key Laboratory for Technology Research and Evaluation of Drug Products, School of Pharmaceutical Sciences, Cheeloo College of Medicine, Shandong University, Jinan, Shandong, 250012, China;

^4^Department of Pharmacology, School of Pharmaceutical Sciences, Cheeloo College of Medicine, Shandong University, Jinan, Shandong, 250012, China;

^5^Department of Pharmacy, Shandong Provincial Key Medical and Health Discipline of Clinical Pharmacy, Shandong Provincial Third Hospital, Shandong University, Jinan 250013, Shandong, China;

^6^Department of Hematology, Heze Municipal Hospital, Heze, Shandong, 274031, China;

^7^Department of Pharmacy, Shandong Provincial Hospital Affiliated to Shandong First Medical University, Jinan, Shandong, 250012, China;

^8^Department of Hematology, Qilu Hospital, Cheeloo College of Medicine, Shandong University, Jinan, Shandong, 250012, China;

^9^School of Stomatology, Shandong First Medical University & Shandong Academy of Medical Sciences, Jinan, Shandong, 250062, China.

*Correspondence author, Phone: 86-531-88382176, Fax: 86-531-88382176

E-mail: [baobingzh@sdu.edu.cn](mailto:baobingzh@sdu.edu.cn) (Baobing Zhao).

**Supplemental Information**

**Inhibition of Pellino-1 reverts the progression and tyrosine kinase inhibitor resistance in chronic myeloid leukemia**

**This PDF file includes:**

Supplemental Materials and Methods

Supplementary Fig. 1 to 11

Supplementary Table 2 to4

Captions for Supplementary Table 1

**Supplemental** **Materials and Methods**

**Generation of Peli1 conditional knockout mice**

We made *Peli1* conditional knockout mice via CRISPR/Cas9-mediated genome engineering. Briefly, Cas9 mRNA, sgRNA and donor were co-injected into zygotes. sgRNAs (5’-AGCACATGTTAATCTCA-3’, 5’-ACCGAGGGACCTAAACTCC-3’) directed Cas9 endonuclease cleavage in intron 2-3 and intron 4-5 and created a double-strand break. LoxP sites were inserted into intron 2-3 and intron 4-5 respectively by homologous recombination. LoxP sites were verified by DNA sequencing.

Chimeric mice were generated by standard methods. *Peli1* deletion was achieved by crossing with transgenic Vav-cre mice purchased from JAX Lab (B6.Cg-Commd10Tg (Vav1-icre) A2Kio/J), by which Exon 3-4 floxed by loxP sites were deleted in hematopoietic cells. This led to the disruption of PELI1 expression. PELI1^fl/fl^ littermate mice were genotyped by PCR with primers *Peli1*-tF1 (5’-CTGGGTGTGTGATACATGCC-3’) and *Peli1*-tR1 (5’- CAGTGTCTGGCCTTGTAAAGTC-3’).

Excision after Vav-Cre recombination was confirmed by PCR with primers to detect a portion that remains post-excision (*Peli1*-null-TF1:5’-GCCAGTGTCATTCTGGGACTTG-3’ and *Peli1*-null-TR1: 5’- GAGGAAGGTAGGAGGTAGTGCC-3’).

**Cell Culture**

Ba/F3 and K562 cells were purchased from the Chinese Academy of Medical Sciences, and cultured in RPMI 1640 medium (BasalMedia, Cat #L210KJ, China) supplemented with 10% fetal bovine serum (SeraPure, Cat #SE141-500, China). KBM5 and KBM5-T315I cells were kindly provided by Jingxuan Pan (Sun Yat-sen University), and cultured in IMDM (Thermo, Cat #12440053, USA) supplemented with 10% fetal bovine serum.

**Co-immunoprecipitation and mass spectrometry analysis**

Cell lysates were obtained and immunoprecipitated with beads conjugated with indicated antibodies as previously described methods[1]. The beads were then washed with IP wash buffer (25 mM Tris pH 7.5,150 mM NaCl, 150 mM MgCl2, 0.5mM EDTA and 5% Glycerol) five times and boiled at 95 °C in 2× NuPAGE LDS Sample Buffer (Thermo Fisher Scientific, NP0007, USA) for 10 min. Aliquots were analyzed by immunoblotting, in parallel, proteins were identified using mass spectrometry (BGI). The antibodies used in immunoprecipitation are listed in Supplemental Table 4.

**Flow Cytometric analyses**

Single-cell suspensions of bone marrow, spleen and peripheral blood were prepared and stained as previously described [2]. Data was collected on a FACS and was analyzed with NovoExpress software. Antibodies used in this study are shown in Supplemental Table 4.

**Plasmids Construction and Cell Transduction**

Cloning of shRNA into the MSCV retroviral vector was performed as previously described[3]. Plasmids encoding Myc-PELI1, HA-PELI1(human) and its truncations (P1: 1-200aa, P2: 1-287aa, P3: 85-311aa, P4: 288-418aa) were cloned into pcDNA3.1 vector， BCR-ABL1-T315I/E255V were purchased from Miao Ling Plasmid Platform (Wuhan, China). MSCV-BCR-ABL1-IRES-EGFP and its T315I mutant were kindly provided by Prof. Chunyan Ji (Qilu Hospital of Shandong University). HA-tagged BCR (Addgene, #38189, USA) and pSG5-ABL (Addgene, #31284, USA) constructs were from Addgene. Plasmids encoding BCR-ABL1 mutants (E255K, Y253F, M351T, H396P, F317L, F317I, Q252H and V299L) were kindly provided by Prof. Qingsong Liu (Chinese Academy of Sciences). PGL3-basic-PELI1 was constructed with the corresponding sequences amplified from human genomic DNA. Viral packaging and injection were conducted as described previously[4]. All oligonucleotides are shown in Supplemental Table 3.

**Luciferase Reporter Assay**

HEK293T cells (1 x 10^5^) were plated in 6-well plate. When the cells fusioned to 80%, the promoter plasmid pGL3-*Peli1* (1 μg) coupled with plasmid STAT5 (1 μg), FOXP3 (1 μg), or an empty vector (1 μg) were transfected into cells. After 24 hours, cells were lysed and subjected to luciferase reporter assays according to the manufacturer’s instructions (Promega, Cat #E2940, USA) and the results were read by microplate reader. All the experiments were repeated three times.

**CML mouse model**

For the primary CML model, c-Kit^+^ bone marrow cells from 8-week-old C57BL/6 mice were stimulated with cytokines and transduced twice with the MSCV-BCR-ABL1-IRES-EGFP retrovirus, followed by tail vein injection into irradiated (550 cGy) recipient C57BL/6 mice (CD45.2). The average onset of lesions was 2 weeks after BMT. For the second generation CML model, 1 x10^6^ GFP^+^ bone marrow and spleen cells were transplanted to recipients through tail vein injection. For evaluation of the *in vivo* effect of PELI1 deletion, similar approaches were performed excluding PELI1-CKO and wildtype mice as the bone marrow donors.

**Immunofluorescence**

Details of immunofluorescence have previously been described [5]. The antibodies used are shown in Supplemental Table 4.

**Homing**

2×10^6^ GFP^+^LSK cells from indicated mice were sorted and injected into lethally irradiated CD45.1 mice. Total GFP^+^ cells and GFP^+^LSKs in the BM were measured by flow cytometry 16-18 h after transplantation. The specific antibody information is provided in Supplemental Table 4.

**Immunoblotting**

Cells were lysed in RIPA buffer (Beyotime, P0013B, China) with protease and phosphatase inhibitor mixture (Beyotime, P1005, China) for 30 min on ice bath, then centrifuged at 12000 rpm, 4 °C. The protein concentration was quantified by a bicinchoninic acid (BCA) protein kit (Beyotime, P0012S, China). The protein expression levels were determined by staining with primary antibodies.

**mRNA extraction and Quantitative RT-PCR**

Total RNA was extracted using the Trizol reagent according to the manufacturer’s instructions (Invitrogen, 15596018CN, USA). RNA quality and quantity were determined using a NanoDrop and Agilent 2100 bioanalyzer (Thermo Fisher Scientific, USA). RNA was reversely transcribed into cDNA using an RT reagent kit (TaKaRa, RR037A, Japan). A SYBR Green PCR kit was used for quantitative real-time PCR and results were quantified with an Applied Biosystem System (ABI) with appropriate primers. The human housekeeping gene 18S was used as RNA-loading control. Gene expression was determined by the delta CT method (2^-∆∆Ct^). ∆Ct = (Ct target gene – Ct housekeeping).

**FLAG pull-down assay**

HEK293T cells expressing FLAG-BCR-ABL1 were lysed in ice-cold IP lysis buffer (Thermo, 87787, USA) and the FLAG-BCR-ABL1 protein was purified with Protein A/G PLUS-Agarose (Santa Cruz Biotechnology, sc-2003, USA). Then, 10 µg PELI1 protein that were expressed and purified through the prokaryotic system were incubated with 10 µg FLAG-fusion protein in binding buffer (50mM Tris-HCl pH 8.0, 200mM NaCl, 1mM EDTA, 1%(v/v) NP-40, 1mM DTT and 10mM MgCl2) overnight at 4°C with rotation. After washing by wash buffer (25 mM Tris pH 7.5,150 mM NaCl, 150 mM MgCl2, 0.5mM EDTA and 5% Glycerol), the beads were resuspended in loading buffer and subjected to western blot analysis.

**References**

1. Lu Z, Huang L, Li Y, Xu Y, Zhang R, Zhou Q, et al. Fine-Tuning of Cholesterol Homeostasis Controls Erythroid Differentiation. Advanced Science (Weinheim, Baden-Wurttemberg, Germany) 2022; 9(2): e2102669.

2. Wen QJ, Yang Q, Goldenson B, Malinge S, Lasho T, Schneider RK, et al. Targeting megakaryocytic-induced fibrosis in myeloproliferative neoplasms by AURKA inhibition. Nat Med 2015; 21(12): 1473-1480.

3. Zhao B, Mei Y, Cao L, Zhang J, Sumagin R, Yang J, et al. Loss of pleckstrin-2 reverts lethality and vascular occlusions in JAK2V617F-positive myeloproliferative neoplasms. The Journal of Clinical Investigation 2018; 128(1): 125-140.

4. Zhao B, Mei Y, Schipma MJ, Roth EW, Bleher R, Rappoport JZ, et al. Nuclear Condensation during Mouse Erythropoiesis Requires Caspase-3-Mediated Nuclear Opening. Developmental Cell 2016; 36(5): 498-510.

5. Zhou Q, Kim SH, Pérez-Lorenzo R, Liu C, Huang M, Dotto GP, et al. Phenformin Promotes Keratinocyte Differentiation via the Calcineurin/NFAT Pathway. The Journal of Investigative Dermatology 2021; 141(1): 152-163.

**Supplemental Figures**

**Supplementary Fig. 1 PELI1 protected BCR-ABL1 from degradation via K63-linked polyubiquitination.** (**A**) Statistical analysis of *PELI1* mRNA expression in CD34^+^ bone marrow (BM) cells obtained from CML patients (n=12) and healthy donors (n=3) using published RNA-seq data (GSE47927). Data were presented as mean±SD. (**B**) Co-IP analysis of PELI1 binding to BCR-ABL1 in HEK293T cells co-transfected with PELI1 (HA-tagged) and BCR-ABL1 (FLAG-tagged) plasmids. (**C**) Co-IP analysis of PELI1 binding to ABL1 segment of BCR-ABL1 using anti-Myc antibody in HEK293T cells co-transfected with HA-ABL and Myc-human PELI1 plasmids. (**D**) Quantitative PCR analysis of *BCR-ABL1* mRNA level in K562 cells transduced with *PELI1* shRNA. shNC represents a non-targeting shRNA. Data were presented as mean±SD from three independent experiments. (**E**) Immunoblotting analysis of BCR-ABL1 in K562 transduced with retroviruses encoding indicated shRNAs after MG132 (10 μM) treatment for 4 h. shNC represents a non-targeting shRNA. GAPDH was used as the loading control. (**F-G**) Immunoblotting and quantitative PCR analysis of BCR-ABL1 in K562 cells transduced with retroviral constructs encoding Myc-human PELI1 or empty vector (OE-C) plasmids. Data were presented as mean±SD from three independent experiments. (**H-I**) Immunoblotting analysis of BCR-ABL1 in K562 cells with PELI1 overexpression upon cycloheximide (CHX, 10 μM) treatment. BCR-ABL1 levels were normalized to the change of β-Tubulin. Data were presented as mean±SD from three independent experiments. P value was determined by two-way ANOVA. (**J**) Immunoblotting analysis of the K48-linked polyubiquitination level of BCR-ABL1 immunoprecipitated by anti-FLAG antibody from HEK293T cells co-overexpressing with BCR-ABL1 (FLAG-tagged), PELI1 or PELI1△C(Myc-tagged) and K48 ubiquitin (HA-tagged) plasmids.

**Supplementary Fig. 2** **BCR-ABL1 regulated PELI1 expression via STAT5/FOXP3 transcriptional activation in CML.** (**A**) Schematic strategy for CML mouse model. (**B**) Immunoblotting analysis of indicated proteins in Ba/F3 cells transduced with retroviral constructs encoding BCR-ABL1 or empty vector (OE-C), HSC70 was used as the loading control. (**C**) Quantitative PCR analysis of *Peli1* mRNA level in Ba/F3 cells as in B. Data were presented as mean±SD from three independent experiments. (**D**) Immunoblotting analysis of indicated protein levels in K562 cells with STAT5 inhibitor (STAT5 IN) treatment for 10 h. GAPDH was used as the loading control. (**E**) Quantitative PCR analysis of *PELI1* mRNA levels in cells as in D. Data were presented as mean±SD from three independent experiments. (**F**) Schematic of the potential binding sites of STAT5 in PELI1 promoter. Black blocks with “TTC*NNN*GAA” represent the reported conserved sequences of STAT5 binding to the target genes. (**G**) The distribution of STAT5-binding peaks within PELI1 gene regions from ENCODE database (<https://www.encodeproject.org/>). (**H**) Luciferase reporter assays of *Peli1* promoter in HEK293T cells co-transfected with the wide type (WT), constitutively active (CA) and domain negative (DN) STAT5 and *Peli1* promoter luciferase reporter. Data were presented as mean±SD from three independent experiments. OE-C represents overexpression of empty vector. (**I**) Quantitative PCR analysis of *PELI1* mRNA levels in K562 cells transduced with retroviruses encoding indicated shRNAs. shNC represents a non-targeting shRNA. Data were presented as mean±SD from three independent experiments. (**J**) Immunoblotting analysis of indicated proteins in K562 cells transduced with retroviruses encoding *FOXP3* shRNAs. shNC represents a non-targeting shRNA. (**K**) Quantitative PCR analysis of *FOXP3* mRNA levels in K562 cells transduced with the indicated shRNAs. shNC represents a non-targeting shRNA. BA represents BCR-ABL1. Data were presented as mean±SD from three independent experiments. (**L**) Quantitative PCR analysis of *FOXP3* mRNA levels in K562 cells treated with Imatinib for 10 h. Data were presented as mean±SD from three independent experiments.

**Supplementary Fig. 3 Silencing BCR-ABL1 promotes the CML cell senescence.** (**A**) SA-β-gal staining analysis in K562 cells with indicated shRNAs. shNC represents a non-targeting shRNA. Data were presented as mean±SD from three independent experiments. (**B**) Western blot analysis of the indicated protein levels in K562 cells as in A. BA: BCR-ABL1. shNC represents a non-targeting shRNA. (**C**) SA-β-gal staining analysis in K562 cells with Imatinib (IM, 10 μM) treatment. Data were presented as mean±SD from three independent experiments. (**D**) Western blot analysis of the indicated protein levels in K562 cells with 10 h of Imatinib (IM) treatment. GAPDH was used as the loading control.

**Supplementary Fig. 4 PELI1 was upregulated in Po-resistant** **CML cells.** (**A**) Co-IP analysis of PELI1 binding to BCR-ABL1^T315I^ mutation (BA^T315I^) in HEK293T cells co-transfected with BCR-ABL1^T315I^ (FLAG-tagged) and PELI1 (HA-tagged). (**B**) Quantitative PCR analysis of *BCR-ABL1* in Ba/F3 cells transduced with retroviral constructs encoding indicated BCR-ABL1 mutants or empty vector (OE-C). Data were presented as mean±SD from three independent experiments. (**C**) Western blot analysis of the indicated protein levels in Ba/F3 cells transduced with retroviral constructs encoding BCR-ABL1^T315I^ and BCR-ABL1^T315I+E255V^ upon the treatment of Imatinib Ponatinib (Po) for 10 h. HSC70 was used as the loading control. Data were presented as mean±SD from three independent experiments.

**Supplementary Fig. 5** **PELI1 played roles in stressed hematopoiesis.** (**A**) Schematic of *Peli1*^fl/fl^; Vav-Cre gene-targeting strategy. (**B**) Western blot analysis of PELI1 protein levels in mononuclear bone marrow cells from the WT (*Peli1*^fl/fl^), HET (*Peli1*^fl/+; Vav-cre^) and CKO (*Peli1*^fl/fl; Vav-cre^) mice. (**C**) Peripheral blood analysis of indicated mice as in A at the age of 2 months and 12 months respectively. Data were shown as mean±SD. Each dot represents one mouse. (**D**) Quantification of homing efficiency of LSC (GFP^+^LSK^+^) from indicated mice as in Fig. 5A. Data were shown as means±SD. NS represents no significant by Student’s t-test. (**E**) Flow cytometry analysis of leukemia cells (GFP^+^Gr1^+^) in peripheral blood mononuclear cells from the mice as in Fig. 5A. (**F**) Representative H&E stains of spleen from indicated mice as in Fig. 5A. (**G-H**) The gross pathology and H&E staining of lungs from the mice as in E. (**I**) Kaplan-Meier survival curves of indicated mice after second round transplantation as in Fig. 5A. n=8 mice per group. *P* value was determined by the Log-rank test. (**J**) Representative flow cytometry histograms for LSC (GFP^+^LSK^+^) cells and normal HSC (GFP^-^LSK^+^) cells in bone marrow from the mice as in Fig. 5A. (**K**) Statistical analysis of normal HSC (GFP^-^LSK^+^) in the bone marrow as in J. Each dot represents one mouse. Data were presented as mean±SD from five independent experiments.

**Supplementary Fig. 6** **Loss of PELI1** **ameliorated the disease burden and progression in BCR-ABL1^T315I^-deriven CML.** (**A**) Schematic representation of BCR-ABL1^T315I^-driven CML mice model with wild-type (WT) or *Peli1* conditional knockout (CKO) mice bone marrow cells. (**B**) Flow cytometry analysis of leukemia cells (GFP^+^ or GFP^+^Gr1^+^) in peripheral blood mononuclear cells (PBMC) from the indicated mice as in A. Each dot represents one mouse. Data were shown as means ± SD. (**C**) Flow cytometry analysis of leukemia cells (GFP^+^, GFP^+^LSK or GFP^+^Gr1^+^) in the bone marrow from indicated mice as in A. Each dot represents one mouse. Data were presented as mean±SD. (**D**) Representative spleen of the indicated mice as in B was shown. (**E**) Statistical analysis of spleen weight from indicated mice as in A. Each dot represents one mouse. Data were presented as mean±SD. (**F**) Flow cytometry analysis of leukemia cells (GFP^+^, GFP^+^LSK or GFP^+^Gr1^+^) in the spleen from indicated mice as in A. Each dot represents one mouse. Data were presented as mean±SD. (**G**) H&E staining of lungs from indicated mice as in A. (**H**) Kaplan-Meier survival curves of indicated mice as in A. n=8 mice per group. *P* value was determined by Log-rank test.

**Supplementary Fig. 7** **Loss of PELI1 ameliorated the progression of Ponatinib-resistant CML.** (**A**) Schematic representation of BCR-ABL1^T315I+E255V^-driven CML mice model with wild-type (WT) or *Peli1* conditional knockout (CKO) mice bone marrow cells. (**B**) Flow cytometry analysis of leukemia cells (GFP^+^ or GFP^+^Gr1^+^) in peripheral blood mononuclear cells (PBMC) from the indicated mice as in A. Each dot represents one mouse. Data was shown as means ± SD. (**C**) Flow cytometry analysis of leukemia cells (GFP^+^, GFP^+^LSK^+^ or GFP^+^Gr1^+^) in the bone marrow from the indicated mice as in A. Each dot represents one mouse. Data were presented as mean±SD. (**D**) The representative spleen of the indicated mice as in A was shown. (**E**) Statistical analysis of spleen weight from the indicated as in D. Each dot represents one mouse. Data were presented as mean±SD. (**F**) Flow cytometry analysis of leukemia cells (GFP^+^, GFP^+^LSK^+^ or GFP^+^Gr1^+^) in the spleen from the indicated as in A. Each dot represents one mouse. Data were presented as mean±SD. (**G**) H&E staining of lungs from the indicated mice as in A. (**H**) Kaplan-Meier survival curves of indicated mice. n=8 mice per group. *P* value was determined by the Log-rank test.

**Supplementary Fig. 8** **PELI1 inhibitor screening.** (**A**) Screening strategy of PELI1 small molecule inhibitors. Primary screening was performed with ELISA assays in K562 cells based on the decrease of SNAIL and SLUG, since SNAIL and SLUG had been documented as the substrates of PELI1. Hit compounds were then selected to bind with PELI1 in vitro using cellular thermal shift assay, followed by the identification to inhibit K562 cell proliferation. (**B**) Surface Plasmon Resonance (SPR) analysis revealed the dissociation constant (*Kd*) for PELI1 and BZ61 calculated by the built-in evaluation software. (**C**) Thermal shift assay showed the binding of PELI1 with BZ61 i*n vitro*. The purified PELI1 protein was incubated with an indicated dose of BZ61 at 48℃ for 5 min respectively, followed by Western blotting for the soluble proteins. Data were presented as mean±SD from three independent experiments. **(D)** Immunoblotting analysis of Snail and STAT3 proteins in K562 cells with BZ61 treatment. GAPDH was used as the loading control. **(E-F)** Co**-**IP assay analyses of K63- mediated polyubiquitination of STAT3 (E) and SNAIL (F) in HEK293T cells transfected with MYC-STAT3 or MYC-SNAIL, Flag-PELI1 and HA-K63 ubiquitin plasmids after BZ61 treatment. **(G-H)** In vitro ubiquitination assays with recombinant PELI1 and SNAIL and STAT3 proteins in the presence of ubiquitin (Ub), E1 and E2 (UbcH5A).

**Supplementary Fig. 9 BZ61 ameliorated the CML phenotypes.**

(A) K562 cells were seeded into 96-well microtiterplates (5X10^3^ cells/well) and treated with different concentrations of BZ61 then incubated at 37℃ for 24h. CCK-8 assay was performed at 540 nm and IC50 value was measured. (**B**) Co-IP analysis of PELI1 (HA-tagged) binding to BCR-ABL1 (FLAG-tagged) in HEK293T cells with BZ61 (10 μM) for 10 h. (**C**) Quantitative PCR analysis of *PELI1* mRNA levels in K562 cells with BZ61 for 10 h. Data were presented as mean±SD from three independent experiments. (**D-E**) SA-β-gal staining analysis in K562 cells with BZ61 (10 μM) treatment for 24 h. Data were presented as mean±SD from three independent experiments. (**F**) Schema of strategy to evaluate the effects of BZ61 on CML mice. GFP^+^ bone marrow cells from BCR-ABL1-driven CML mice (1x10^6^) were injected into the tail veins of the sublethally irradiated recipient mice (550 cGry), followed by the treatment of IM (100 mg/kg/day, i.g.), BZ61 (25 mg/kg, administered intraperitoneally every other day) and their combination respectively. (**G-H**) Flow cytometry analysis of GFP^+^ and GFP^+^Gr1^+^ cells in bone marrow from mice as in F. Each dot represents one mouse. Data were presented as mean±SD. (**I-K**) Flow cytometry analysis of GFP^+^, GFP^+^Gr1^+^ and GFP^+^LSK^+^ in spleen from the mice as in F. Each dot represents one mouse. Data were presented as mean±SD.

**Supplementary Fig. 10** **BZ61 ameliorated the progression of** **Ponatinib-resistant CML.** (**A**) Schematic strategy for the therapeutic effects of BZ61 (25 mg/kg, administered intraperitoneally every other day), Ponatinib (Po, 25 mg/kg/day, i.g.) and their combination on the BCR-ABL1^T315I+E255V^-driven CML miceA. (**B**) Kaplan-Meier survival curves of indicated mice as in A. n=8 mice per group. The p value was determined by the Log-rank test. (**C**) Statistical analysis of body weights of the indicated mice as in A. (**D-G**) Flow cytometry analysis of leukemia cells (GFP^+^ or GFP^+^Gr1^+^) in peripheral blood (PB) and bone marrow (BM) from mice as in A. Each dot represents one mouse. (**H**) Flow cytometry analysis of CML-LSC cells (GFP^+^LSK^+^) in bone marrow from the mice as in A. (**I**) The representative spleen of indicated mice as in A was shown. (**J**) Spleen weights of the indicated mice as in A. (**K-L**) Flow cytometry analysis of leukemia cells (GFP^+^ or GFP^+^Gr1^+^) in the spleen from the mice as in A. Each dot represents one mouse.

**Supplementary Fig. 11** **Silencing PELI1 inhibited the proliferation of CML-CD34^+^ cells.** (**A**) Statistical analysis of cell proliferation in CD34^+^ bone marrow cells from CML patient (CML#13 in Supplementary Table 2) with PELI1 knockdown. Data were presented as mean±SD from three independent experiments. *P* value was determined by two-way ANOVA. (**B**) Statistical analysis of colonies derived from the cells as A. Data were presented as mean±SD from three independent experiments. (**C**) Statistical analysis of cell proliferation in CD34^+^ bone marrow cells from normal healthy donors (Normal#9 in Supplementary Table 2) with PELI1 knockdown. Data were presented as mean±SD from three independent experiments. *P* value was determined by two-way ANOVA. (**D**) Quantitative PCR analysis of indicated mRNA levels in CD34^+^ BM cells from CML patient (CML#13 in Supplementary Table 2) and healthy donor (Normal#9 in Supplementary Table 2). Data were presented as mean±SD from three independent experiments.

**Supplemental Tables**

**Supplementary Table 1. Identified proteins from co-immunoprecipitation with BCR-ABL1 antibody in K562 cells.**

**Supplementary Table 2. The information of CML patients and healthy control.**

| **ID** | **Material** | **Gender** | **Disease phase** | **Gene rearrangement** |
| --- | --- | --- | --- | --- |
| CML#1 | PB | Female | CML-CP | P210(+)/P190(-)/P230(-) |
| CML#2 | BM | Female | CML-BP | P210(+)/P190(-)/P230(-) |
| CML#3 | BM | Male | CML-CP | P210(+)/P190(-)/P230(-) |
| CML#4 | BM | Male | CML-CP | P210(+)/P190(-)/P230(-) |
| CML#5 | BM | Male | CML-CP | P210(+)/P190(-)/P230(-) |
| CML#6 | BM | Male | CML-CP | P210(+)/P190(-)/P230(-) |
| CML#7 | BM | Female | CML-CP | P210(+)/P190(-)/P230(-) |
| CML#8 | BM | Male | CML-CP | P210(+)/P190(-)/P230(-) |
| CML#9 | BM | Male | CML-CP | P210(+)/P190(-)/P230(-) |
| CML#10 | BM | Male | CML-CP | P210(+)/P190(-)/P230(-) |
| CML#11 | BM | Male | CML-CP | P210(+)/P190(-)/P230(-) |
| CML#12 | BM | Male | CML-CP | P210(+)/P190(-)/P230(-) |
| CML#13 | BM | Male | CML-CP | P210(+)/P190(-)/P230(-) |
| Normal#1 | PB | Female | Normal |  |
| Normal#2 | PB | Female | Normal |  |
| Normal#3 | PB | Female | Normal |  |
| Normal#4 | PB | Male | Normal |  |
| Normal#5 | PB | Male | Normal |  |
| Normal#6 | BM | Female | Normal |  |
| Normal#7 | BM | Female | Normal |  |
| Normal#8 | BM | Female | Normal |  |
| Normal#9 | PB | Male | Normal |  |

**Supplementary Table 3. Primer sequences used in this study.**

| **Primer** | **species** | **Sequence** |
| --- | --- | --- |
| PELI1-F | Mus musculus | TTATGCAGCCTTGCCACTCA |
| PELI1-R | Mus musculus | AGCTCTGGGATCAGGGGATT |
| BCR-ABL1-F | Homo | TCCACTCAGCCACTGGATTTAA |
| BCR-ABL1-R | Homo | TGAGGCTCAAAGTCAGATGCTACT |
| 18S-F | Homo/Mus | GCAATTATTCCCCATGAACG |
| 18S-R | Homo/Mus | GGCCTCACTAAACCATCCAA |
| FOXP3-F | Homo | ACAGCACATTCCCAGAGTTCC |
| FOXP3-R | Homo | TCATTGAGTGTCCGCTGCTT |
| Foxp3-F | Mus musculus | CCCATCCCCAGGAGTCTTG |
| Foxp3-R | Mus musculus | ACCATGACTAGGGGCACTGTA |
| YY1-F | Homo | TGGCAGAATTTGCTAGAATGAAGC |
| YY1-R | Homo | TGGCCGAGTTATCCCTGAAC |
| IRF2-F | Homo | GGGGCTCAAGTGGCTTAACA |
| IRF2-R | Homo | CCTGGTTGATGCTTTCCTGT |
| CEBPB-F | Homo | GCACAGCGACGAGTACAAGA |
| CEBPB-R | Homo | TGCTTGAACAAGTTCCGCAG |
| BRCA1-F | Homo | CCTCTGCTCTGGGTAAAGTTCA |
| BRCA1-R | Homo | TGGTCACACTTTGTGGAGACA |
| CREBBP-F | Homo | GCTGTGGACGCAAGTATGAG |
| CREBBP-R | Homo | GTCACATTCTCGCCCTGGAT |
| EP300-F | Homo | TGCCAAACCAGATGATGCCT |
| EP300-R | Homo | ATAGCCCATAGGCGGGTTGA |
| STAT5B-F | Homo | ATGGCTGTGTGGATACAAGCTCAG |
| STAT5B-R | Homo | TCACGATTGTGCGTGCGGGATC |
| STAT5A-F | Homo | ATGGCGGGCTGGATCCAGG |
| STAT5A-R | Homo | TCATGAGAGGGAGCCTCTGGCA |
| PELI1-F | Homo | TTGTGATGCATCCACGCAAT |
| PELI1-R | Homo | ACAATGTTGCACCACAGAGG |
| BCR-ABL-shRNA#1 | Homo | GAGTTCTTGAAGCATTTCAAA |
| BCR-ABL-shRNA#2 | Homo | CCAGTGGAGATAACACTCTAA |
| PELI1-shRNA#1 | Homo | CTCATTGTCTTAGGGTATAAT |
| PELI1-shRNA#2 | Homo | TTACAAGATGGCTCGTTAATT |
| IRF2-shRNA#1 | Homo | CCAGGAGTAGATAAACCTGAT |
| IRF2-shRNA#2 | Homo | GCAGATAAACTCCAACACGAT |
| C/EBP-beta-shRNA#1 | Homo | CCCGTGGTGTTATTTAAAGAA |
| C/EBP-beta-shRNA#2 | Homo | GCACAGCGACGAGTACAAGAT |
| BRCA1-shRNA#1 | Homo | CCCTAAGTTTACTTCTCTAAA |
| BRCA1-shRNA#2 | Homo | GCCTACAAGAAAGTACGAGAT |
| CREBBP-shRNA#1 | Homo | CCGTTTACCATGAGATCCTTA |
| CREBBP-shRNA#2 | Homo | CGTGCCAAATATGTCTCAGAT |
| EP300-shRNA#1 | Homo | GCCTTCACAATTCCGAGACAT |
| EP300-shRNA#2 | Homo | TACACTAGAGACACCTTGTAT |
| YY1-shRNA#1 | Homo | CCTCCTGATTATTCAGAATAT |
| YY1-shRNA#2 | Homo | GCCTCTCCTTTGTATATTATT |
| FOXP3-shRNA#1 | Homo | CCTCCACAACATGGACTACTT |
| FOXP3-shRNA#2 | Homo | CACACGCATGTTTGCCTTCTT |
| PELI1-shRNA#1 | Mus musculus | GCTCCTTTGAATATGCAATTT |
| PELI1-shRNA#2 | Mus musculus | GCTTTAAGACAGGAGATCAAT |
| PELI1-1(CHIP)-F | Homo | CTTCTGCTGCCCAAATCTGT |
| PELI1-1(CHIP)-R | Homo | AGTGGACCACAGTGTCTACT |

**Supplementary Table 4. Antibodies and commercial reagents used in this study.**

| **Antibody** | **Brand** | **Cat#** |
| --- | --- | --- |
| Myc-Tag(9B11) | CST | 2276S |
| Normal Rabbit IgG | CST | 2729S |
| p21 PolyAb | Proteintech | 10355-1-Ap |
| Phospho-c-Ab1(Y245) | CST | 2861S |
| Pellino-1(D2Z4F) | CST | 31474S |
| Anti-HA-tag mAb | MBL | M180-3 |
| c-Ab1 | CST | 2862S |
| GADPH(D16H11) XP(R) | CST | 5174S |
| Phospho-Stat5(Y694) (C11C5) | CST | 9359S |
| STAT5 (D206Y) | CST | 94205T |
| Anti-HA-Tag | AFFINITY | T0050 |
| Anti-DDDK-tag-FLAG | MBL | M185-3 |
| Anti-DDDK-tag-FLAG | AFFINITY | T0053 |
| HRP-Goat Anti-Rabbit IgG | Jackson immuno | 111-035-144 |
| HRP-Goat Anti-Mouse IgG | Jackson immuno | 115-035-003 |
| APC Annexin V  FITC-Annexin V | Biolegend  Biolegend | 640920  640906 |
| PE anti-human Ki-67 | Biolegend | 350503 |
| PE-Sca-1 | eBioscience | 12-5981 |
| APC-CD117 (c-Kit) | eBioscience | 17-1171 |
| Pacific Blue™ anti-mouse Ly-6G/Ly-6C (Gr-1) | Biolegend | 108429 |
| APC/Cy7 CD117 (c-kit) | Biolegend | 105826 |
| PE anti-mouse/human CD11b | Biolegend | 101207 |
| PE/Cy7 anti-mouse Ly-6A/E (Sca-1) | Biolegend | 108114 |
| Pacific Blue™ anti-mouse Lineage Cocktail | Biolegend | 133306 |
| Pacific Blue™ anti-mouse CD45.1 | Biolegend | 110722 |
| FITC anti-human CD45 | Biolegend | 304006 |
| APC anti-human CD34 | Biolegend | 343510 |
| PE anti-human CD11b | Biolegend | 982606 |
| K48-linkage Specific Polyubiquitin | CST | 8081T |
| K63-linkage Specific Polyubiquitin | CST | 5621T |
| Myc | Aladdin | Ab116621 |
| Snail | CST | 3879T |
| STAT3 | CST | 12640T |
| γH2AX | RM8145 | Biodragon |
| H2AX | Aladdin | Ab107657 |
| STAT5 | Santa Cruz | sc-835 |
| **Reagents** |  |  |
| Hoechst 33342 | Invitrogen | H1399 |
| Annexin V binding buffer  RBC Lysis Buffer  Imatinib Mesylate  β-galactosidase Assay Kit | Biolegend  Biolegend  MCE  Beyotime Biotechnology | 422201  420301  HY-50946  C1115 |
| Recombinant Human SCF | Peprotech | 300-07 |
| Recombinant Human IL-3 | Peprotech | 200-03 |
| Recombinant Human IL-6 | Peprotech | 200-06 |
| Recombinant Human GM-CSF | Peprotech | 300-03 |
| Recombinant Murine SCF | Peprotech | 250-03 |
| Recombinant Murine IL-3 | Peprotech | 213-13 |
| Recombinant Murine IL-6 | Peprotech | 216-16 |
| EasySep™ Human CD34 Positive Selection Kit II | STEMCELL | 17856 |
| StemSpan™ SFEM | STEMCELL | 09650 |
| MethoCult™ GF M3534 | STEMCELL | 03534 |
| MethoCult™ GF H4434 | STEMCELL | 04434 |
| STAT5 Inhibitor | TargetMol | [T16940](https://www.targetmol.cn/compound/STAT5-IN-2) |
| SimpleChIP® Enzymatic Chromatin IP Kit | CST | 9003 |
| SNAIL | Sangon | D621239 |
| STAT3 | MCE | HY-P701098 |
| IP Lysis Buffer | Thermo | 87787 |
