# Supplementary figures and images for: Inhibition of Pellino-1 reverts the progression and tyrosine kinase inhibitor resistance in chronic myeloid leukemia
Source: Cell Death Dis. 2026 May 5;17(1):593. doi: 10.1038/s41419-026-08799-7 (PMC13287773; doi:10.1038/s41419-026-08799-7)

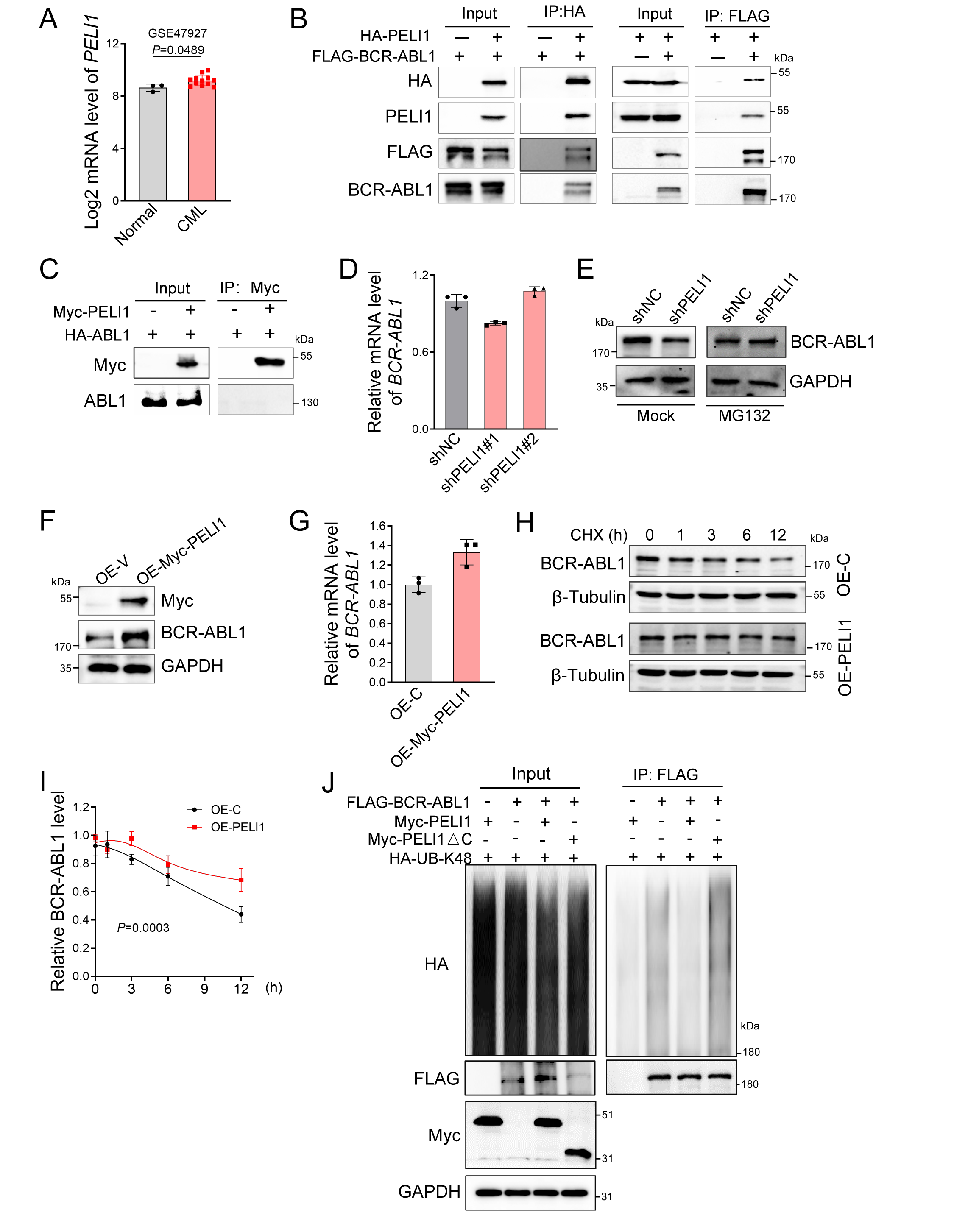

Supplement: Supplementary file 2 — Supplementary Figures 1 [file 41419_2026_8799_MOESM2_ESM.tif]

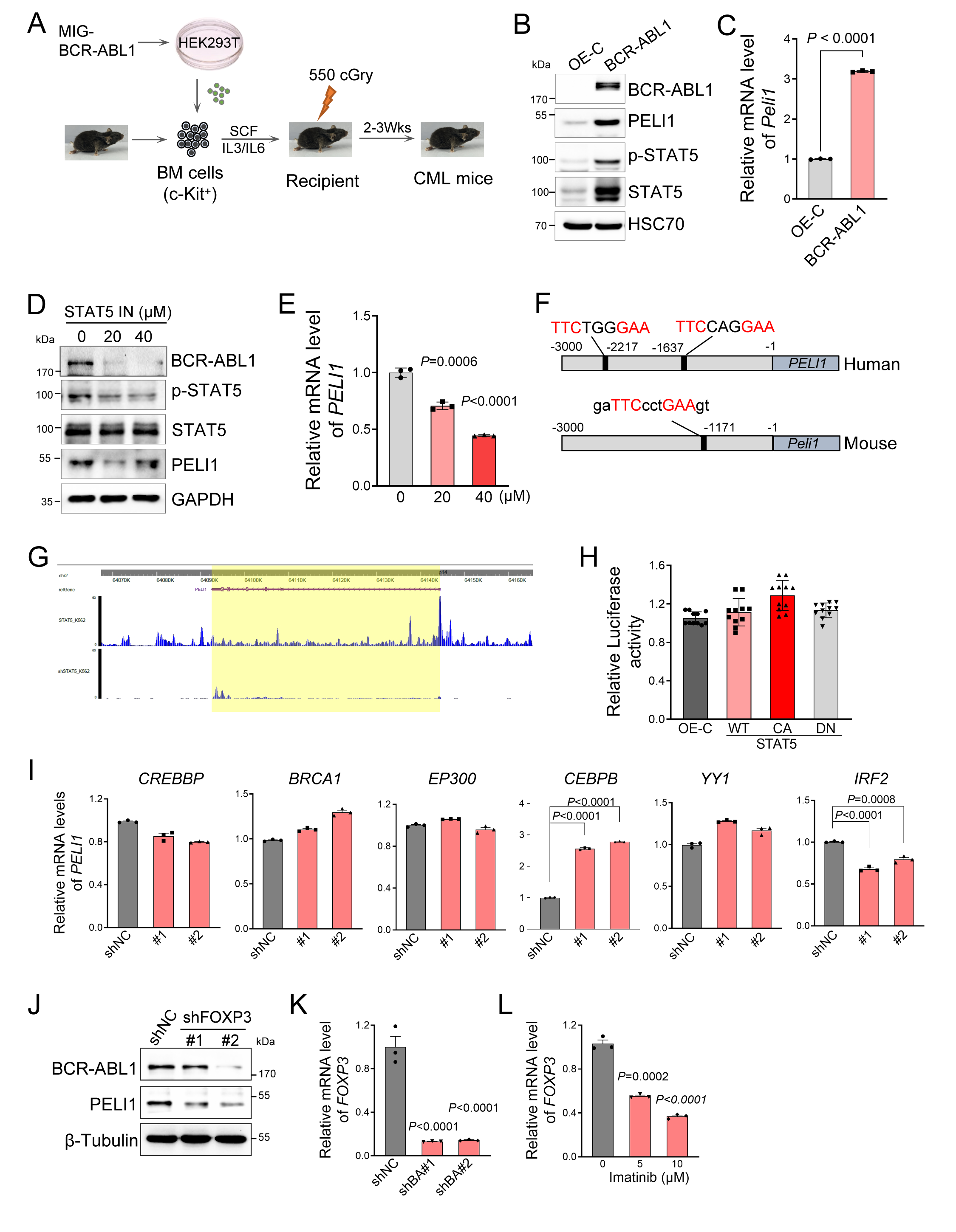

Supplement: Supplementary file 3 — Supplementary Figures 2 [file 41419_2026_8799_MOESM3_ESM.tif]

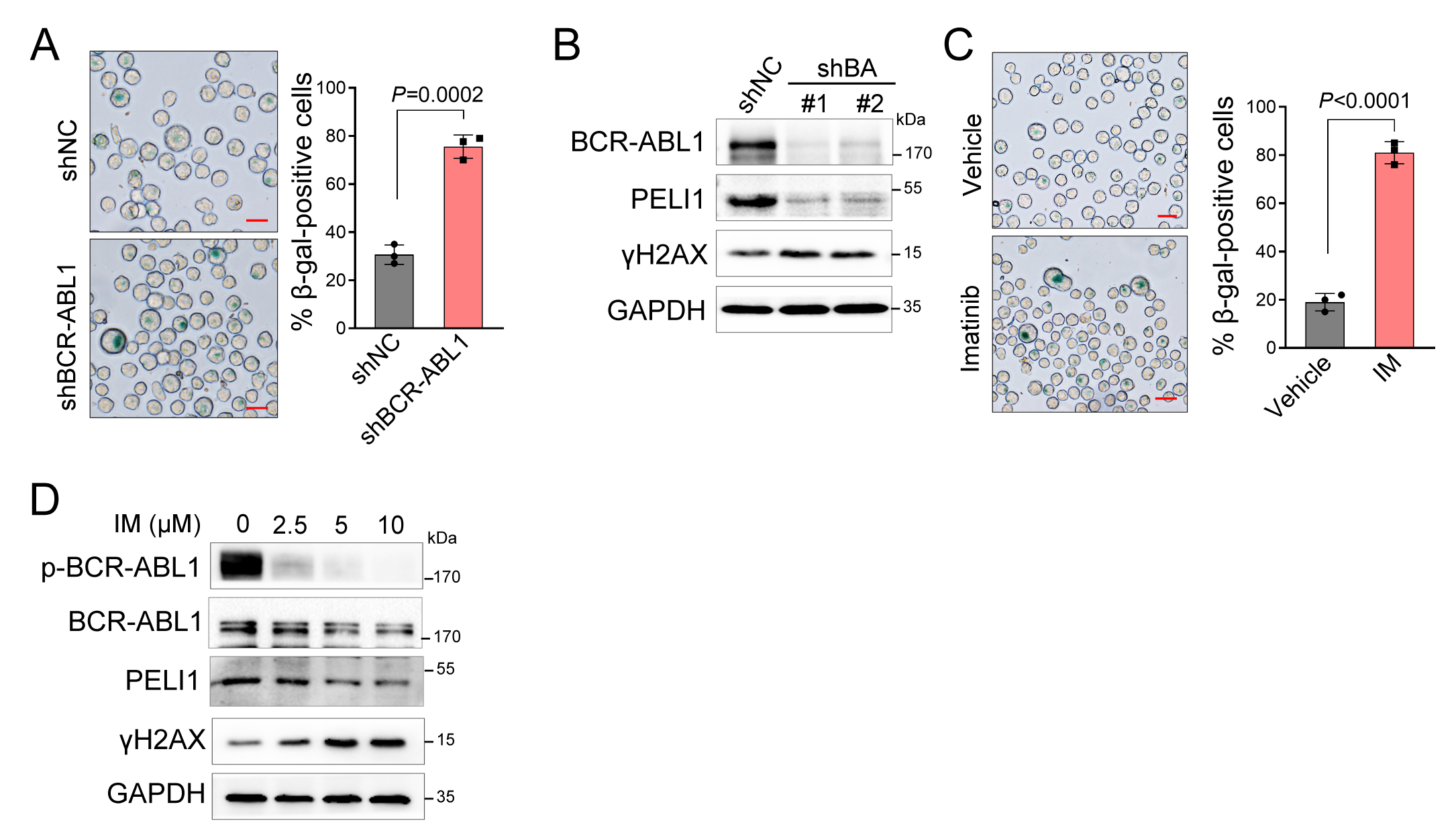

Supplement: Supplementary file 4 — Supplementary Figures 3 [file 41419_2026_8799_MOESM4_ESM.tif]

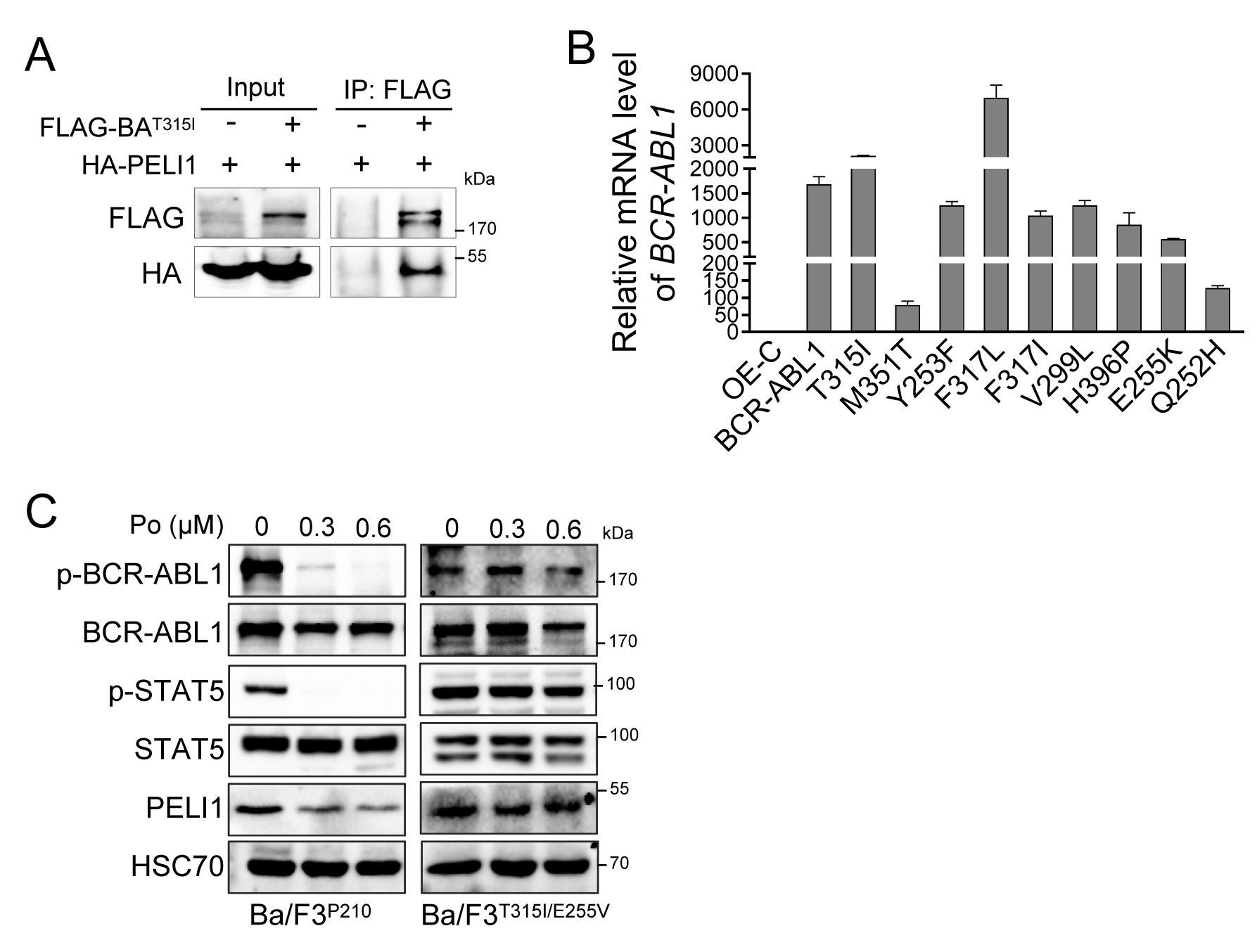

Supplement: Supplementary file 5 — Supplementary Figures 4 [file 41419_2026_8799_MOESM5_ESM.tif]

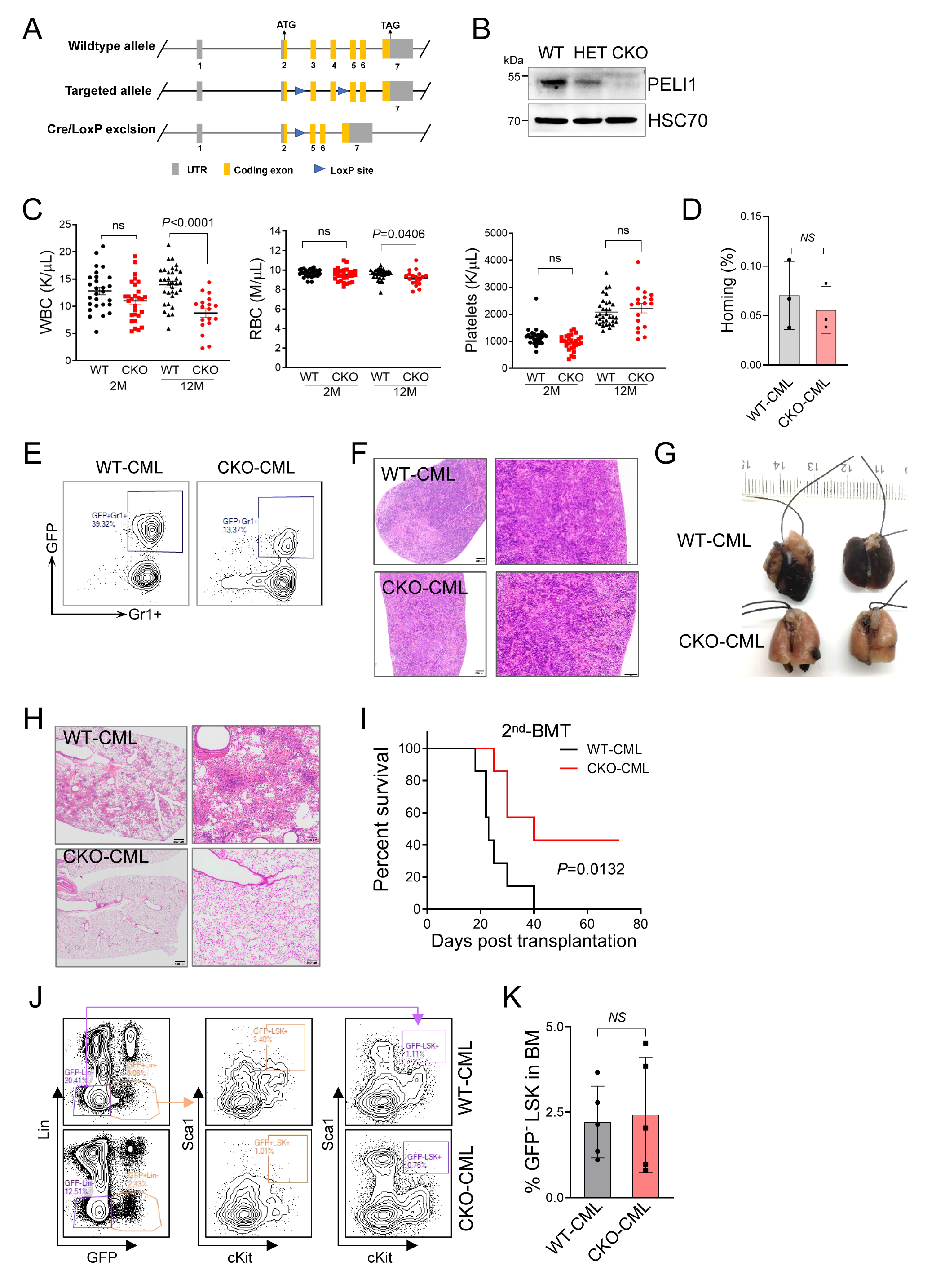

Supplement: Supplementary file 6 — Supplementary Figures 5 [file 41419_2026_8799_MOESM6_ESM.tif]

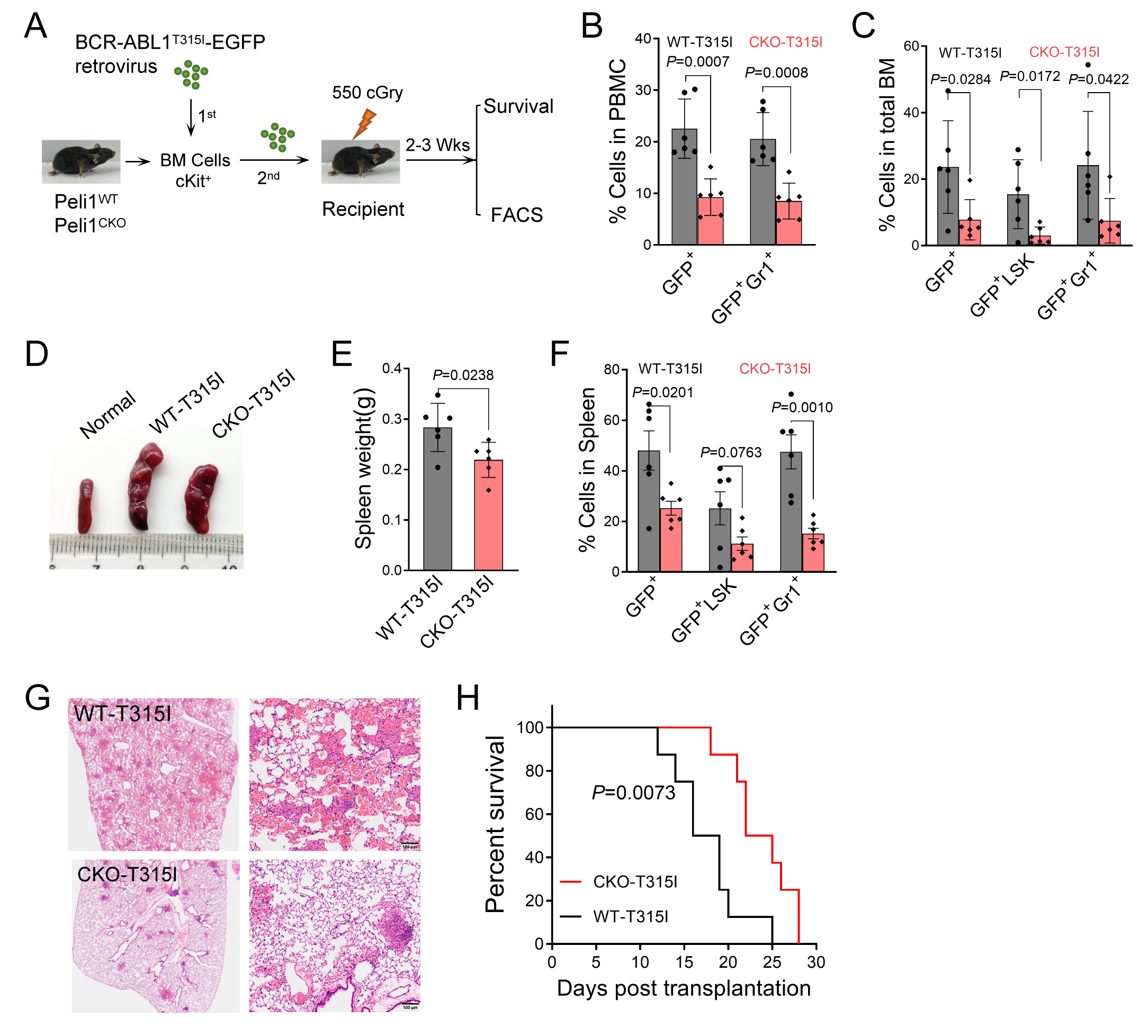

Supplement: Supplementary file 7 — Supplementary Figures 6 [file 41419_2026_8799_MOESM7_ESM.tif]

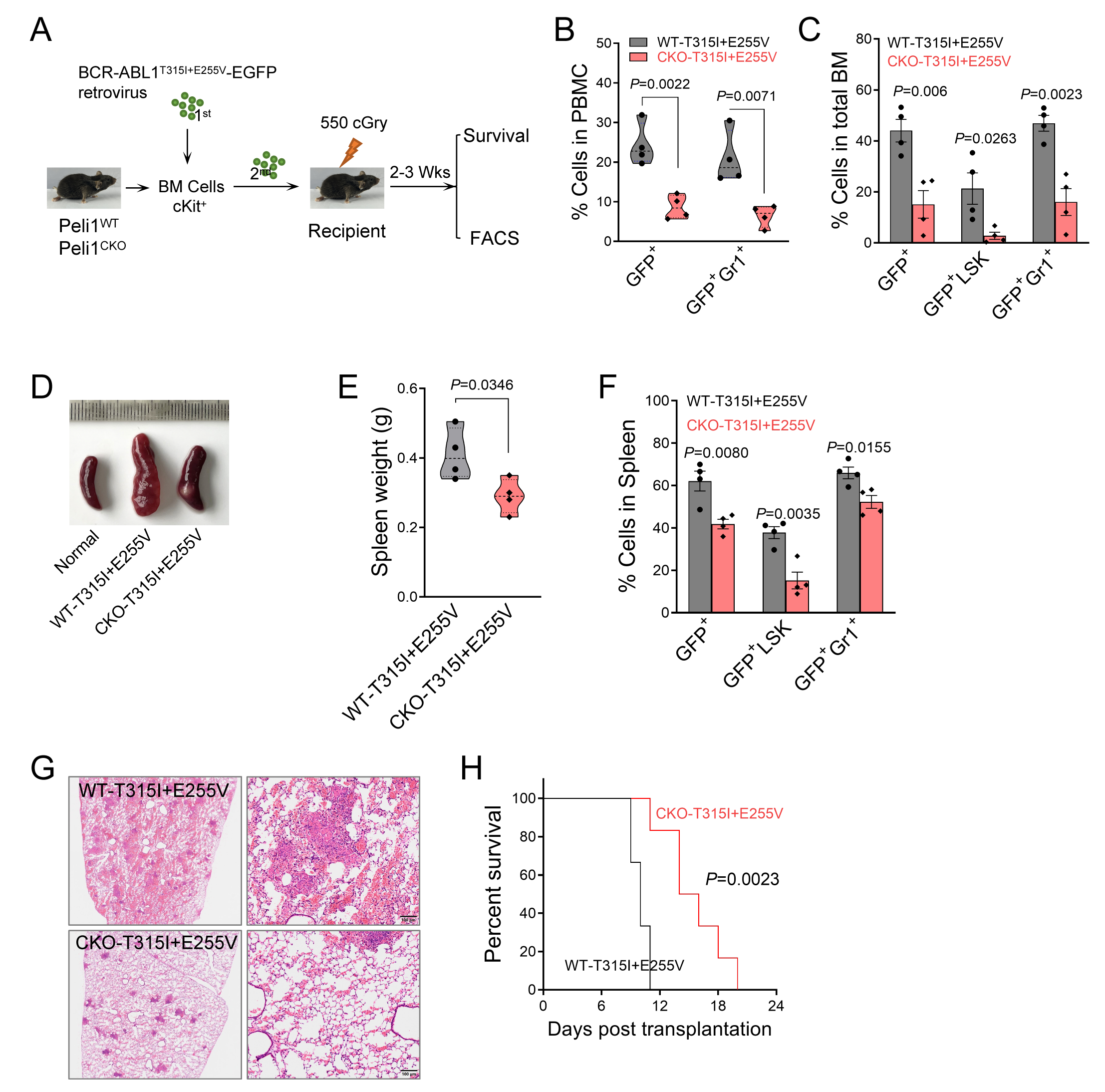

Supplement: Supplementary file 8 — Supplementary Figures 7 [file 41419_2026_8799_MOESM8_ESM.tif]

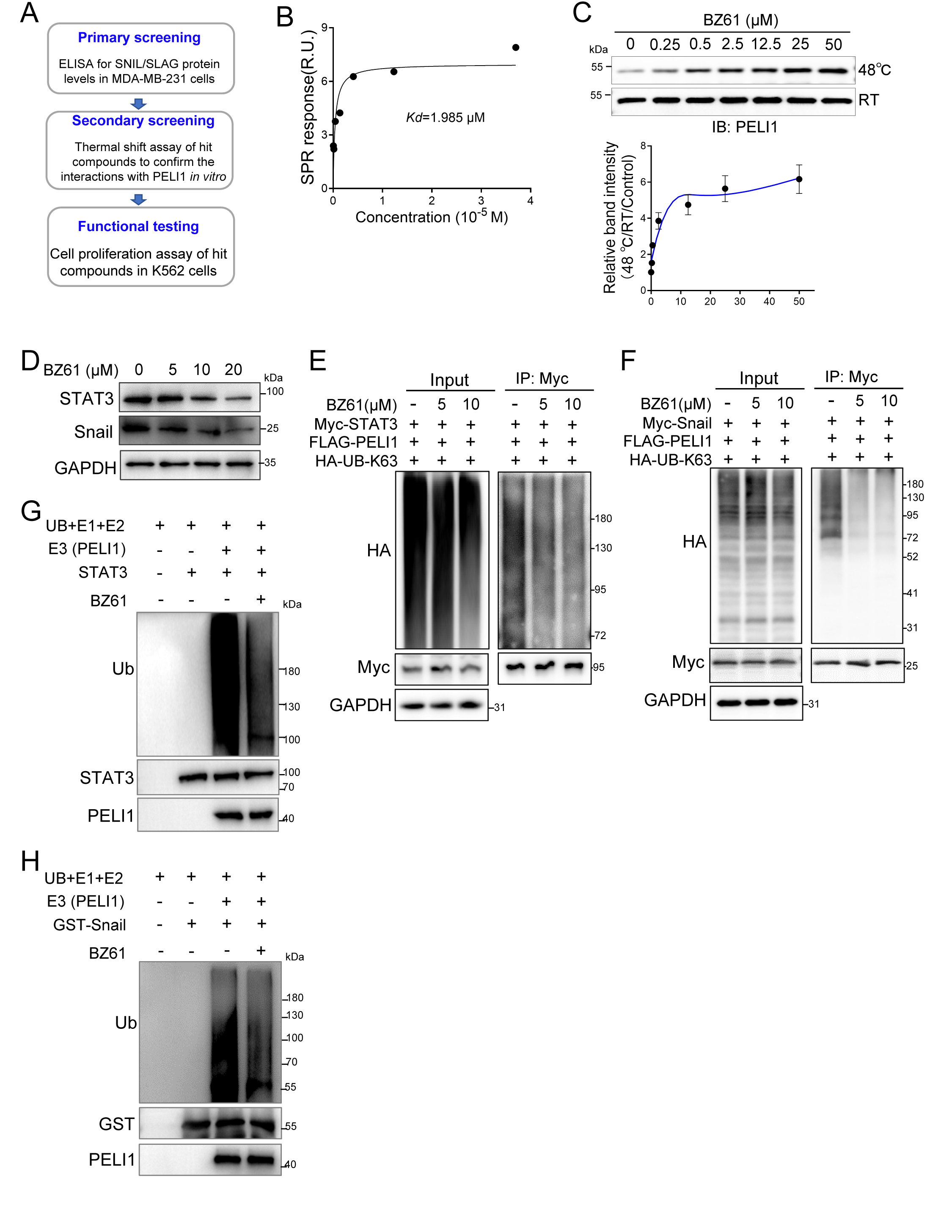

Supplement: Supplementary file 9 — Supplementary Figures 8 [file 41419_2026_8799_MOESM9_ESM.tif]

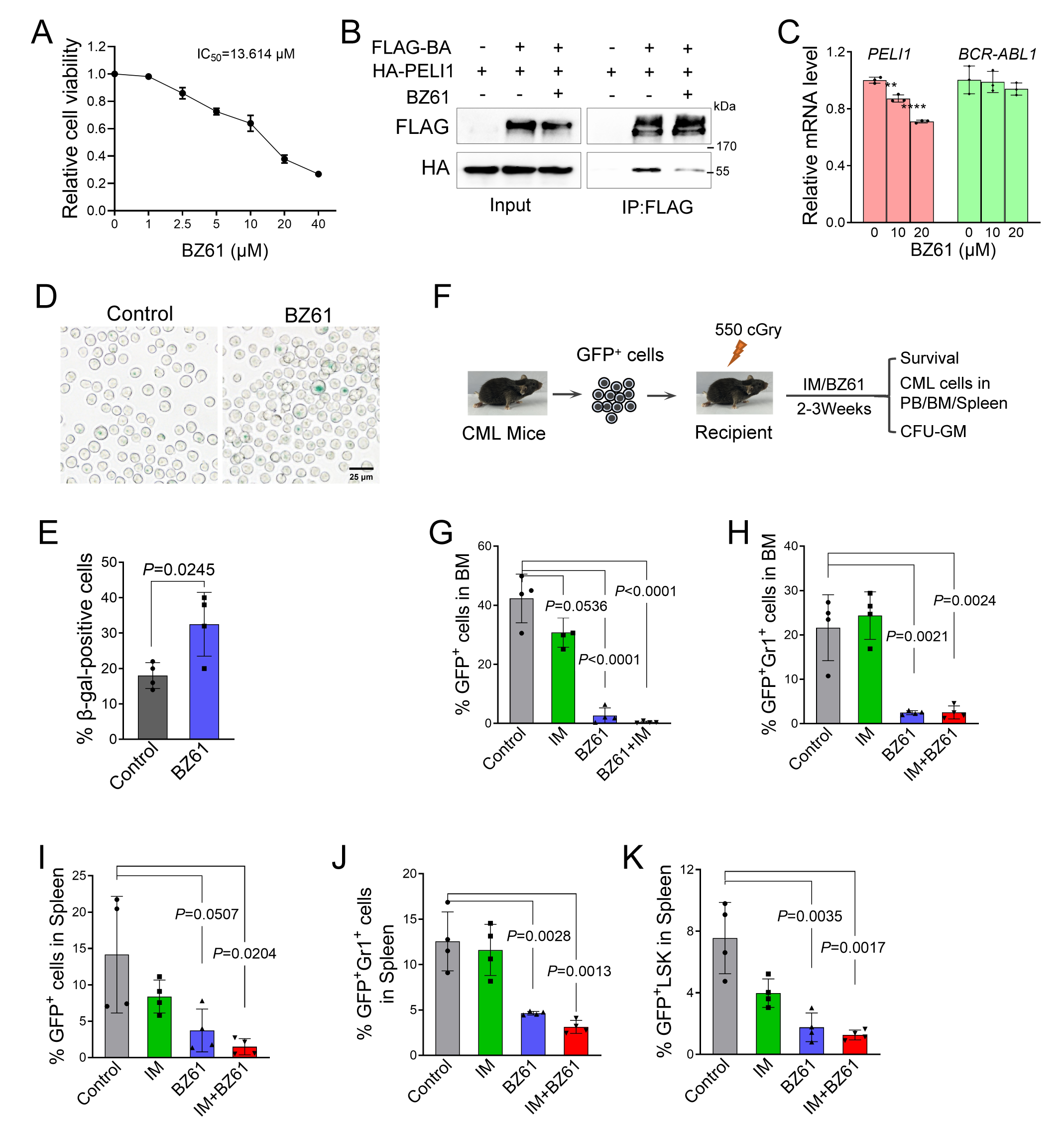

Supplement: Supplementary file 10 — Supplementary Figures 9 [file 41419_2026_8799_MOESM10_ESM.tif]

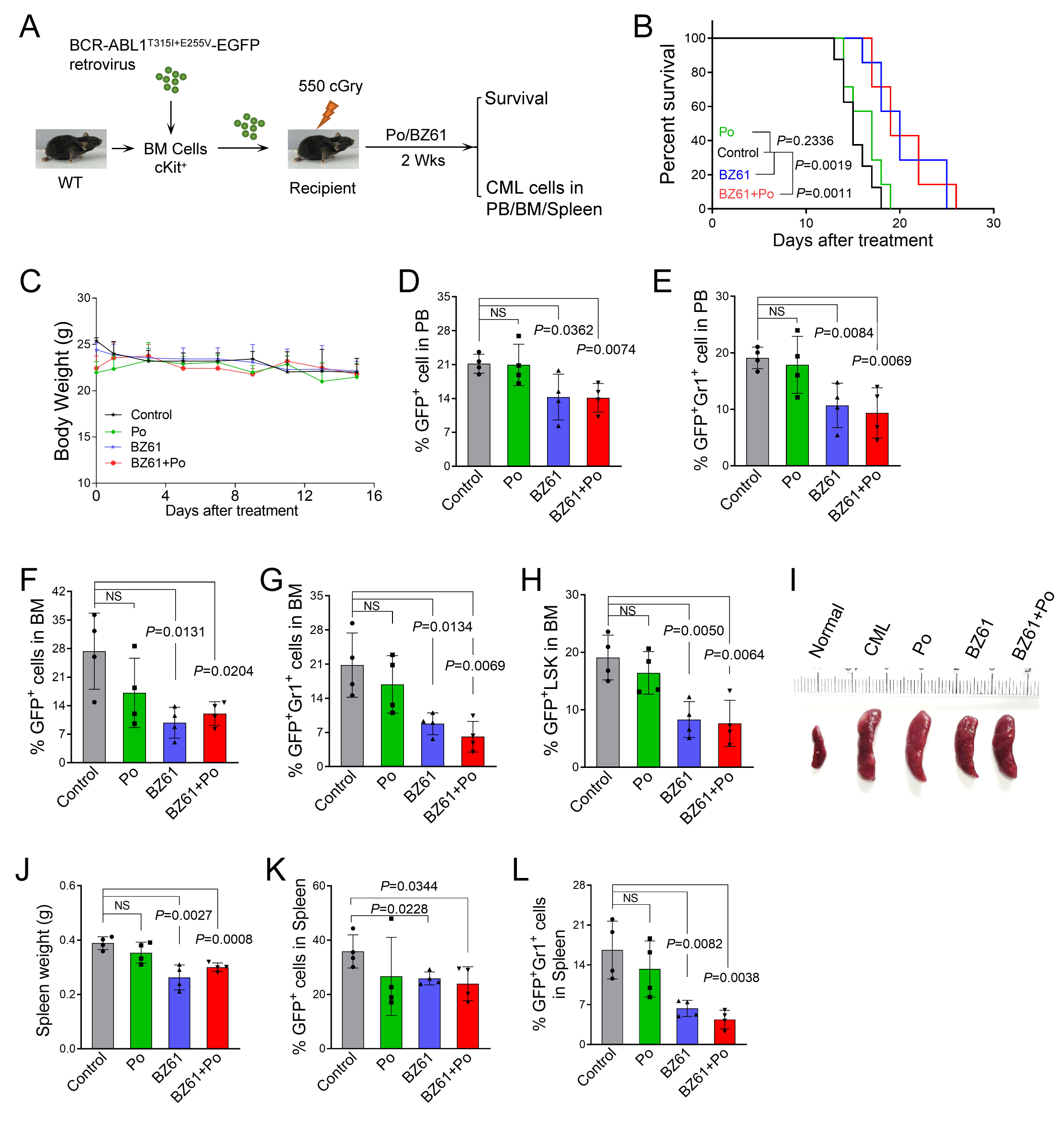

Supplement: Supplementary file 11 — Supplementary Figures 10 [file 41419_2026_8799_MOESM11_ESM.tif]

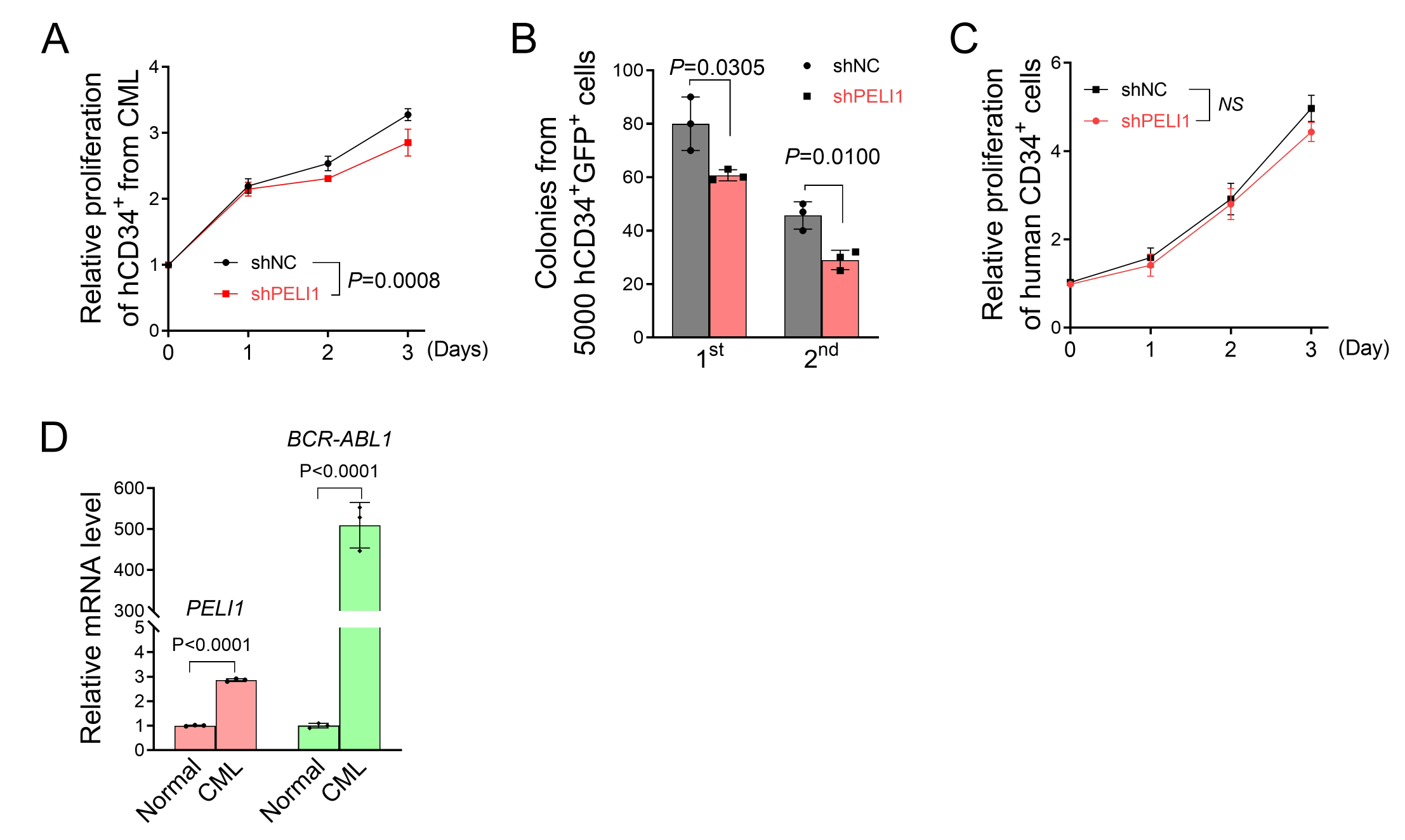

Supplement: Supplementary file 12 — Supplementary Figures 11 [file 41419_2026_8799_MOESM12_ESM.tif]
