# Supplementary material for: Inhibition of Pellino-1 reverts the progression and tyrosine kinase inhibitor resistance in chronic myeloid leukemia
Source: Cell Death Dis. 2026 May 5;17(1):593. doi: 10.1038/s41419-026-08799-7 (PMC13287773; doi:10.1038/s41419-026-08799-7)

Protein marker used in this study

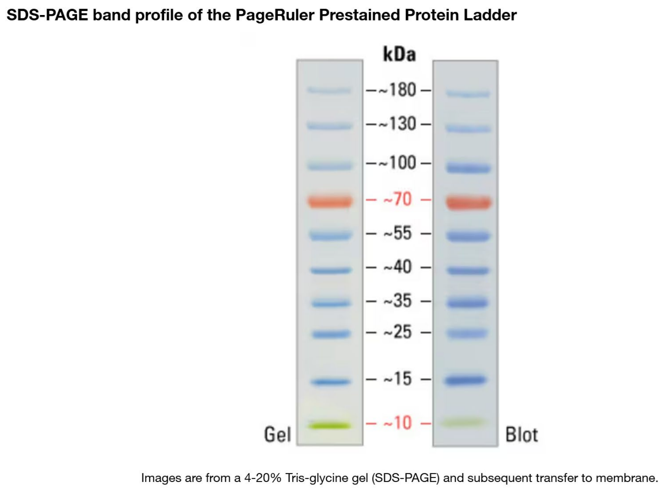

ThermoFisher, 26616

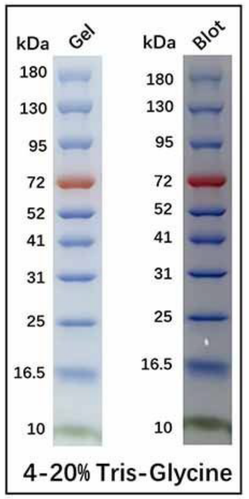

Aladdin, rp192282

Fig1A

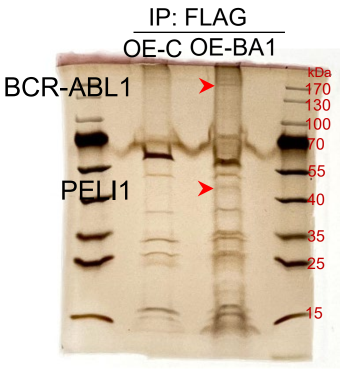

Fig1B

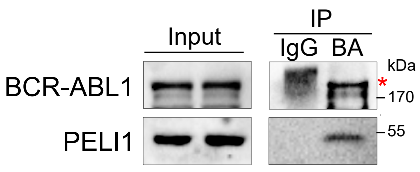

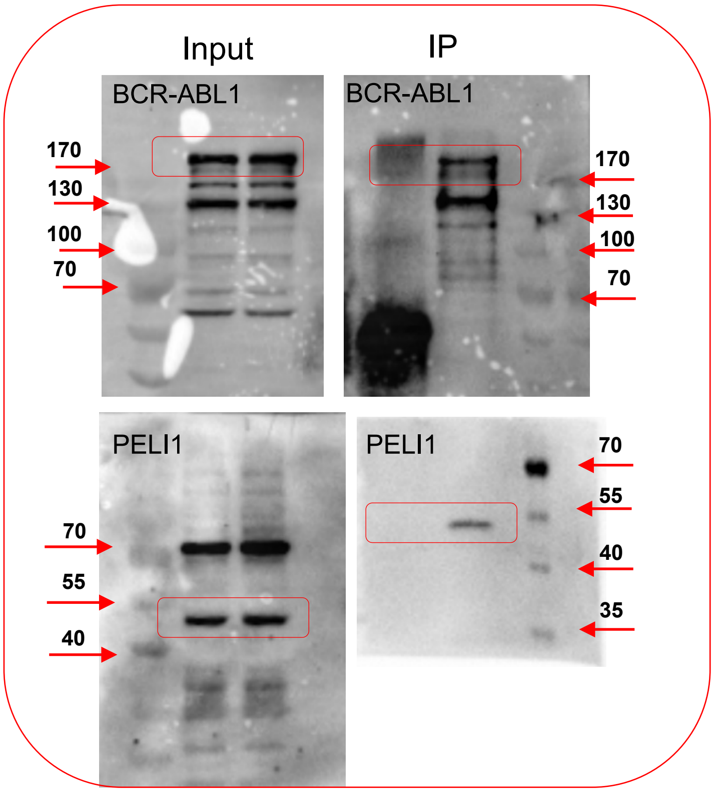

Fig1C

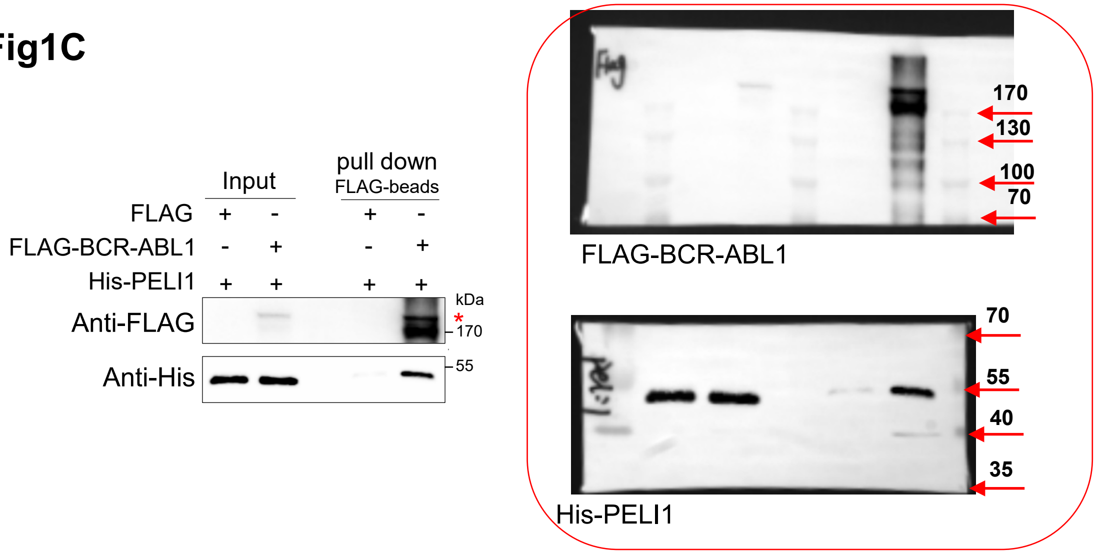

Fig1D

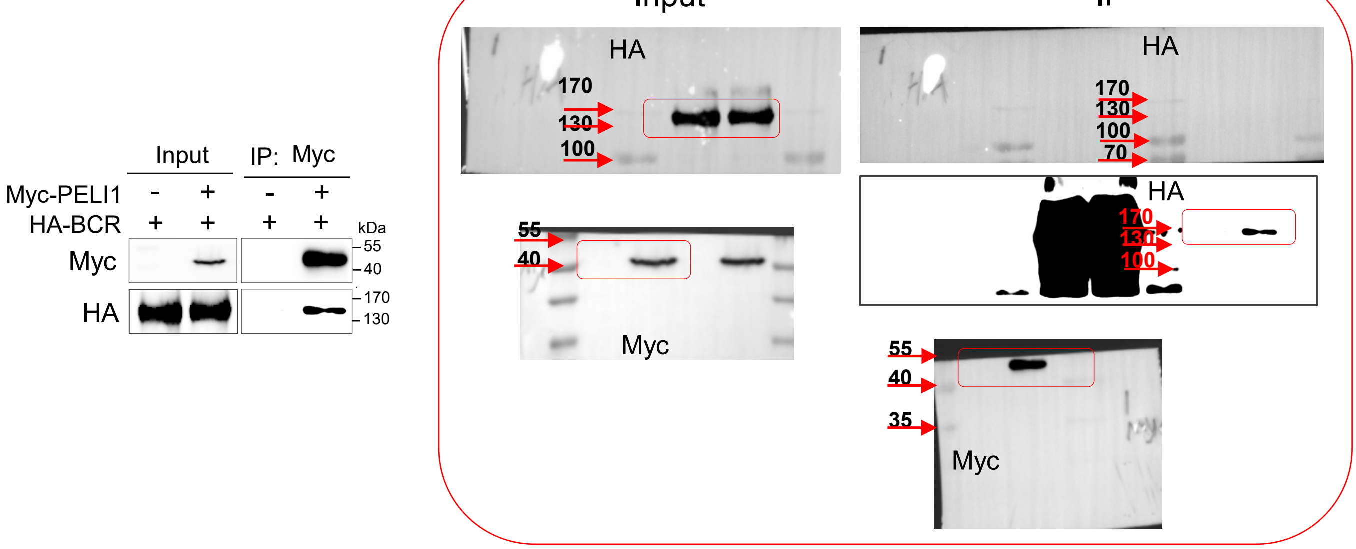

Fig1F

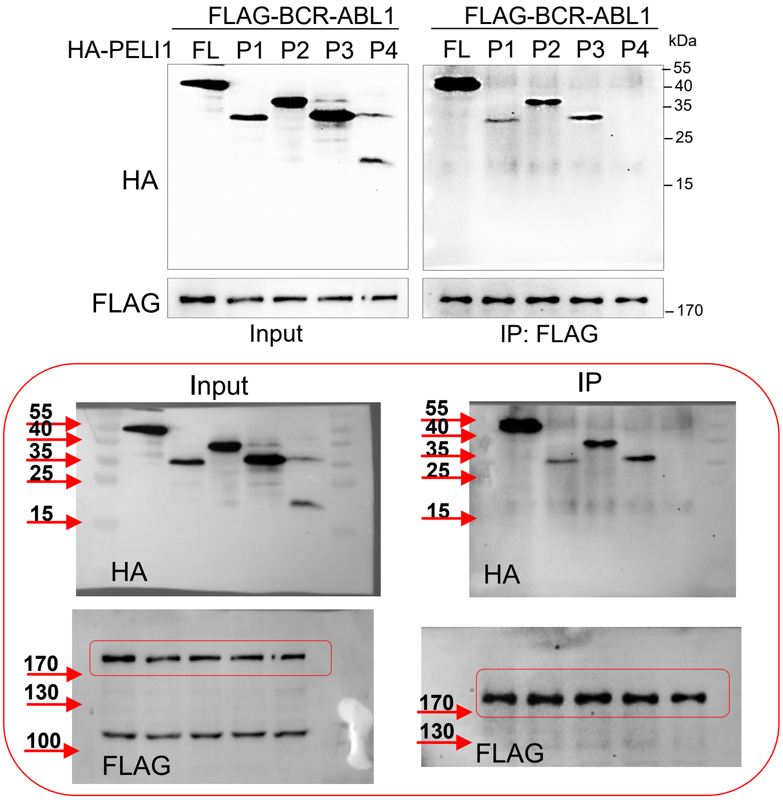

Fig1G

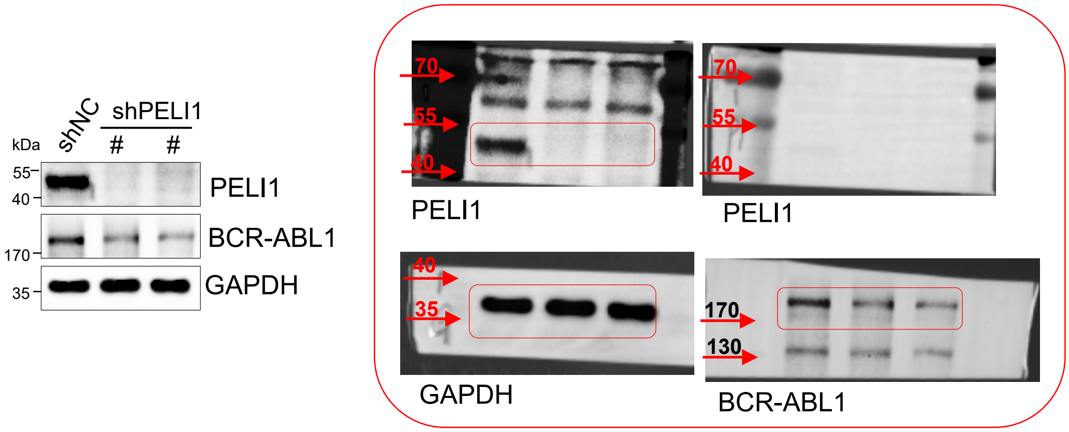

Fig1H

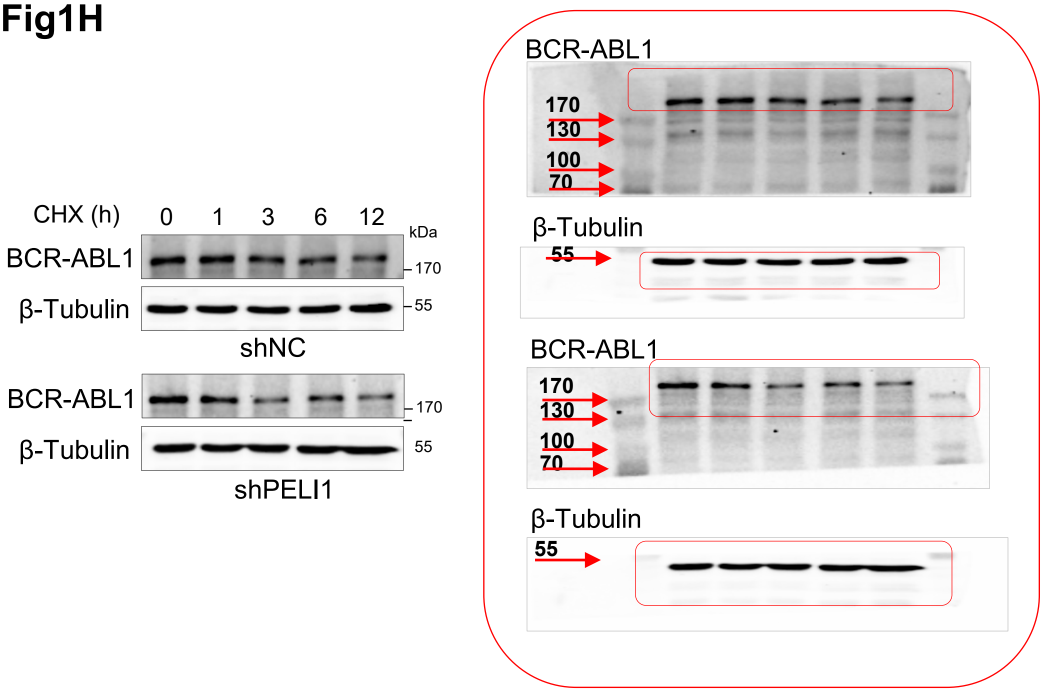

Fig1J

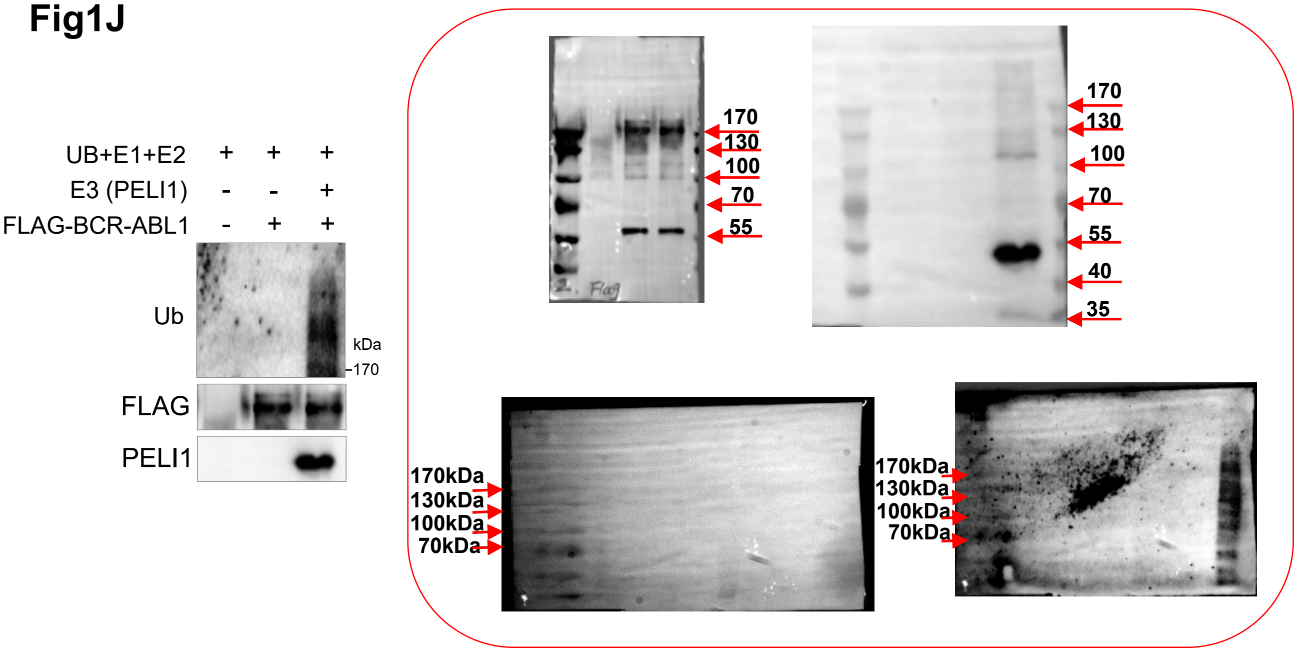

Fig1K

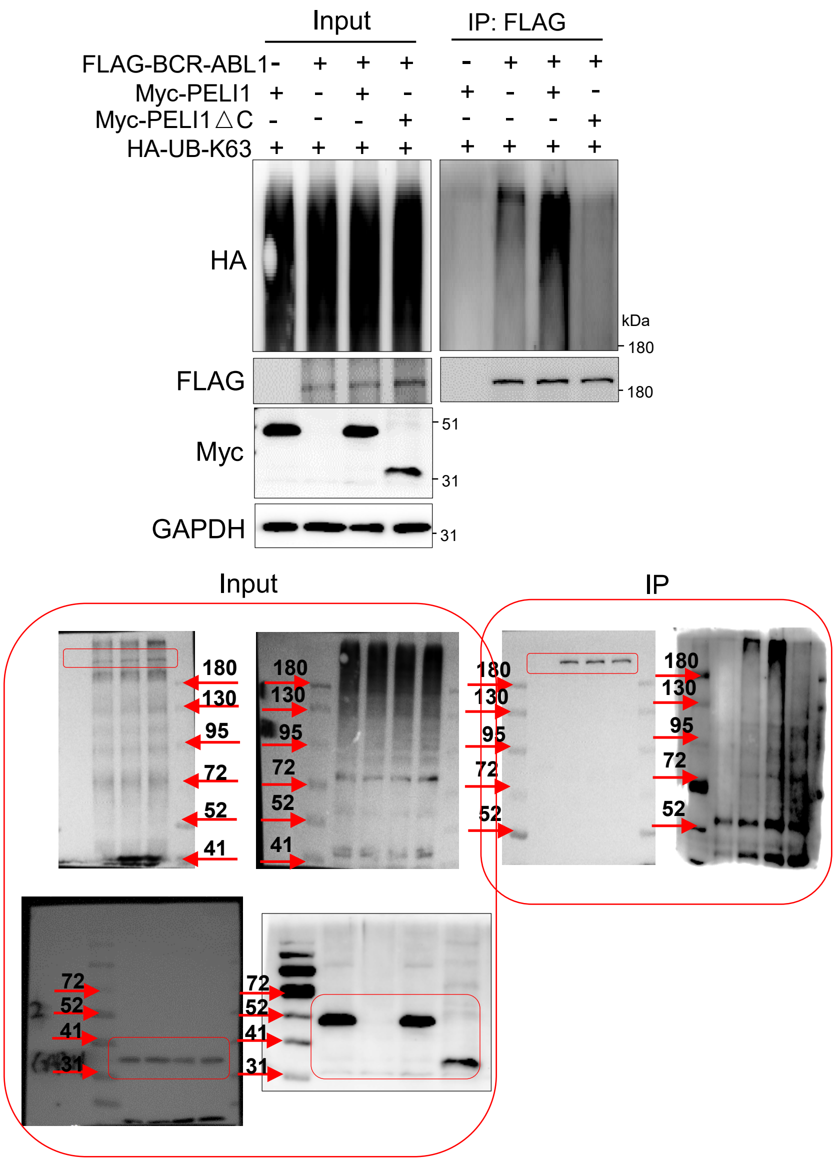

Input
IP: FLAG
FLAG-BCR-ABL1
Myc-PELI1
Myc-PELI1△C
HA-UB-K63
HA
FLAG
Myc
GAPDH
kDa
180
51
31
Input
IP
180
130
95
72
52
41
31

Fig1L

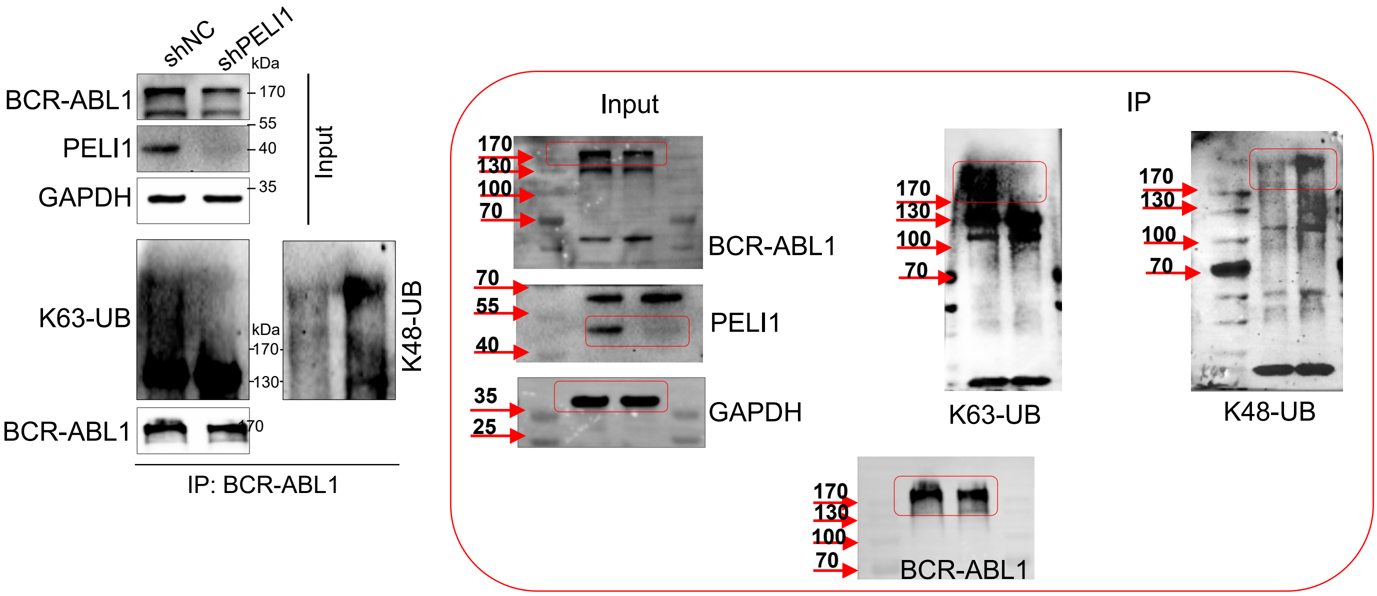

shNC
shPELI1
kDa
BCR-ABL1
PELI1
GAPDH
Input
170
55
40
35
K63-UB
K48-UB
130
IP: BCR-ABL1
Input
IP
100
70
25

Fig2B

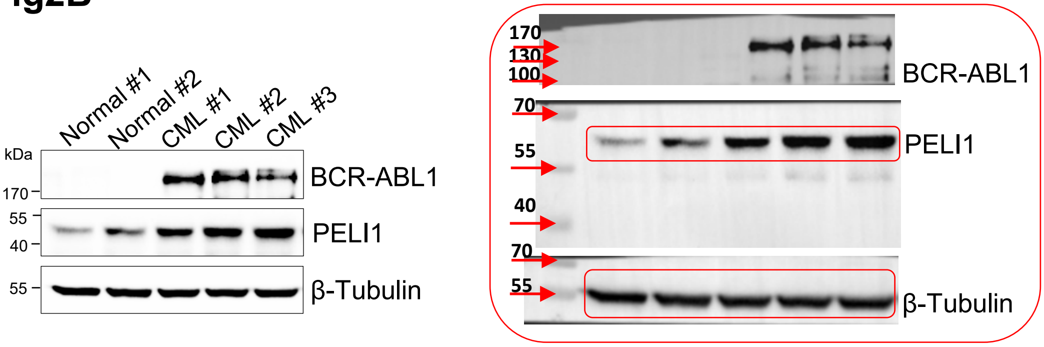

Fig2C

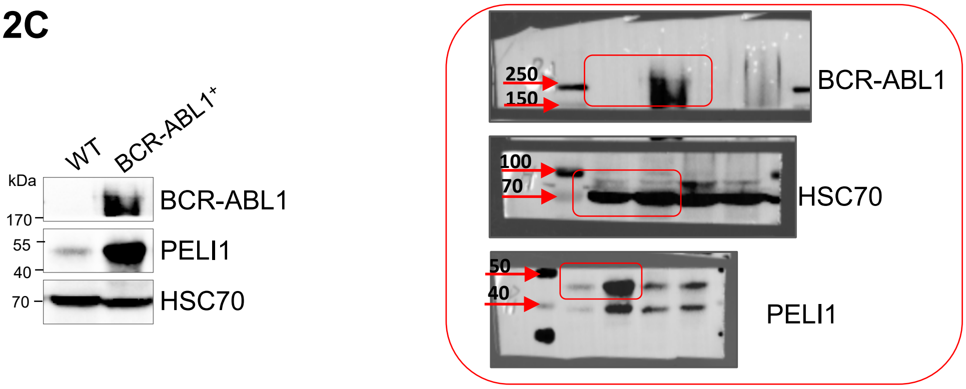

Fig2E

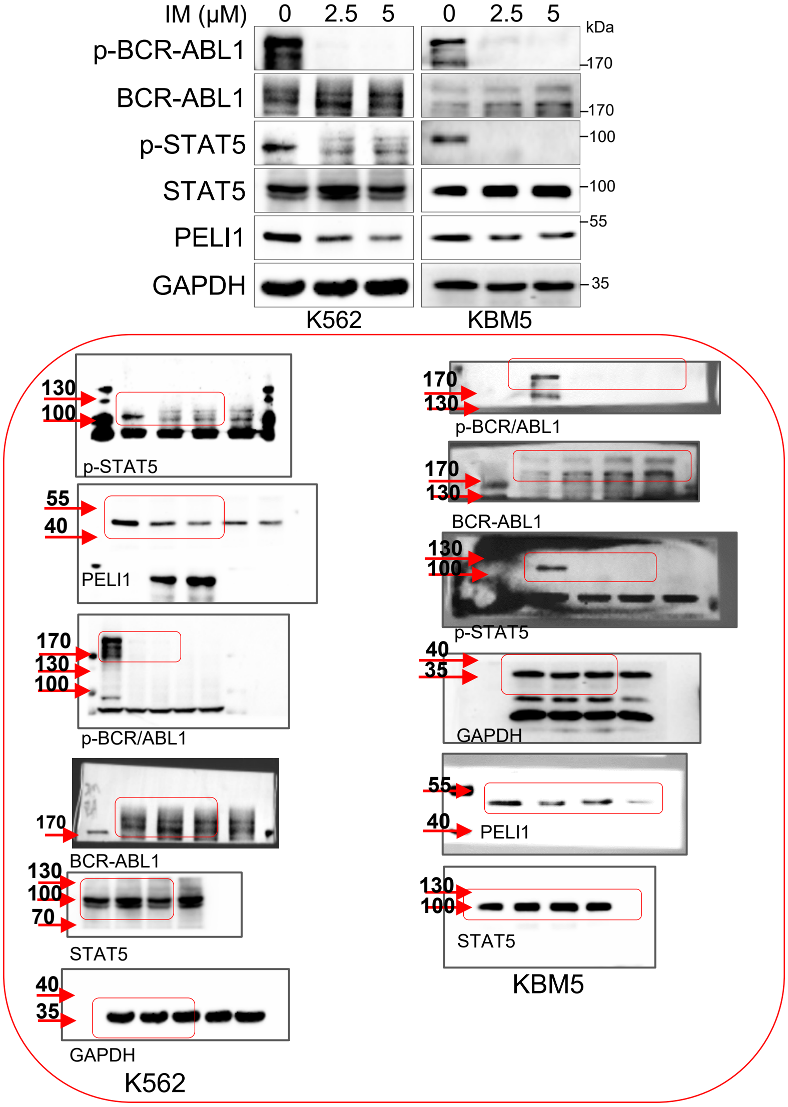

Fig2G

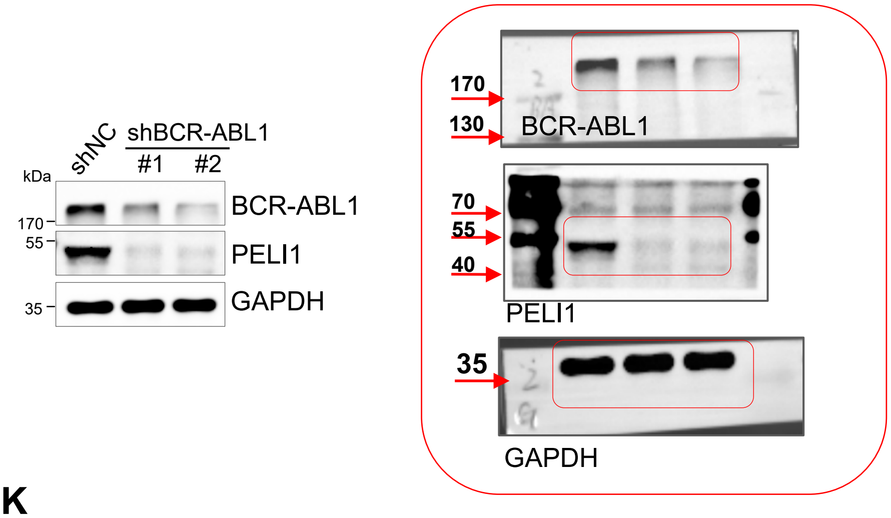

Fig2K

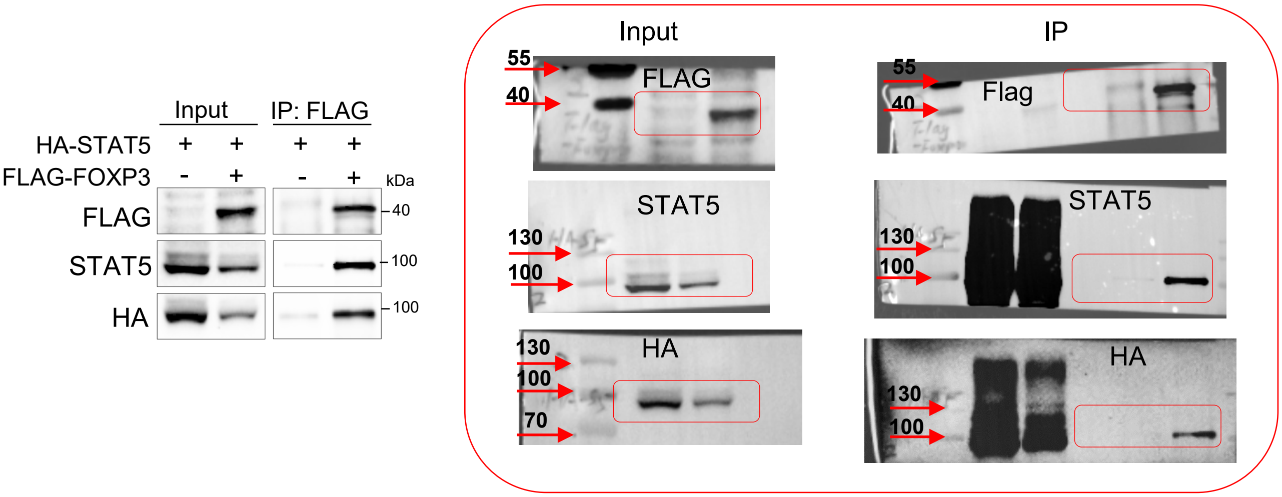

Fig3G

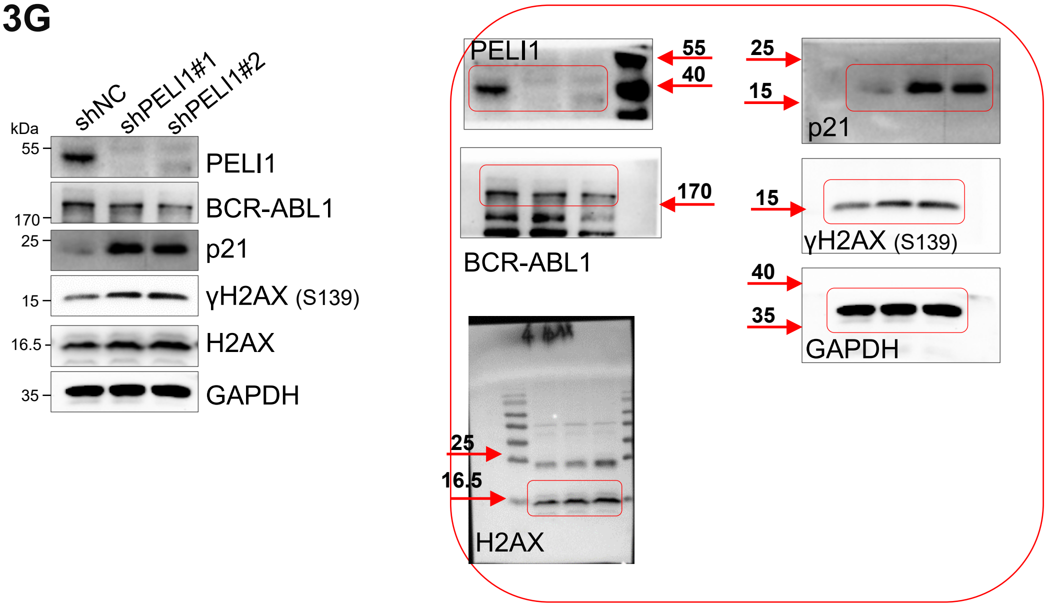

Fig3H

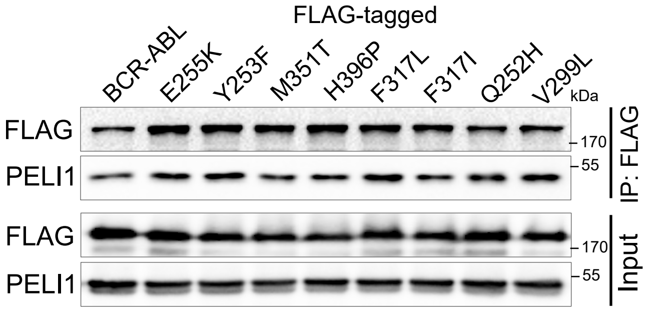

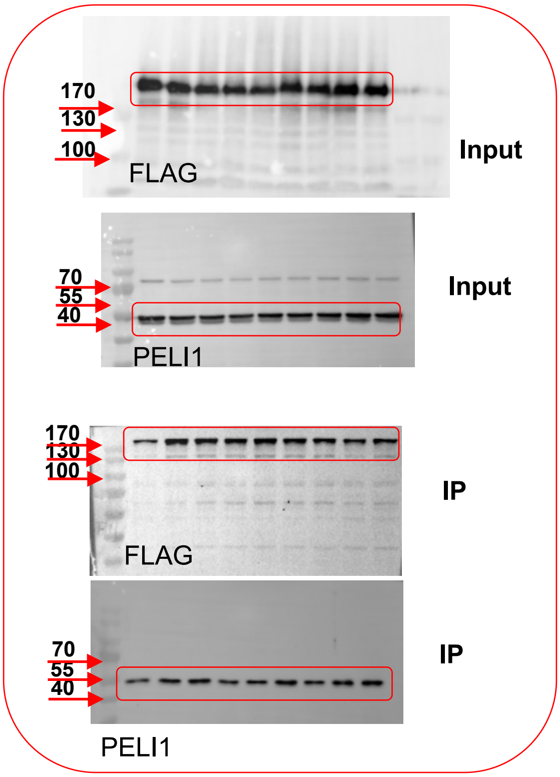

Fig3I

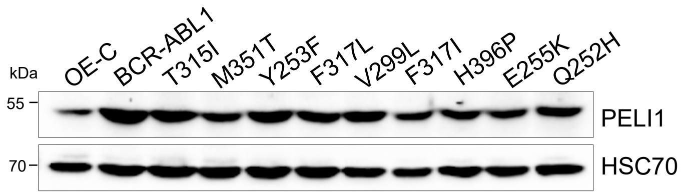

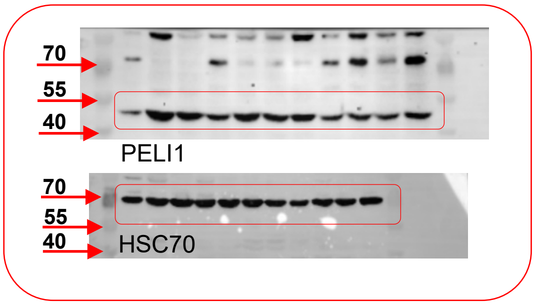

Fig3J

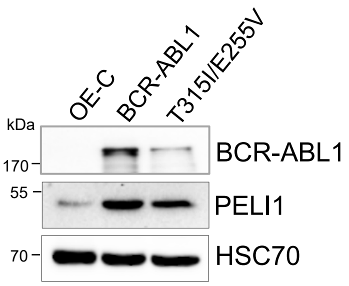

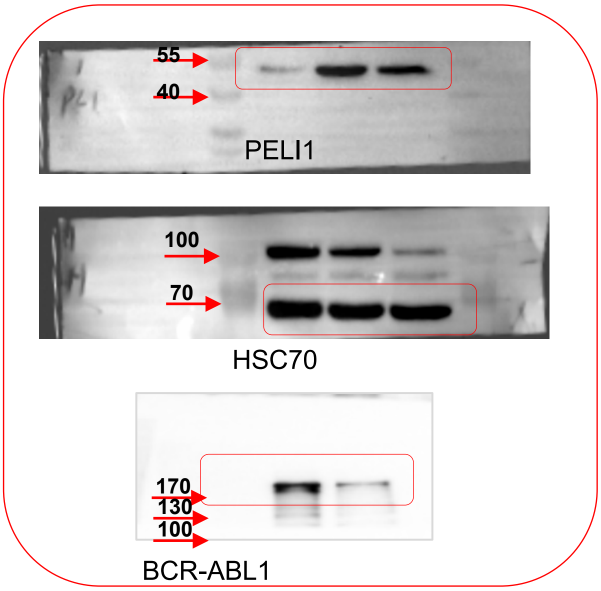

Fig 4L

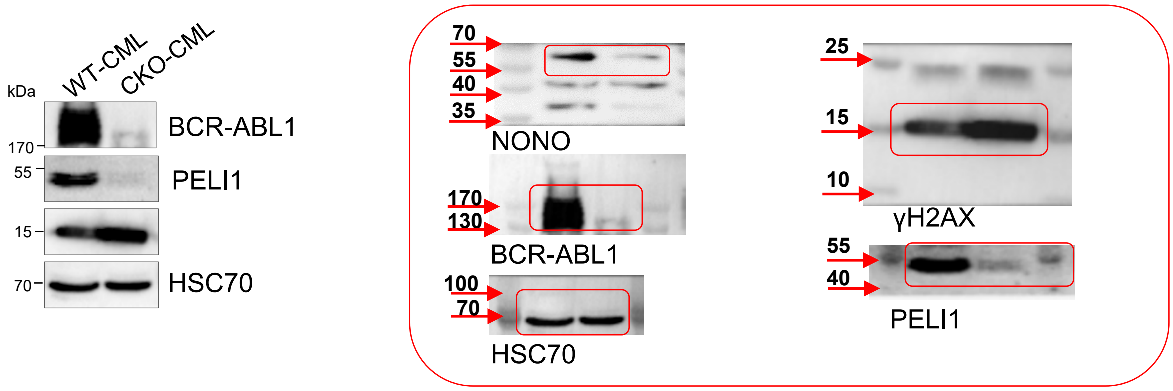

Fig 5D

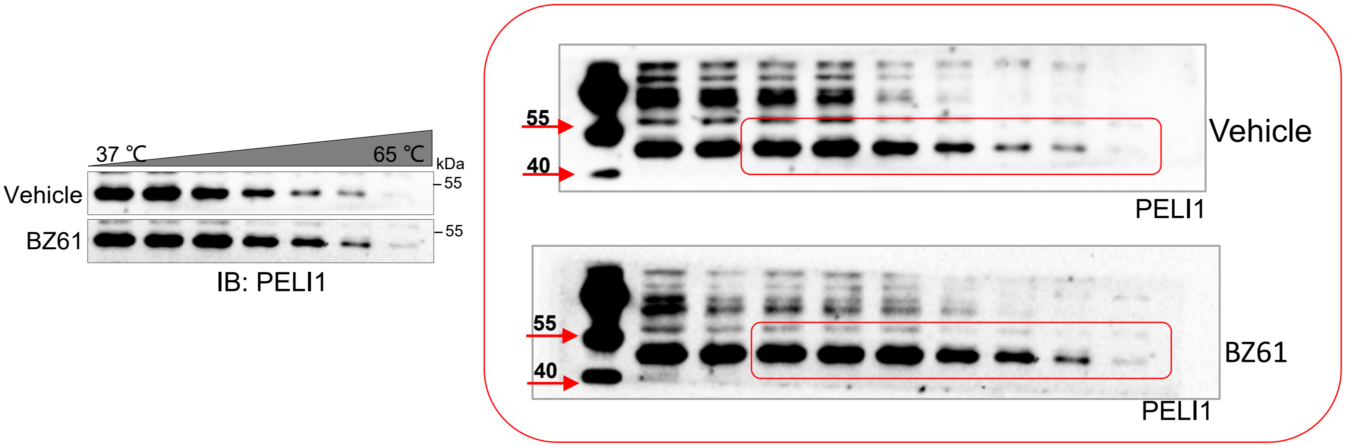

Fig 5F

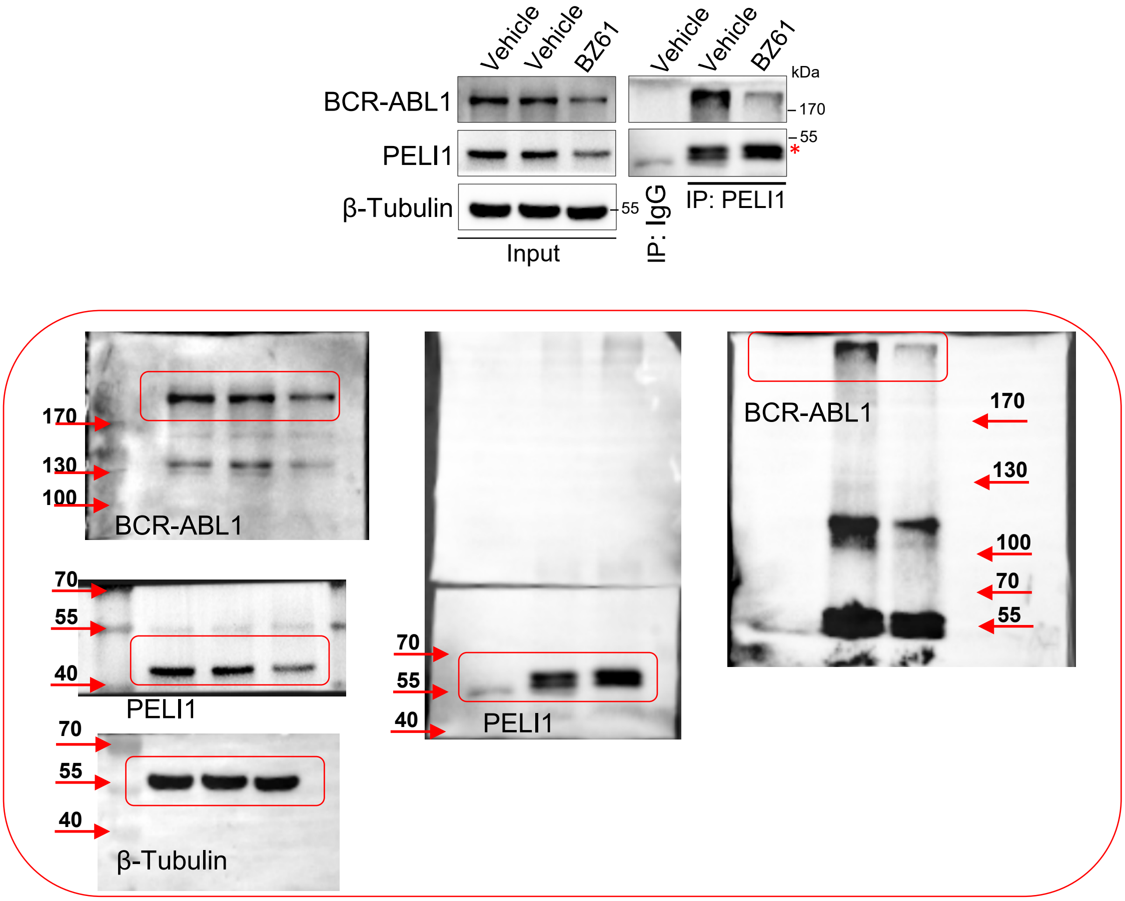

Fig 5G

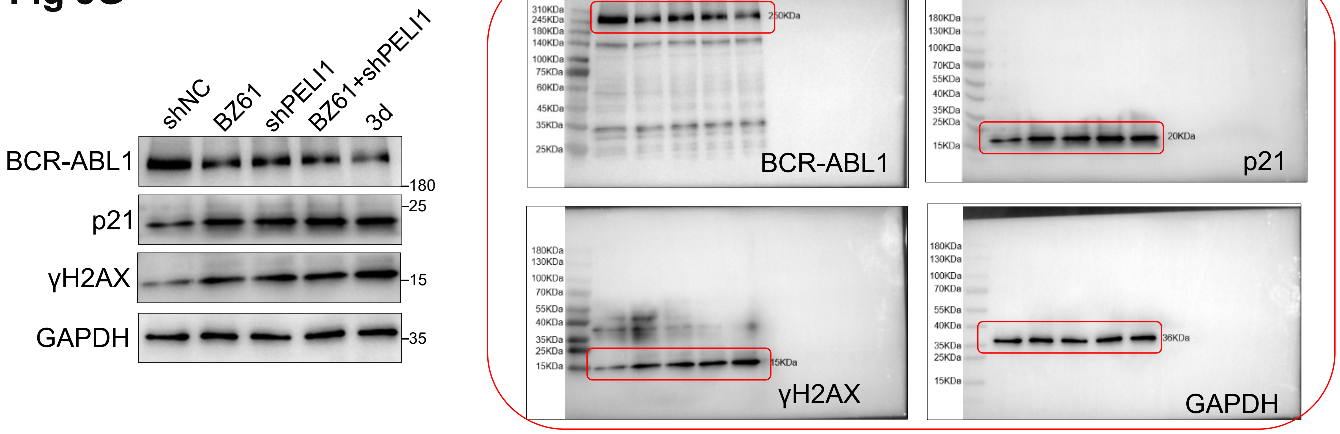

Fig 5H

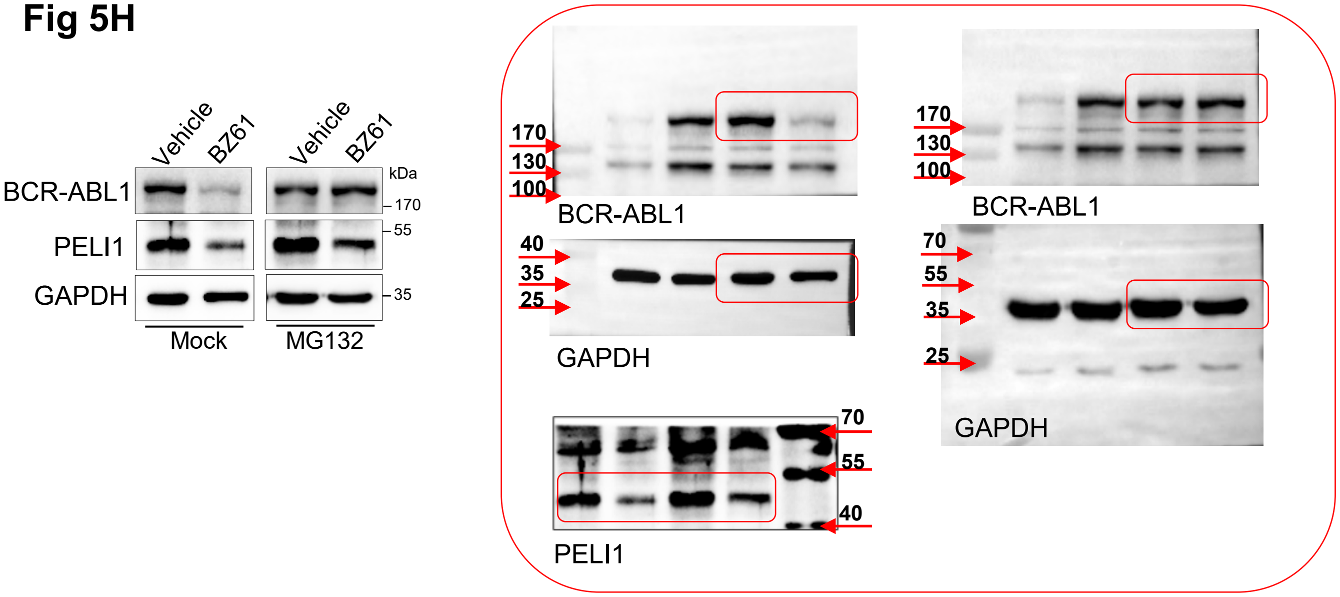

Fig 6C

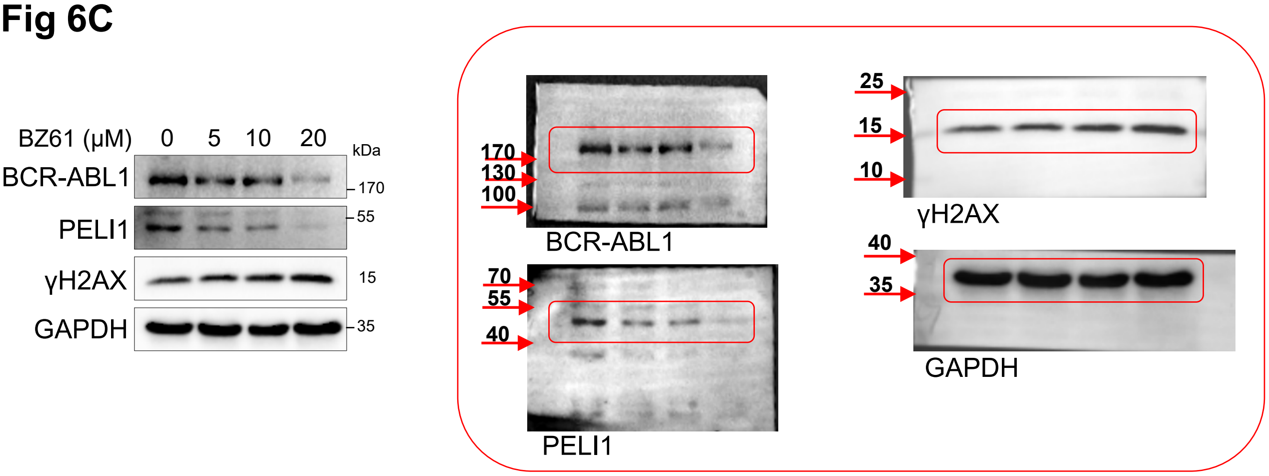

Suppl Fig.1B

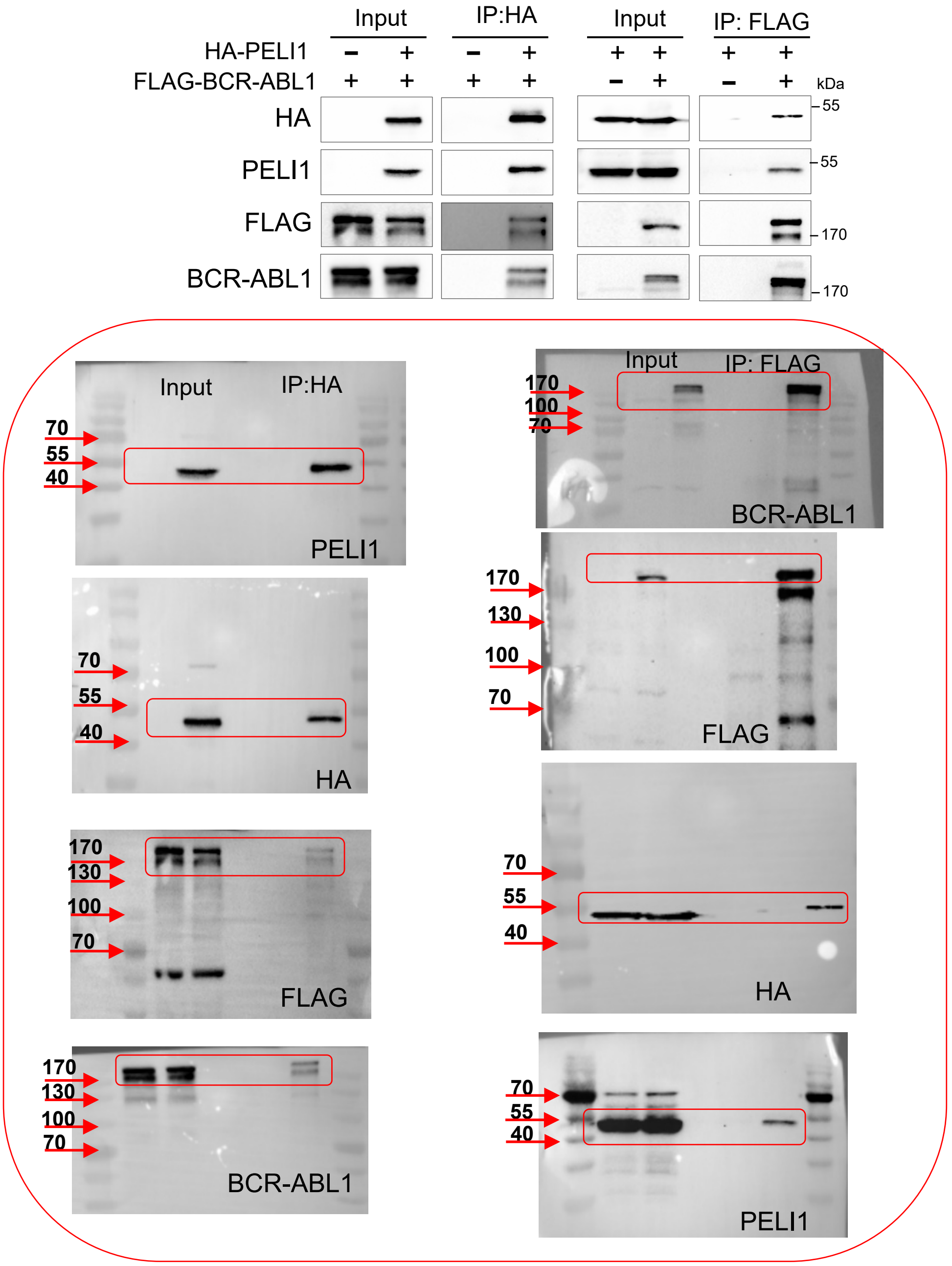

Suppl Fig.1C

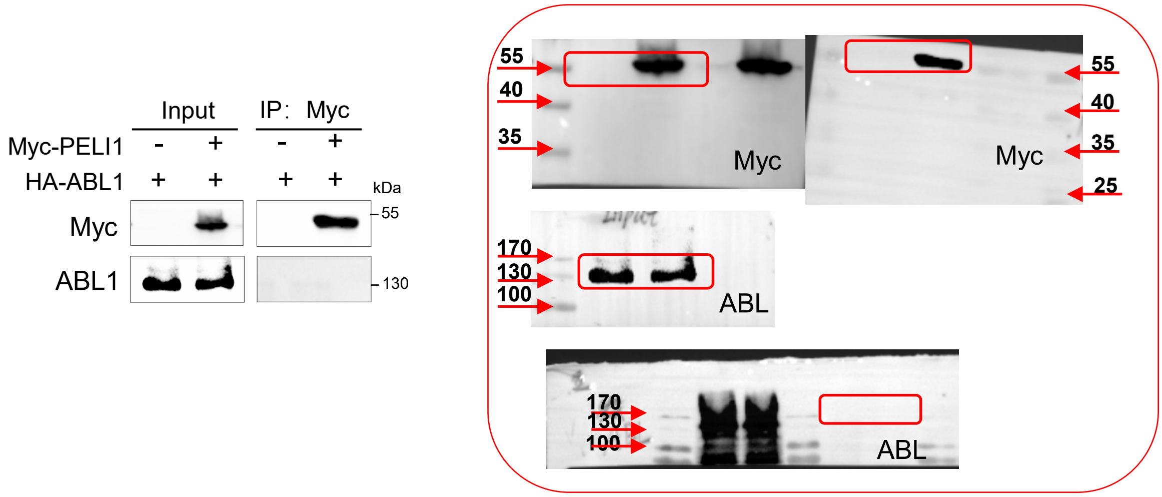

Suppl Fig.1E

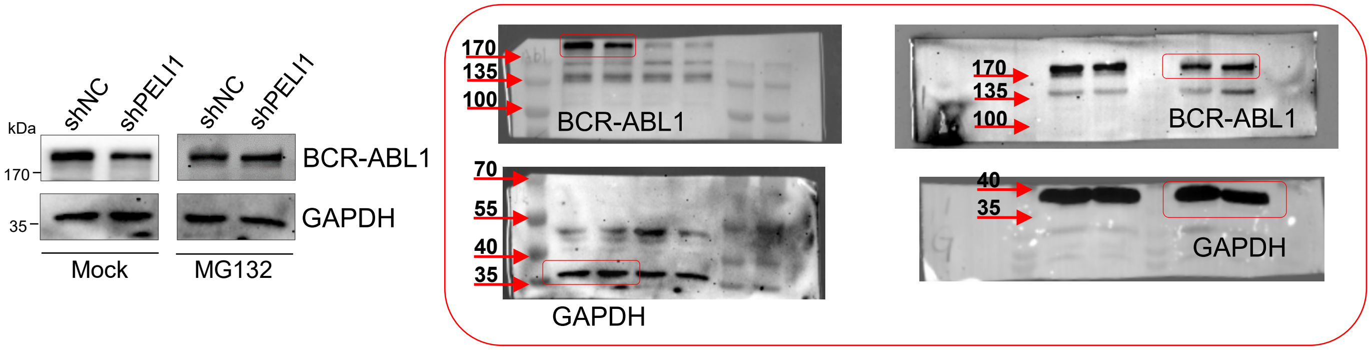

Suppl Fig.1F

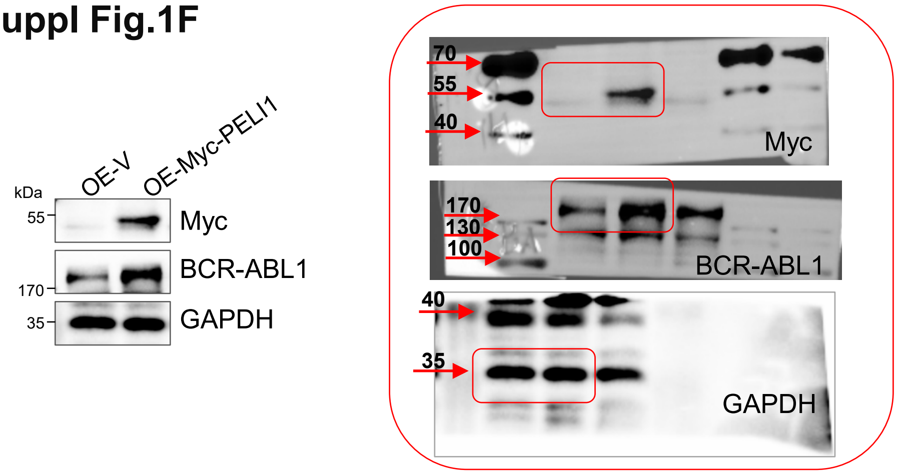

Suppl Fig.1H

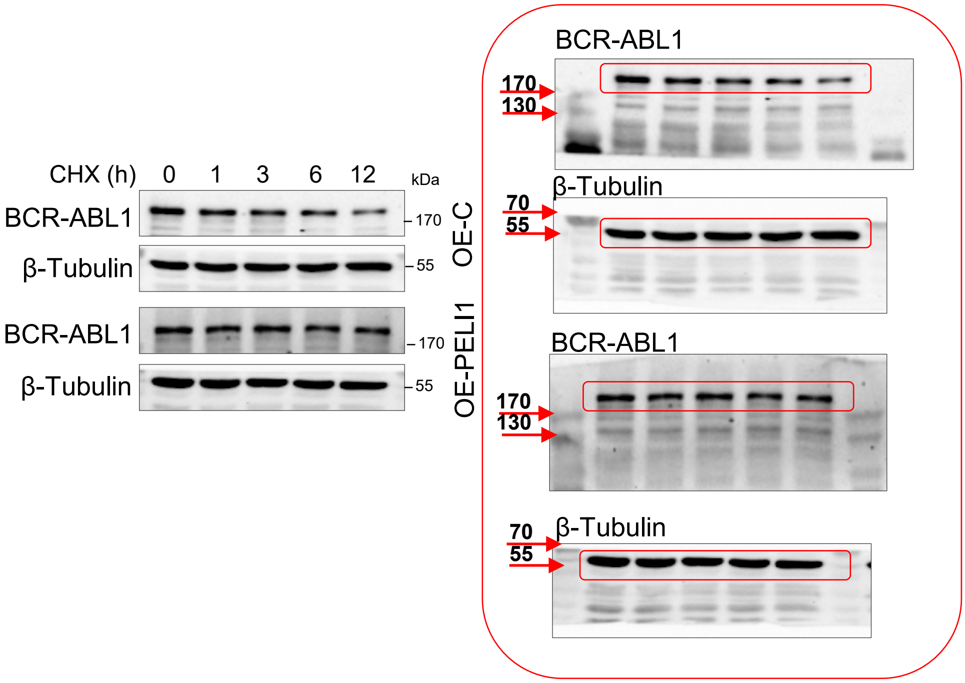

Suppl Fig.1J

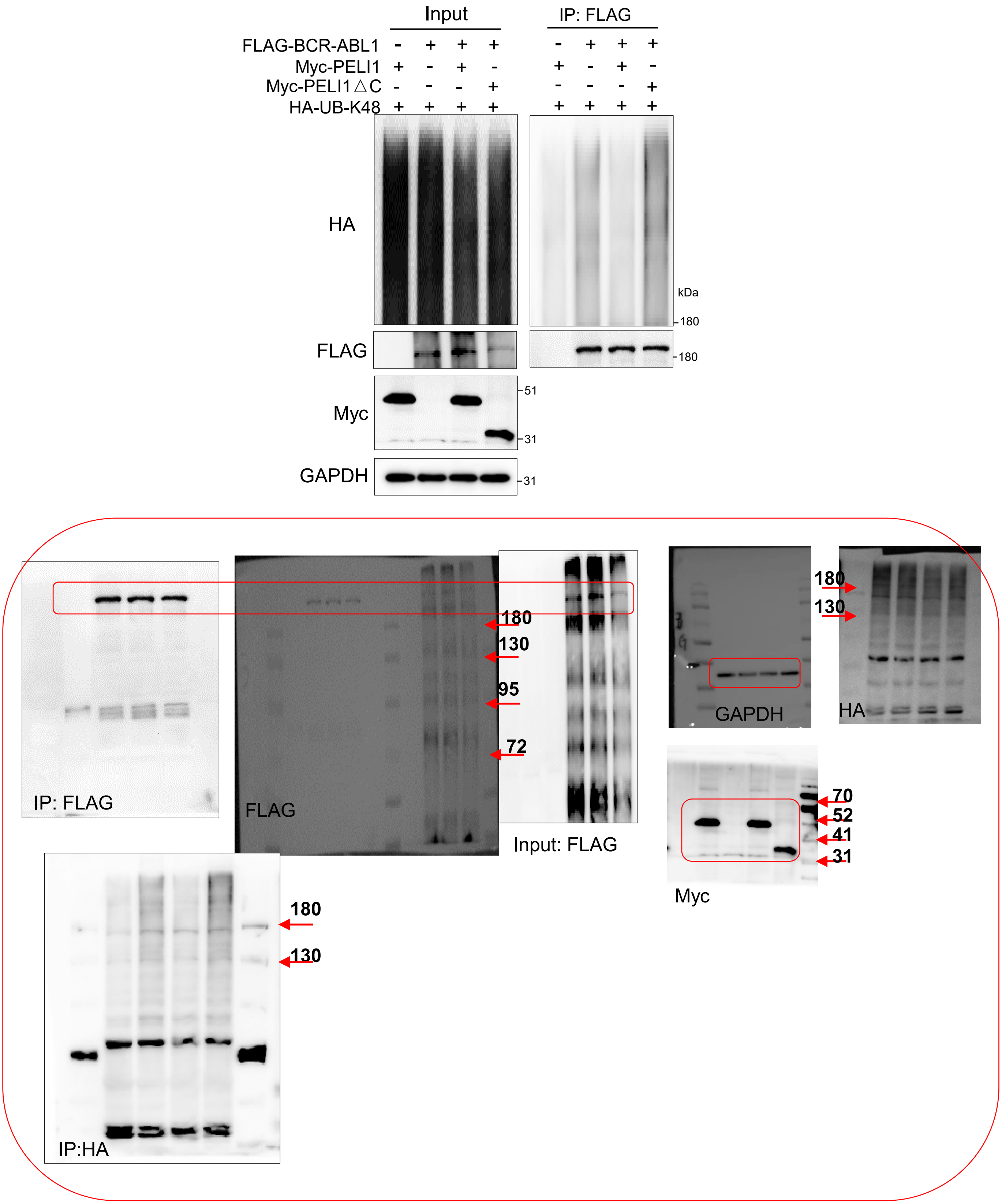

Input
IP: FLAG
FLAG-BCR-ABL1 - + + + - + + +
Myc-PELI1 + - + - + - + -
Myc-PELI1△C - - - + - - - +
HA-UB-K48 + + + + + + + +
HA
FLAG
Myc
GAPDH
kDa
180
180
51
31
31
IP: FLAG
FLAG
180
130
95
72
Input: FLAG
GAPDH
180
130
HA
70
52
41
31
Myc
IP:HA
180
130

Suppl Fig.2B

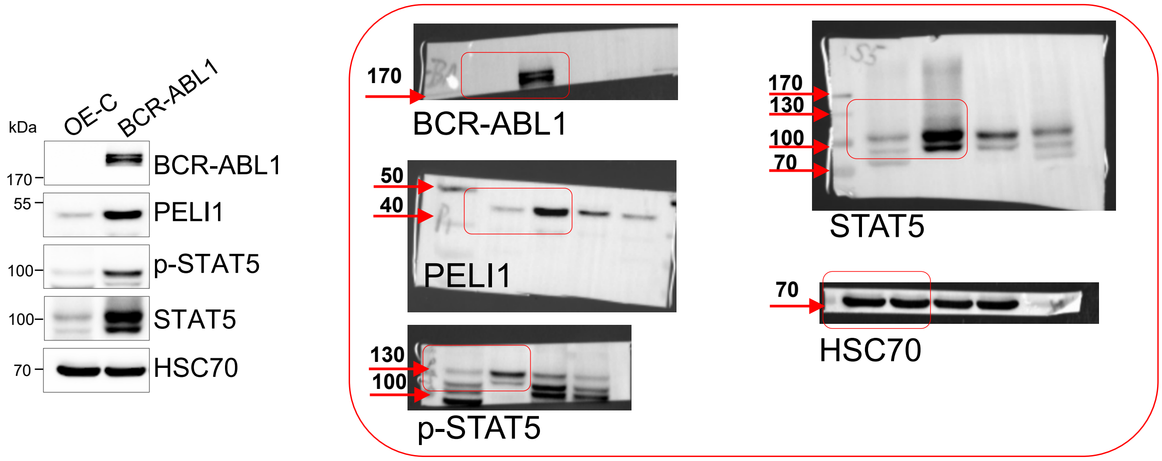

Suppl Fig.2D

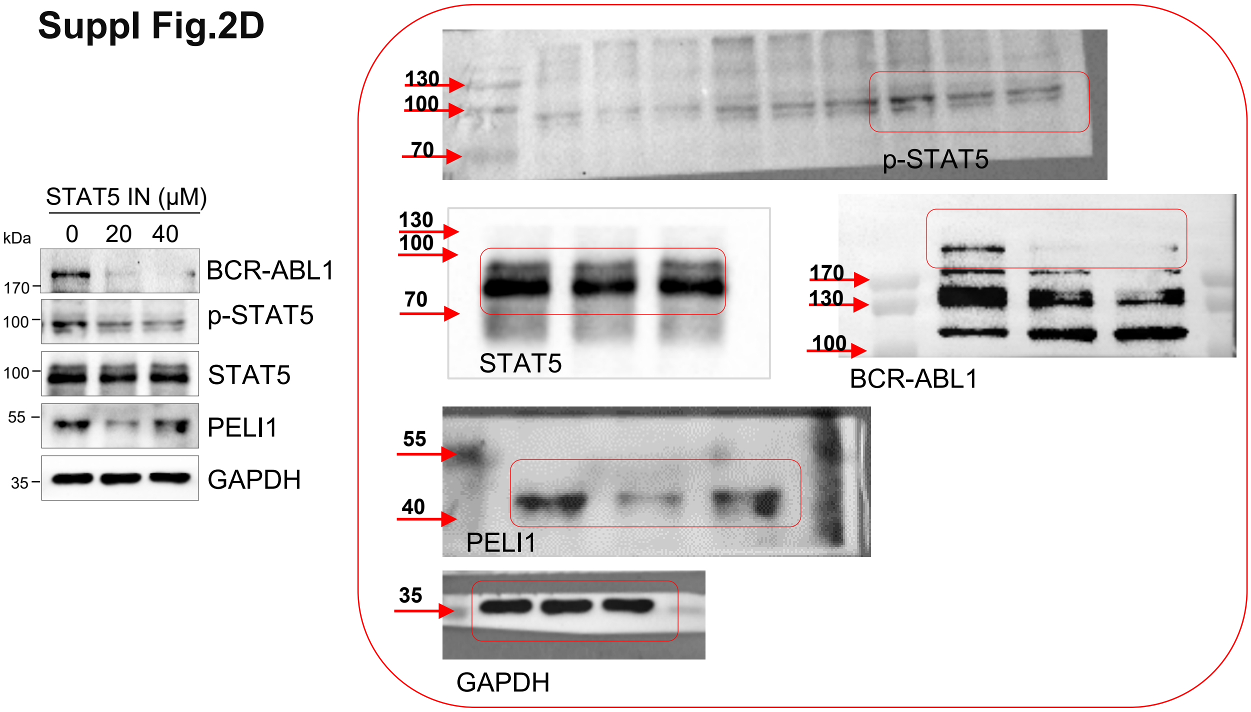

Suppl Fig.2J

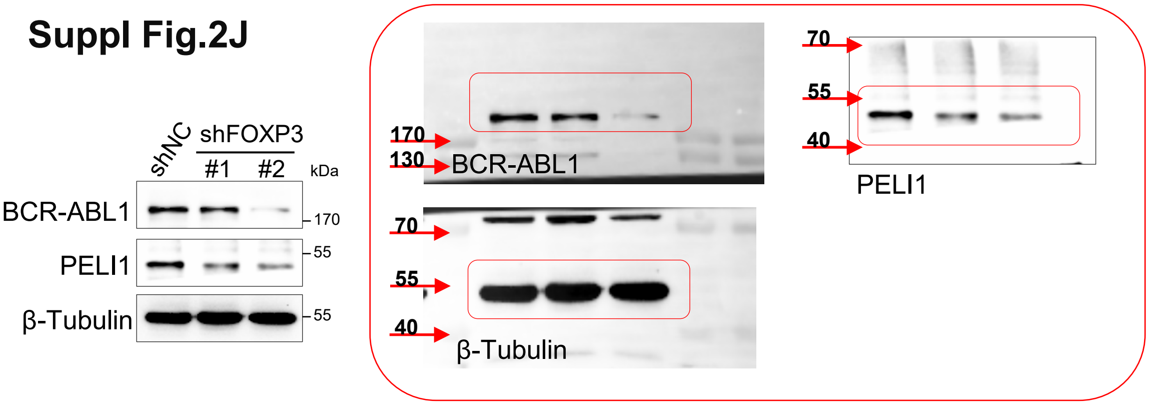

Suppl Fig. 3B

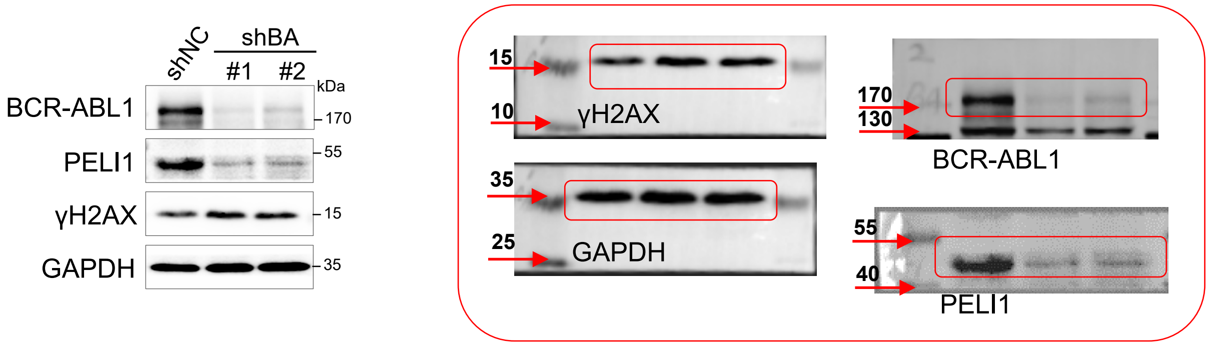

Suppl Fig. 3D

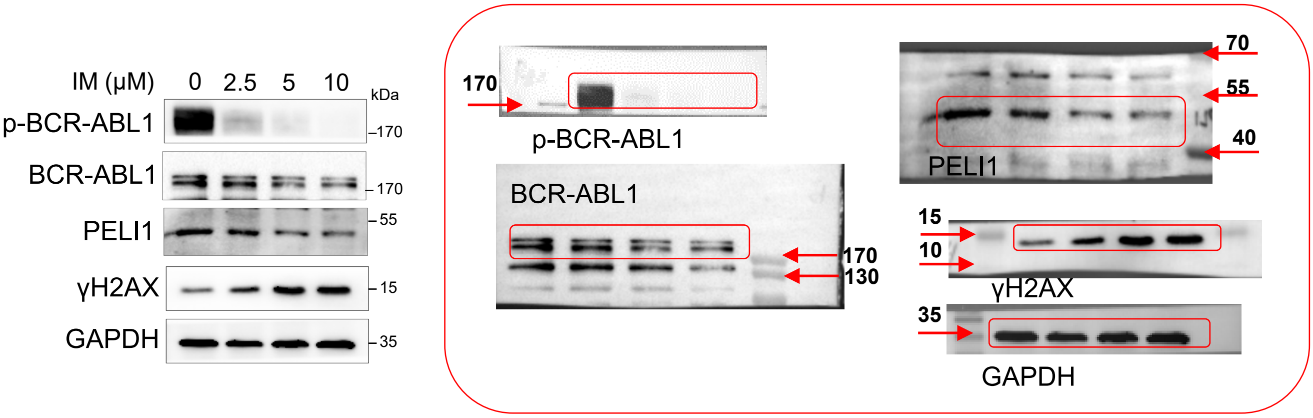

Suppl Fig.4A

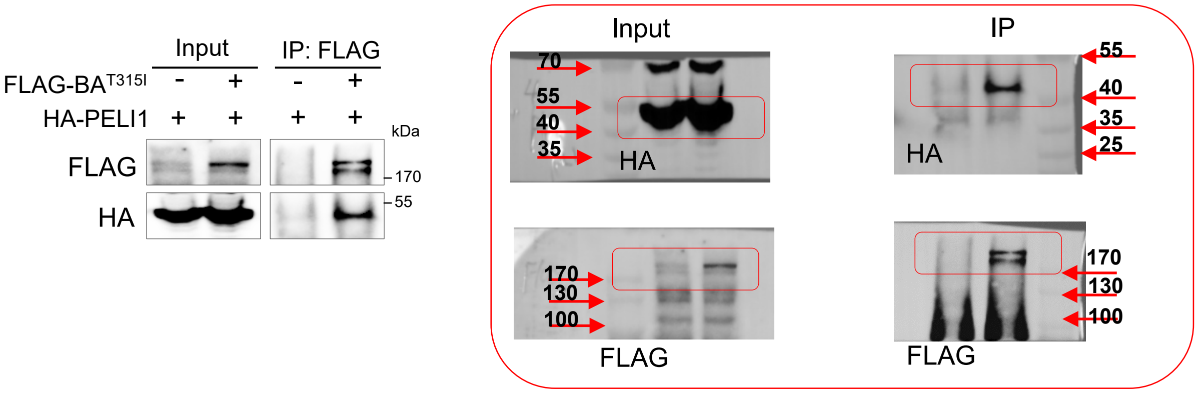

Suppl Fig.4C

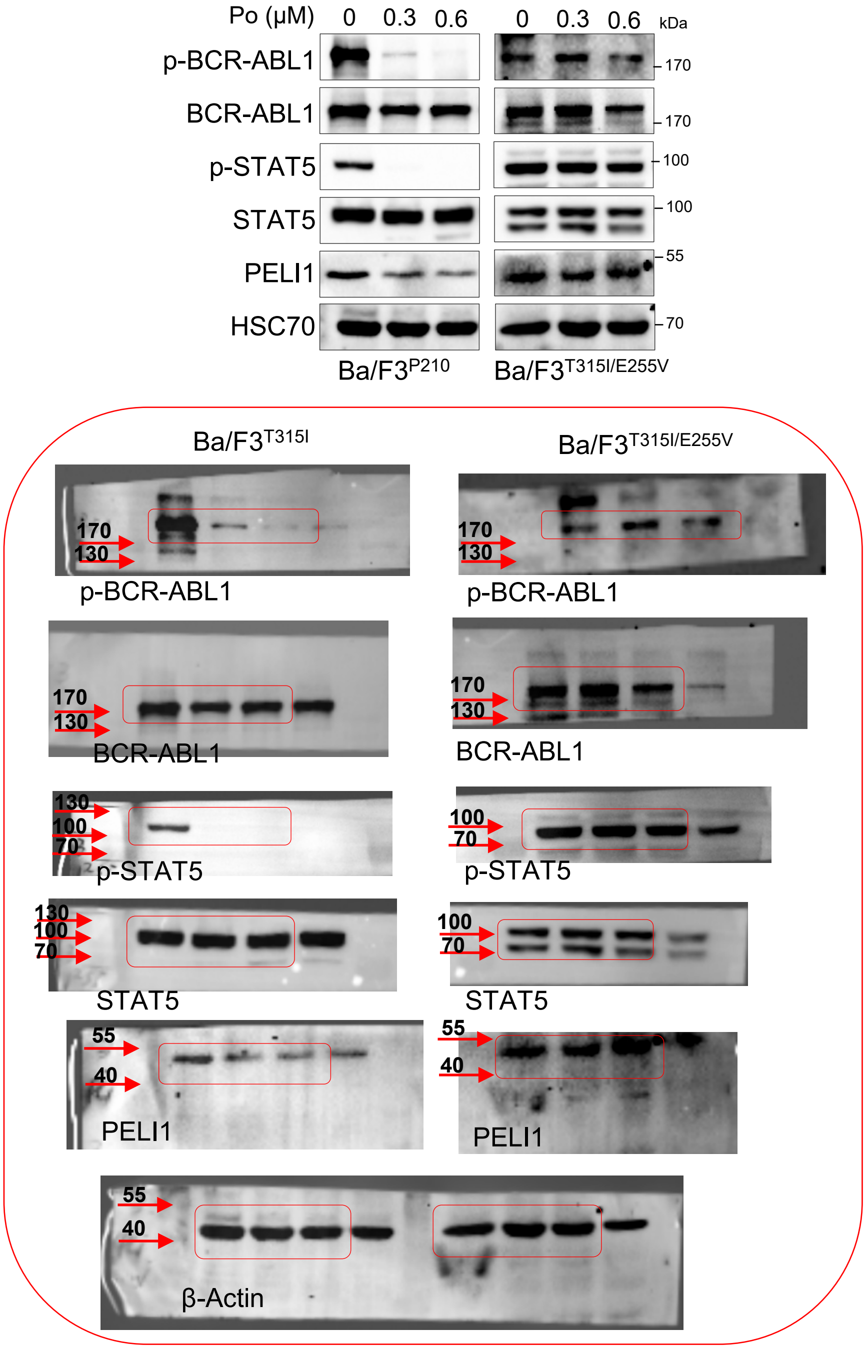

Suppl Fig.5B

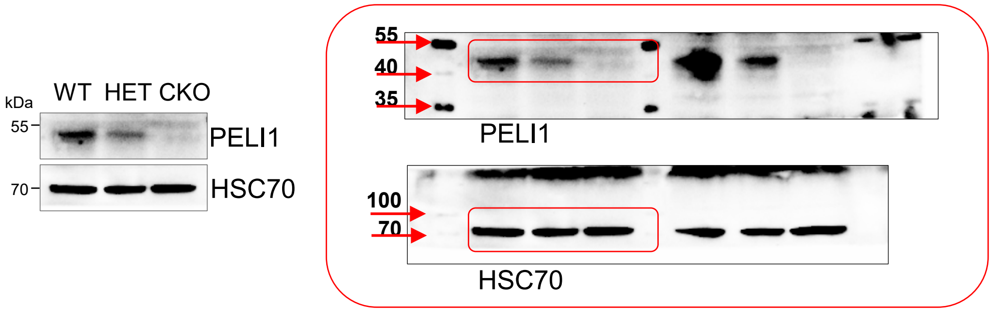

Suppl Fig.8C

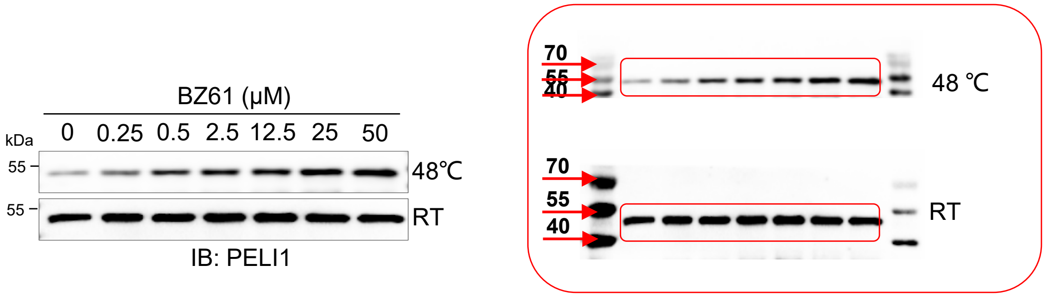

Suppl Fig.8D

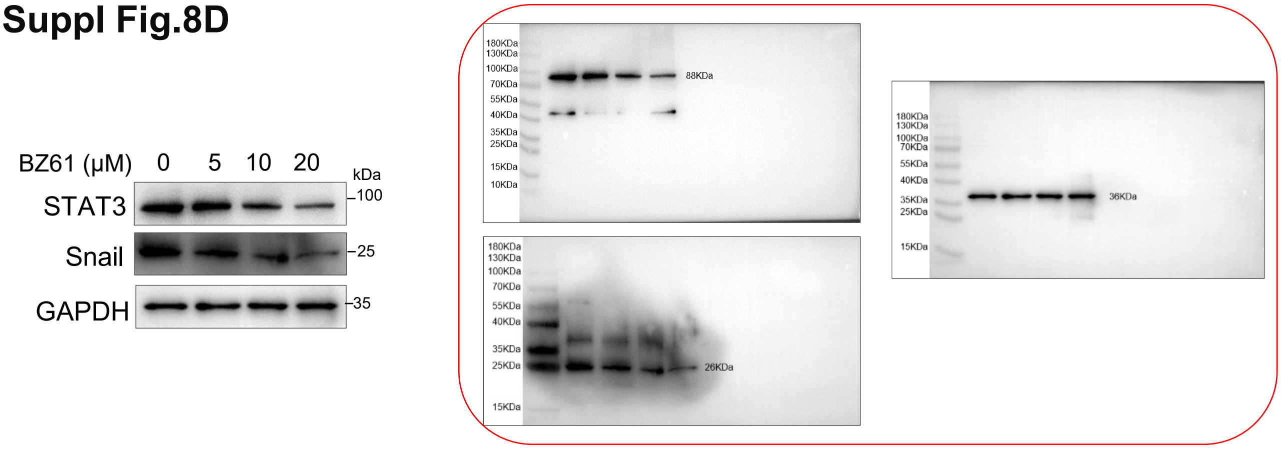

Suppl Fig.8E

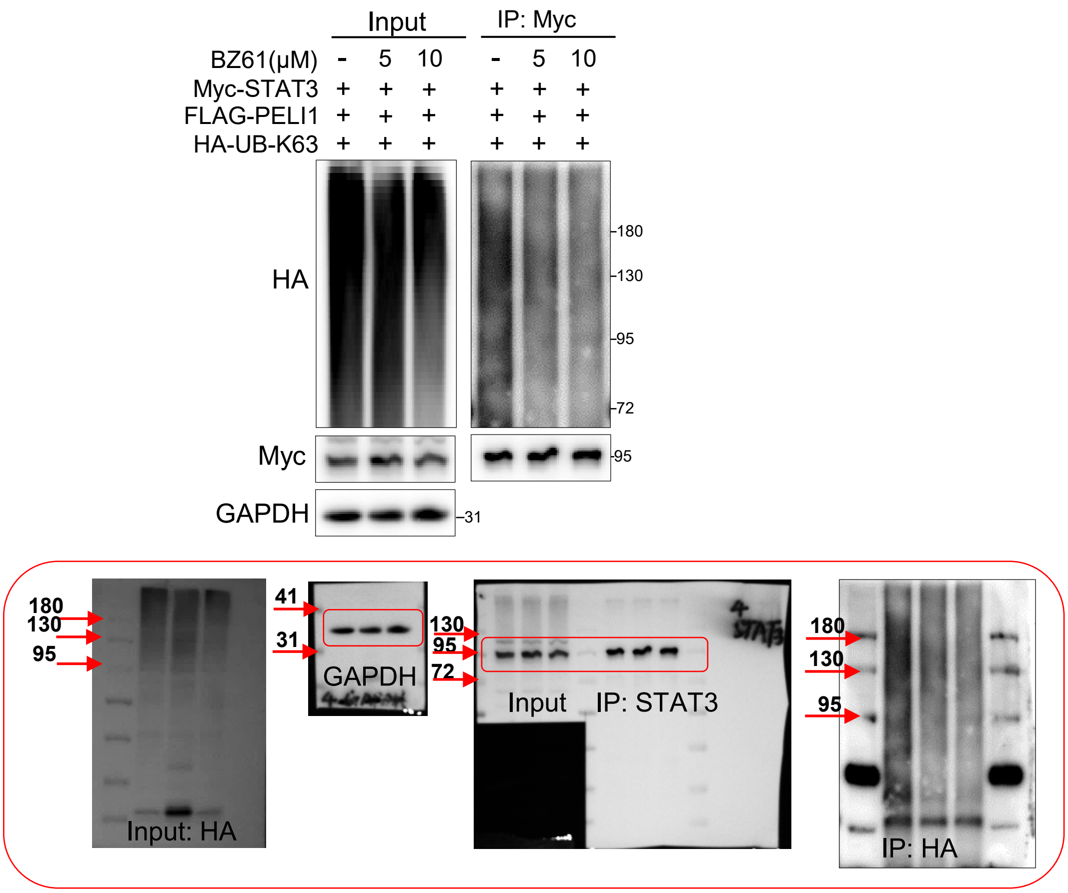

Suppl Fig.8F

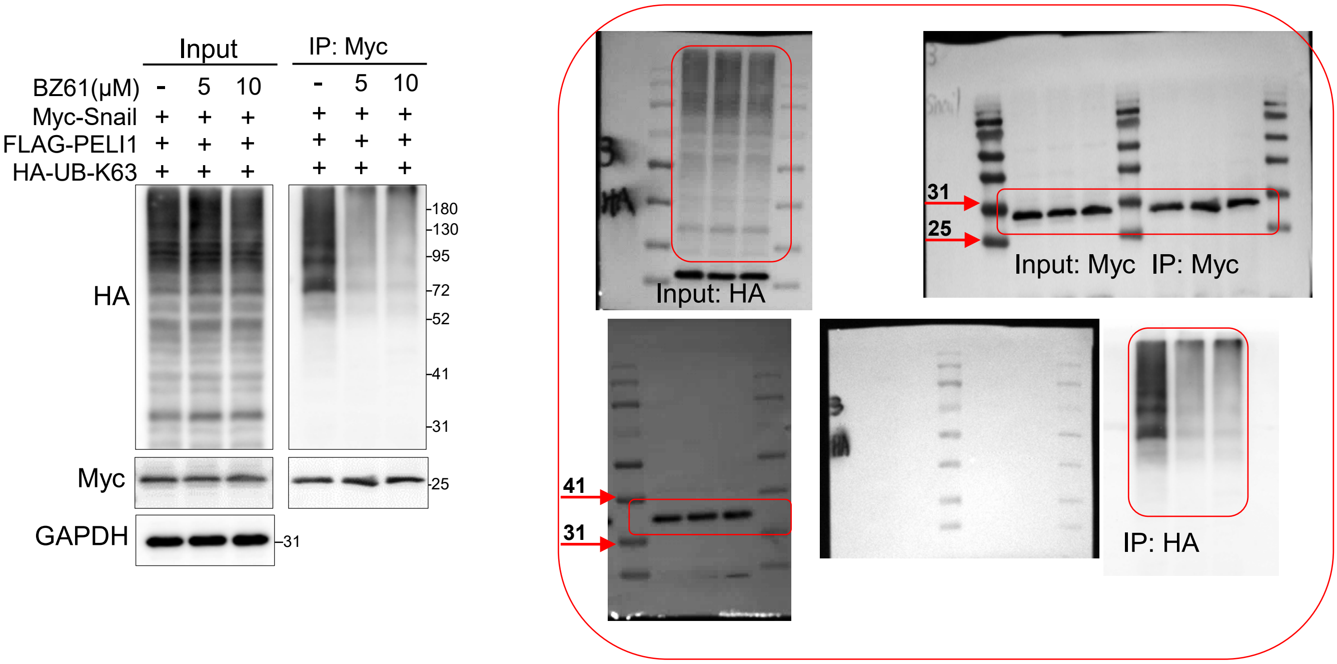

Suppl Fig.8G

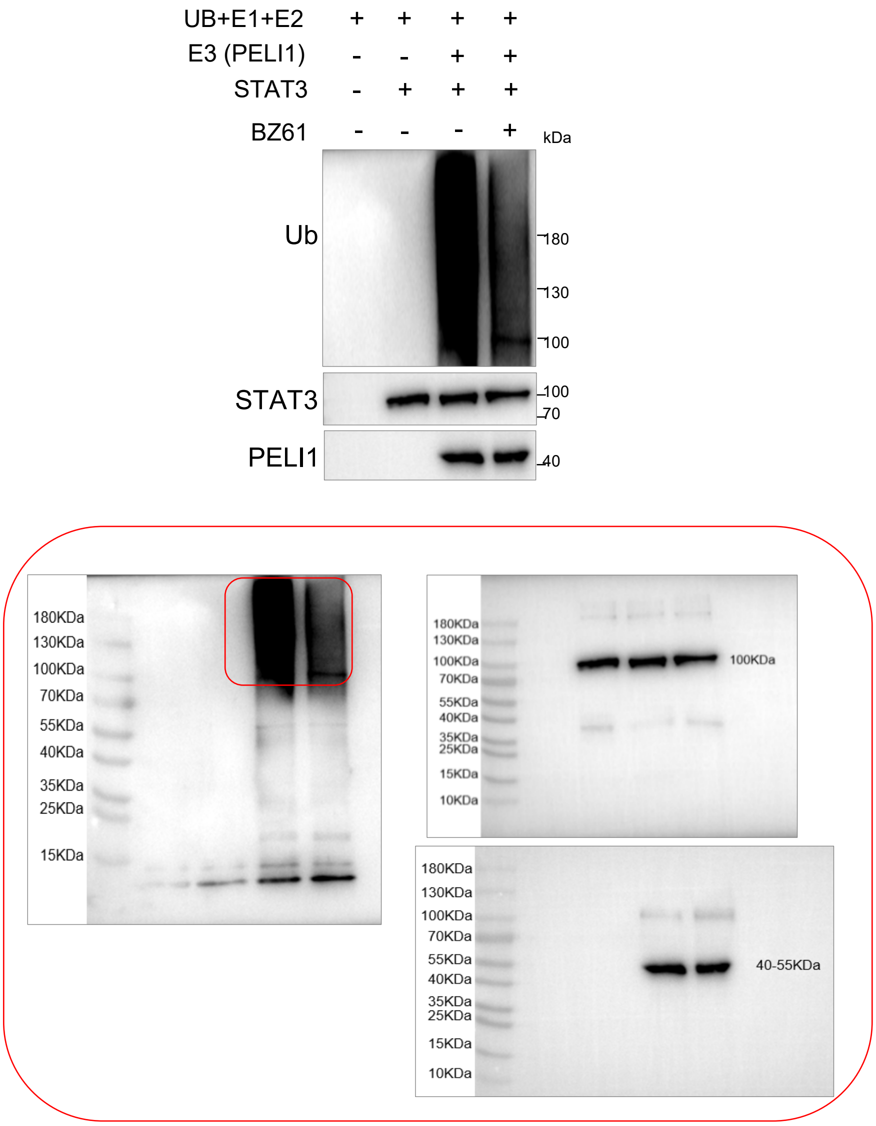

Suppl Fig.8H

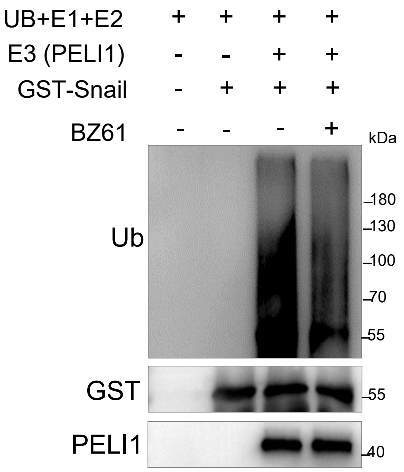

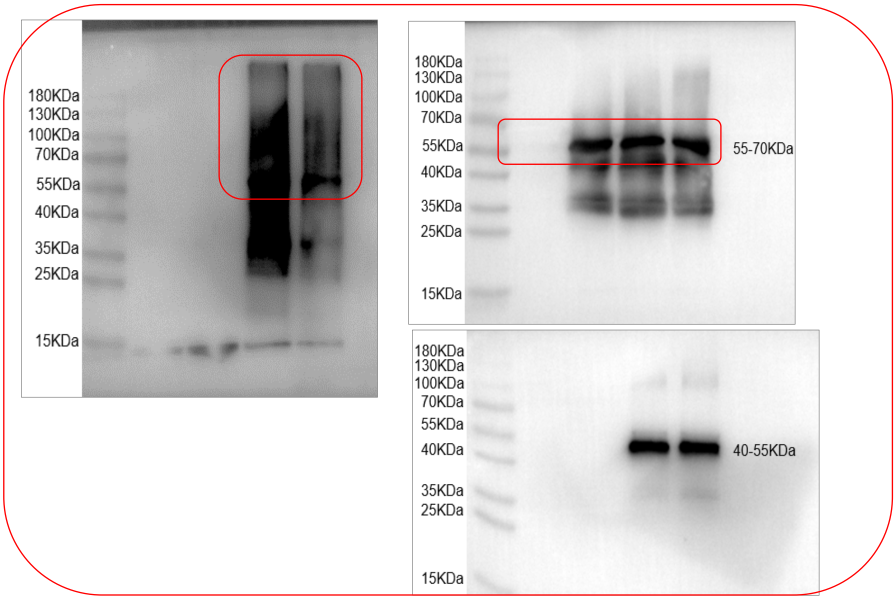

Suppl Fig.9B

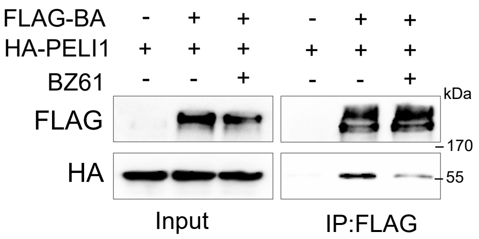

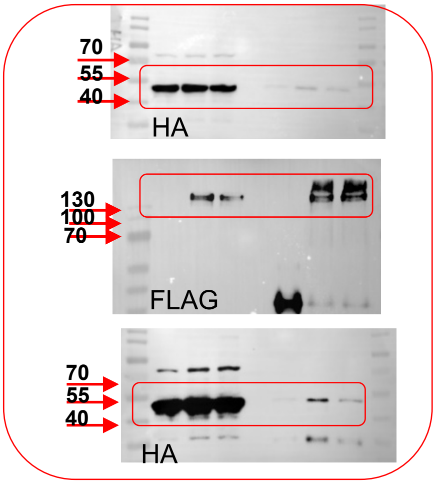

Supplement: Supplementary file 15 — Unedited blot and gel images [file 41419_2026_8799_MOESM15_ESM.pdf]
